# Transcriptomic and Zonal Signatures of Mitochondrial Peroxisomal Dysfunction in HCV Associates with Circulating Mitochondrial DNA Biomarkers

**DOI:** 10.3390/cimb48090877

**Published:** 2026-08-29

**Authors:** Moumita Chakraborty, Rownock Afruza, Maleeha F. Ahmad, Matthew G. Menkart, Jenna L. Oringher, Adekanyinsola Onitiri, Nicole Minerva, Kareen Akiva, Grace Zhang, Elizabeth C. Townsend, Gabriella Quinn, Anjali Rai, David E. Kleiner, Elliot Levy, Christopher Koh, Ohad Etzion, Rabab O. Ali, Theo Heller

**Affiliations:** 1 Translational Hepatology Section, National Institute of Diabetes and Digestive and Kidney Diseases, National Institutes of Health, Bethesda, MD 20892, USA; rownock.afruza@nih.gov (R.A.); mfahmad14@gmail.com (M.F.A.); mmenkar@emory.edu (M.G.M.); jloleigh12@gmail.com (J.L.O.); kanyinsolaonitiri3@gmail.com (A.O.); nicoleminerva757@gmail.com (N.M.); kareen.hill12@gmail.com (K.A.); grac3.zhang@gmail.com (G.Z.); ltowns22@gmail.com (E.C.T.); gabriella.m.quinn@gmail.com (G.Q.); anjali.rai@nih.gov (A.R.); ohadet34@yahoo.com (O.E.); rabab.ali226@gmail.com (R.O.A.); theoh@intra.niddk.nih.gov (T.H.); 2 Laboratory of Pathology, National Cancer Institute, National Institutes of Health, Bethesda, MD 20892, USA; kleinerd@mail.nih.gov; 3 Center for Interventional Oncology, Radiology and Imaging Sciences, Clinical Center, National Institutes of Health, Bethesda, MD 20892, USA; levyeb@cc.nih.gov; 4 Liver Diseases Branch, National Institute of Diabetes and Digestive and Kidney Diseases, National Institutes of Health, Bethesda, MD 20892, USA; christopher.koh@nih.gov

**Keywords:** chronic liver disease, hepatic zonation, cell free mitochondrial DNA, mitochondrial dysfunction, peroxisomal dysfunction, biomarkers of liver injury

## Abstract

Mitochondria and peroxisomes are critical for hepatic energy metabolism, lipid homeostasis, and reactive oxygen species (ROS) detoxification. In chronic hepatitis C virus (HCV) infection, continuous injury leads to cirrhosis; however, the spatial arrangement and reversibility of organelle dysfunction remain poorly understood. This study aimed to examine the zonal distribution of mitochondrial and peroxisomal injury in liver biopsies and elucidate the role of circulating cell-free mitochondrial DNA (ccfDNA) in patients with chronic HCV and cirrhosis following antiviral therapy. We employed advanced microscopy imaging and transcriptomic analysis of liver biopsies and quantified ccf-mtDNA as a noninvasive marker of mitochondrial injury in the peripheral blood of these patients. Transcriptomic data revealed alterations in mitochondrial and peroxisomal pathway alterations in HCV-infected patients. The imaging data displayed distinct zone-specific patterns of organelle damage. Following viral removal, significant improvement in mitochondrial and peroxisomal protein expression were noted, indicating partial recovery of organelle integrity following viral clearance; whether this reflects true subcellular regeneration or an early stage of a longer recovery process remains to be determined. Additionally, we showed that ccf-mtDNA quantitatively reflects intrahepatic mitochondrial dysfunction, indicating its potential as a diagnostic biomarker in therapeutic approaches. These findings indicate that organelle injury in chronic HCV is spatially patterned, disease severity-dependent, and partially reversible following antiviral therapy.

## 1. Introduction

The liver is a central organ involved in metabolic homeostasis, detoxification, and energy production. Mitochondria and peroxisomes play pivotal roles in maintaining liver function and are crucial organelles for cellular metabolism [1]. Mitochondria are central to energy production and regulation of apoptosis, whereas peroxisomes contribute to lipid metabolism and reactive oxygen species (ROS) detoxification. Dysregulation of organelle function can exacerbate liver disease. A recent study showed disruption of energy metabolism in the gut–liver axis of patients with hepatitis C infection (HCVi) [2]. Transcriptomic analyses have revealed that the pathways downregulated in HCVi were metabolic, including the metabolism of fatty acids and amino acids, in addition to peroxisome proliferator-activated receptor (PPAR) signaling [2]. Importantly, the function of the genes downregulated in HCVi were localized predominantly to the peroxisomes and mitochondria and were fundamental for energy and redox balance.

Zonal organization of the liver, or hepatic zonation, is the spatial distribution of metabolic functions across the liver acinus zones, which are classified into periportal (zone 1), midlobular (zone 2), and pericentral (zone 3) regions [3]. This is driven by differential gene expression regulated by oxygen gradients, hormones, and other signaling molecules [4]. Hepatitis C virus (HCV) core and nonstructural proteins induce oxidative stress and disrupt metabolic zonation by altering the expression of genes involved in oxidative phosphorylation and detoxification [5]. Moreover, HCV infection induces epigenetic changes, such as DNA methylation and histone modification, which alter the gene expression required to maintain hepatic zonation. These perturbations lead to loss of zonal gene expression patterns, contributing to metabolic dysfunction [6,7]. Recent evidence suggests that HCV infection disrupts hepatic zonation and contributes to liver disease progression [8].

HCV infection has been associated with changes in peroxisomal function, including alterations in peroxisomal biogenesis and proliferation, which may disrupt hepatic metabolism and compromise the ability of the liver to counteract viral infection [9,10]. Hepatic zonation may influence the differential regulation of peroxisomes in response to HCV infection, leading to altered lipid metabolism, increased oxidative stress, and a compromised antiviral response [11].

Recent studies have highlighted that hepatic zonation influences the extent of mitochondrial dysfunction, with periportal regions being more susceptible to dysfunction [12]. Mitochondrial dysfunction in HCV infection has been linked to impaired oxidative phosphorylation, excessive ROS production, and disruption of mitochondrial dynamics and biogenesis [13,14,15,16,17]. These mitochondrial alterations can exacerbate liver injury, foster a proinflammatory environment, and support viral persistence in the liver.

In liver disease, cell-free DNA (cfDNA), especially circulating cell-free mitochondrial DNA (ccf-mtDNA), is released during hepatocyte stress, apoptosis, or necrosis and can serve as an indicator of general mitochondrial stress. Given the central role of mitochondria in metabolic regulation and redox homeostasis, increased circulating mtDNA levels may indicate impaired mitochondrial integrity and contribute to disease progression [18,19]. Therefore, measuring cf-mtDNA provides a noninvasive strategy to monitor hepatocellular injury, assess disease progression, and potentially evaluate therapeutic responses in HCV infection.

Mitochondrial and peroxisomal dysfunction in HCV infection is known, but the distribution of liver acinus before and after viral clearance remains unclear. As hepatic zonation affects metabolic specialization and susceptibility to injury, understanding these spatial changes is crucial. This study examined the zonal interaction between mitochondrial dysfunction and peroxisomal changes during HCV infection and post therapy recovery. Integrating these with biomarkers, such as ccf-mtDNA aims to link organelle injury with noninvasive stress indicators. This may help to identify targetable metabolic pathways, thereby enhancing HCV-associated liver disease monitoring and therapy.

## 2. Materials and Methods

### 2.1. Patient Selection

As previously described, 29 patients were evaluated for chronic HCV-associated liver disease at the National Institutes of Health Clinical Center and the exclusion criteria were specified (NCT02400216) [2,20]. Positive anti-HCV antibody and HCV RT-PCR results are required to confirm a chronic HCV infection. All patients signed a consent form for participation in the Institutional Review Board-approved protocol. All 29 patients underwent initial evaluation and responded to sofosbuvir/velpatasvir. Twenty-three patients returned after six months of sustained virologic response (SVR). Patients with hepatocellular carcinoma were excluded from this study. Patients with an Ishak fibrosis score (IF) of 0–4 were considered non-cirrhotic (NC), and those with an Ishak fibrosis score of 5–6 were considered cirrhotic (C). At the HCVi evaluation, 16 patients were classified as NC and 13 as C. At the SVR evaluation, 14 patients were classified as NC and 9 as C. Because tissue and sample availability differed among experimental assays, the number of patients included in individual analysis varied. The demographic and clinical characteristics as well as sample distribution of the study cohort are summarized in Table 1. This table is modified from our previous study [21].

### 2.2. Transcriptomic Analysis of Mitochondrial and Peroxisomal Genes

Transcriptional profiling was performed based on paired liver biopsy samples from the same patient cohort mentioned in Section 2.1, Table 1, during and after HCV infection with SVR status. The 23 individuals who returned to follow-up lacked one paired RNA sequencing data for transcriptional profiling because of poor tissue samples. Paired transcriptional analyses were performed on the 22 HCVi-SVR pairs. As previously described [2], total RNA was extracted from snap-frozen liver tissues using Thermo Fisher Scientific TRIzol reagent (Catalog No. 15596026), MA, USA and a Qiagen RNA extraction kit (Catalog No. 74104), MD, USA. RNA samples were then subjected to poly A selection using the New England Biolabs NEBNext Poly (A) Selection Kit (Catalog No. E7490S), MA, USA to exclude RNA molecules lacking a poly A tail. A cDNA library was generated from the selected RNA library using the New England Biolabs ScriptSeq RNA Library Prep Kit (Catalog No. SSV221106), MA, USA. All cDNA libraries were quantified using the KAPA Biosystems Illumina qPCR kit (Roche Catalog No. 07960140001) IN, USA, normalized, and submitted to the NIDDK Genomics Core for sequencing on an Illumina HiSeq 4000 platform. The raw sequence files were subsequently aligned to the Homo sapiens *hg38* reference genome using the STAR algorithm on the Partek Flow platform. Of the 24,380 identified genes, 1094 were selected for further analysis. Seventy-eight of these genes were identified as part of the peroxisomal biogenesis pathway according to KEGG, and 1102 genes were identified as nuclear genes related to mitochondrial function according to MitoCarta 3.0 (Broad Institute). Batch effect removal, pre-analysis data filtering, and differential gene expression analysis were performed using the DESeq2 R package. Three differential gene expression analyses were performed: (1) comparing HCVi with SVR in all patients, (2) comparing HCVi with SVR in patients with cirrhosis only, and (3) comparing HCVi with SVR in patients without cirrhosis only. Differentially expressed genes (DEGs) were quantified as significantly upregulated or downregulated if the false discovery rate (FDR) was <0.1 and |log2FC| > 0.5. The transcriptomic data analyzed in this study were previously published [2] and are publicly available through the NCBI Bioproject database under accession number PRJNA727609.

### 2.3. Immunofluorescent Staining

Immunofluorescence analysis was conducted on unpaired liver biopsy tissues because of tissue availability and quality control requirements. These data were obtained from the same patient cohort described in Section 2.1, Table 1. The four subgroups were: (1) HCVi cirrhotic, *n* = 10; (2) HCVi non-cirrhotic, *n* = 13; (3) SVR cirrhotic, *n* = 5; (4) SVR non-cirrhotic, *n* = 11. ATP5A, PMP70, and catalase were selected as marker genes for mitochondria and peroxisomes owing to their proven involvement in energy production in mitochondria, peroxisome membranes, and antioxidation activities in peroxisomes, respectively. The selected markers were used to examine whether transcriptome changes seen in the mitochondria and peroxisomes were correlated with any corresponding protein changes in the liver. Samples were formalin-fixed and paraffin-embedded. Tris-EDTA buffer (Catalog No: ab93684) (Abcam) was used for antigen retrieval. Sections were incubated overnight at 4 °C in a primary antibody solution of Anti-ATP5A (Catalog No: ab14748) at a final concentration of 1:250 in 1% bovine serum albumin (BSA), Anti-catalase (Catalog No: ab16731), and Anti-PMP70 (Catalog No: ab211533) each at a final concentration of 1:100 in 1% BSA. The sections were incubated for 1 h at room temperature with a secondary antibody solution of Alexa Fluor 488 (Catalog No: ab150115) and Alexa Fluor 555 (Catalog No: ab150078), at a final concentration of 1:1000 in 1% BSA. Autofluorescence quenching was performed first withVector Laboratories True Black (Catalog No: 23007), CA, USA, then (Biotium), CA, USA for 30 s, followed by TrueView (Catalog No: SP-8400) (Vector Laboratories), CA, USA treatment for 5 min. Nuclear counterstaining was performed using DAPI (Catalog No: D21490) (Thermo Fisher Scientific), MA, USA (1:1000 in PBS) for 10 min. A Thermo Fisher Prolong Diamond Antifade Mountant (Catalog No: P36970) was added prior to the coverslip application.

### 2.4. Confocal Imaging and Analysis

The sections were imaged using a 20x objective on an LSM 780 confocal microscope (Zeiss, Oberkochen, Germany) using Zeiss ZEN 2.3 SP1 software. Images were analyzed using the FIJI plugin LABKIT (ImageJ 1.53p) to perform image segmentation and determine the particle count in hepatocytes for the mitochondrial marker ATP5A and the peroxisomal markers catalase and PMP70. All images were normalized to the total number of nuclei. The Mann–Whitney U test (*p* < 0.05) was used to compare markers between the groups.

### 2.5. Circulating Cell Free DNA (ccfDNA) Extraction and Quantification

Peripheral blood samples were collected from the same patient cohort as described in Section 2.1, Table 1, with HCV infection and after SVR. Whole blood was centrifuged at 1600 rpm for 10 min at 4 °C to isolate the plasma. Then, 1 mL of plasma was aliquoted into multiple Eppendorf tubes, and all samples were stored at −80 °C. A total of 500 µL of frozen plasma from peripheral samples was processed using the QIAamp MinElute ccfDNA kit (QIAGEN, catalog no: 55284) to extract ccfDNA. ccfDNA was then amplified using primers for human mitochondrial (MT) genes, such as mitochondrially encoded NADH: ubiquinone oxidoreductase core subunit 1 (MT-ND1), mitochondrially encoded NADH dehydrogenase (MT-ND6), mitochondrially encoded cytochrome C oxidase III (MT-COXIII), and the nuclear gene β-actin (housekeeping gene). The primers used for amplification are listed in Table 2. The amplified product was quantified using reference cDNA (Takara catalog no: 639653) standard curves and copy numbers were calculated using the Y-intercept and slopes, as described previously [22]. Moreover, 2% agarose gel electrophoresis was performed to confirm an amplicon length of 103 base pairs (bp) for MT-COXIII, 69 bp for MT-ND1, 90 bp for MT-ND6, and 135 bp for β-actin. Samples that did not contain valid housekeeping gene data were excluded from further analysis. The following four subgroups were analyzed for ccfmtDNA copy numbers: (1) HCVi cirrhotic, *n* = 13; (2) HCVi non-cirrhotic, *n* = 16; (3) SVR cirrhotic, *n* = 7; (4) SVR non-cirrhotic, *n* = 14.

### 2.6. Statistical Analysis

Statistical analyses were performed using GraphPad Prism 9.0 and R software version 4.3.1. Given the relatively small and unequal subgroup sizes in different comparisons and considering that the biological datasets investigated are unlikely to have a normal distribution because of their inherent potential for containing outliers (including immunofluorescence particle count and ccfmtDNA copy number), non-parametric statistics were used for all calculations conducted in the study. For comparisons among more than two independent groups, the one-way ANOVA was used, followed by Bonferroni correction. Two-sided Mann–Whitney U tests were retained only for direct two-group comparisons for unpaired samples, and the Wilcoxon test was used for paired samples. All *p*-values were two-tailed and corrected for false discovery rate (FDR), where relevant. Statistical significance was defined as *p* < 0.05 or, for multi-group comparisons, as Bonferroni adjusted *p* < 0.05.

## 3. Results

### 3.1. Modulation of Mitochondrial and Peroxisomal Pathways Before and After HCV Clearance

**Figure 1:**
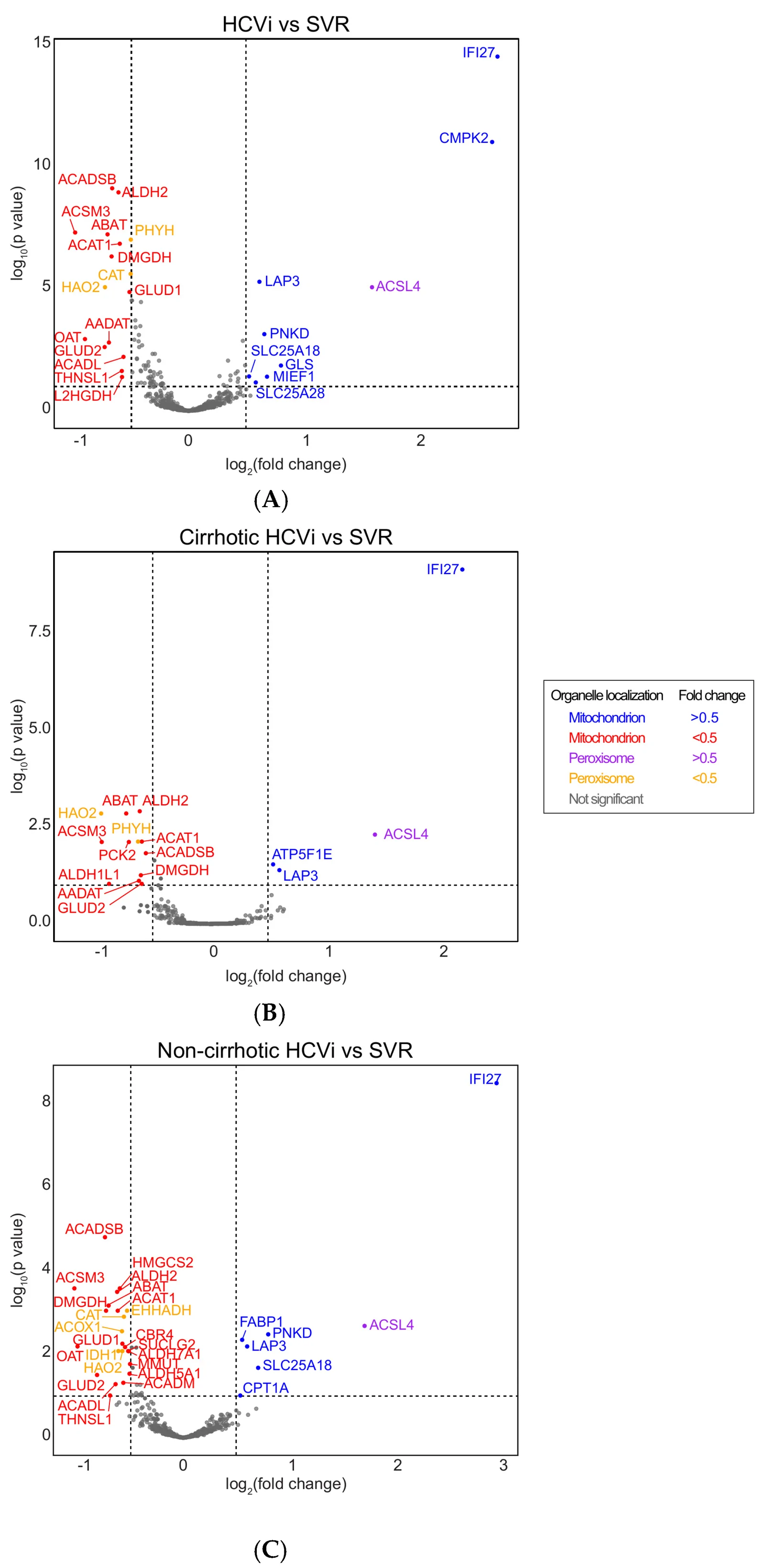
Volcano plots of differential gene expression analysis of mitochondrial and peroxisomal genes between 22 paired samples comparing (**A**) HCVi relative to SVR in all patients, (**B**) HCVi relative to SVR only in patients with cirrhosis, and (**C**) HCVi relative to SVR only in patients without cirrhosis. Each dot represents one gene. Colored genes are significant, while gray genes are not significant.

We have previously [2] showed that genes downregulated in HCVi cells encode proteins almost exclusively localized to peroxisomes and mitochondria, which are organelles responsible for energy and redox balance in the cell. To determine alterations in the mitochondrial and peroxisomal pathways before and after HCV clearance, the differential gene expression between HCVi and SVR was analyzed using DESeq2. Of the 24,380 genes identified, 1180 genes were further analyzed as genes related to mitochondrial or peroxisome function. A total of 1102 genes were identified as mitochondrial nuclear genes according to Mito Carta 3.0 (Broad Institute) (https://www.broadinstitute.org/mitocarta/mitocarta30-inventory-mammalian-mitochondrial-proteins-and-pathways, accessed on 26 August 2026), and 78 peroxisomal genes were identified using KEGG. Figure 1 shows all mitochondrial and peroxisomal genes that were differentially expressed in these groups. A complete list of differentially expressed genes (DEGs) specific to mitochondria and peroxisomes for each group, including associated statistical values, is provided in Supplementary Table S1. Acyl-CoA Dehydrogenase Long Chain *(ACADL)*, which plays a role in mitochondrial fatty acid oxidation, was downregulated in the HCVi group compared to the SVR group as well as in the non-cirrhotic group. Carbonyl reductase 4 *(CBR4)* is downregulated in non-cirrhotic HCVi compared to SVR, which may be associated with oxidative stress and damage. Hepatic transcriptomic analysis of mitochondrial acyl-CoA synthetase medium-chain family member 3 *(ACSM3)* was downregulated in all three groups (HCVi vs. SVR/cirrhotic, HCVi vs. SVR/non-cirrhotic, and HCVi vs. SVR). Interestingly, phytanoyl-CoA 2-hydroxylase (*PHYH*), which is primarily known for peroxisomal alpha-oxidation, was downregulated in the HCVi vs. SVR/cirrhotic HCVi vs. SVR comparison. In contrast, catalase (*CAT*), responsible for hydrogen peroxide (H_2_O_2_) conversion, was also downregulated in HCVi vs. SVR/non-cirrhotic HCVi vs. SVR. Moreover, acyl-coA oxidase 1 (*ACOX1*), which is responsible for fatty acid β-oxidation, was downregulated in non-cirrhotic HCVi compared to SVR. This may be because HCV disrupts host lipid metabolism to facilitate its replication, leading to metabolic dysfunction and oxidative stress. These results confirm that there is a distinct alteration in mitochondrial and peroxisomal genes before and after HCV clearance, which mainly affects energy metabolism.

#### 3.1.1. Mitochondrial and Peroxisomal Marker Expression Is Modulated Across Zones 1 and 2 in Patients with Cirrhosis

**Figure 2:**
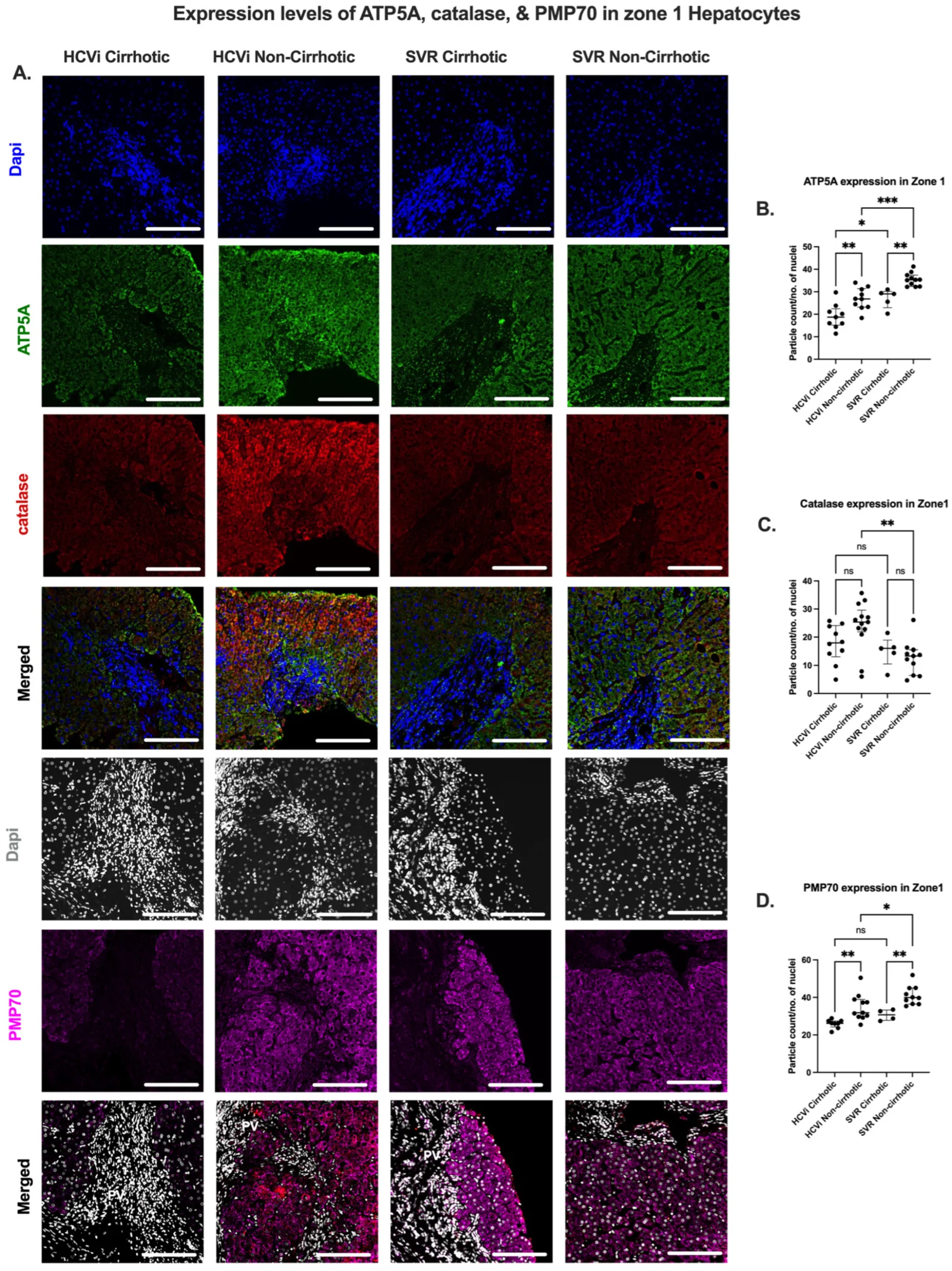
Immunostaining images of the liver biopsies showing expression levels of mitochondria and peroxisome markers in zone 1 hepatocytes. (**A**) Images showing confocal laser scanning micrographs stained with mitochondrial marker ATP5A (green), peroxisomal marker catalase (red), PMP70 (purple), primary antibodies and nuclear marker Dapi (blue) in hepatic zone 1 of HCVi cirrhotic (*n* = 10), HCVi non-cirrhotic (*n* = 13), SVR cirrhotic (*n* = 5) and SVR non-cirrhotic (*n* = 11) individuals. (**B**–**D**) Graphs show quantified particle count in the zone 1 hepatocytes for ATP5A, catalase and PMP70 expression across HCVi cirrhotic, HCVi non-cirrhotic, SVR cirrhotic and SVR non-cirrhotic individuals. All the images were normalized by the total number of nuclei. One-way ANOVA followed by Bonferroni correction (*p* < 0.05) was used for marker comparison between groups. ns: non-significant. * *p* < 0.05, ** *p* < 0.01, *** *p* < 0.001.

**Figure 3:**
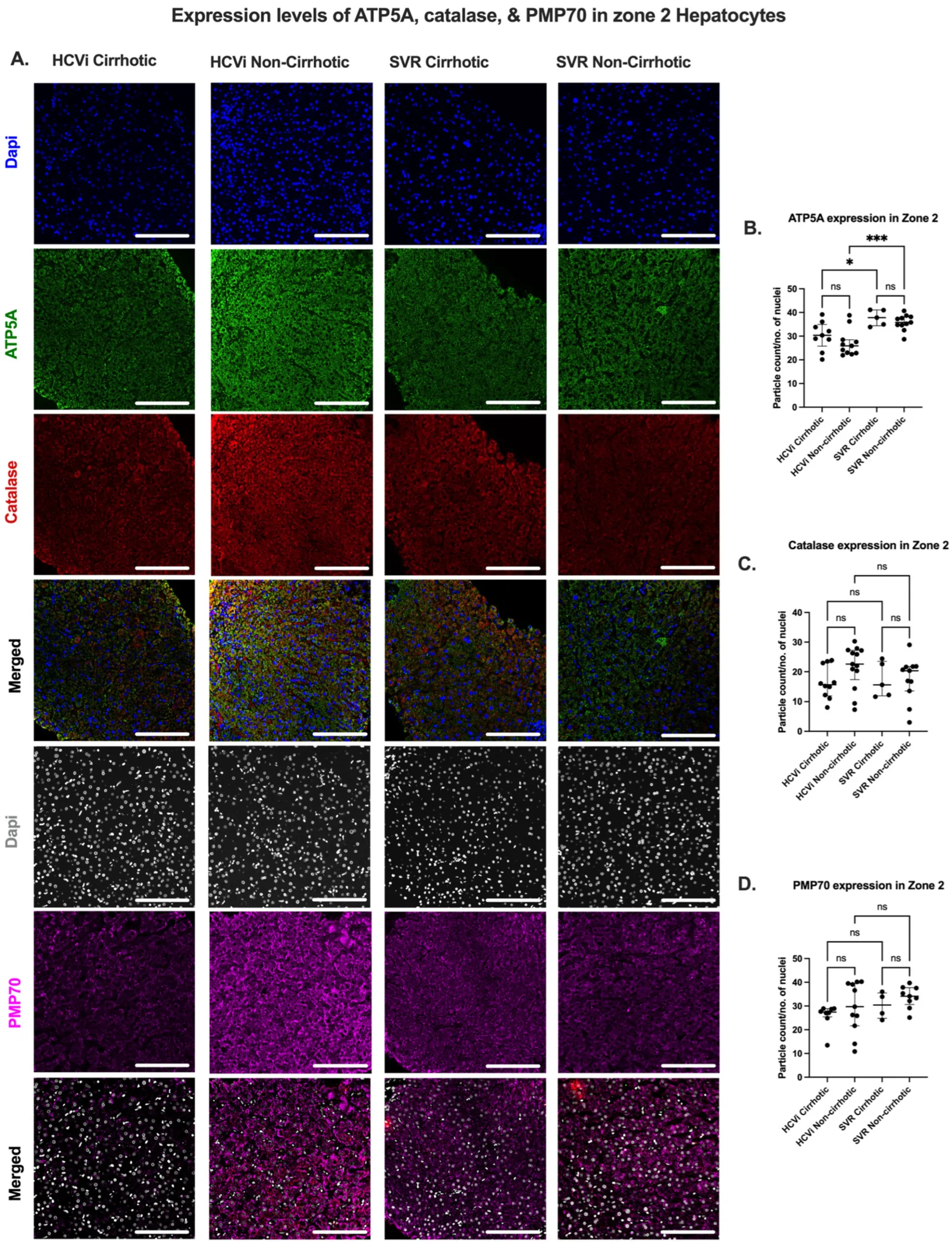
Immunofluorescent labeling of the liver biopsies quantifying the levels of mitochondria and peroxisome markers in zone 2 hepatocytes. (**A**) Confocal images stained with mitochondrial marker ATP5A (green) and peroxisomal markers catalase (red), PMP70 (purple), primary antibodies and nuclear marker Dapi (blue) in hepatic zone 2 of HCVi cirrhotic (*n* = 10), HCVi non-cirrhotic (*n* = 13), SVR cirrhotic (*n* = 5) and SVR non-cirrhotic (*n* = 11) individuals. (**B**–**D**) Graphs depict quantified particle count in zone 2 hepatocyte for ATP5A, catalase and PMP70 expression across HCVi cirrhotic, HCVi non-cirrhotic, SVR cirrhotic and SVR non-cirrhotic. Normalization was done with the total number of nuclei. *p*-values were calculated using one-way ANOVA followed by Bonferroni adjustment. ns: non-significant. * *p* < 0.05, *** *p* < 0.001.

Given the alteration of hepatic mitochondrial and peroxisomal genes in HCVi and SVR, we aimed to determine the changes in protein levels across specific liver zones before and after HCV clearance in cirrhotic and non-cirrhotic individuals. Immunofluorescence images of HCVi and SVR liver biopsies stained with ATP5A (green), catalase (red), PMP70 (purple), and DAPI (blue/gray). HCVi cirrhotic patients showed significantly reduced expression of ATP5A (*p* = 0.002) and PMP70 (*p* = 0.004) but no change in catalase expression in zone 1 hepatocytes (Figure 2A–D), whereas no significant difference was observed in zone 2 hepatocytes (Figure 3A–D) compared with HCVi non-cirrhotic patients. After HCV clearance, SVR cirrhotic individuals, when compared with SVR non-cirrhotic individuals, again demonstrated reduced ATP5A (*p* = 0.005) and PMP70 (*p* = 0.008) expression in zone 1 hepatocytes (Figure 2A–D), with no significant changes in catalase expression levels. Moreover, no significant changes in the expression patterns of these three markers were observed in zone 2 hepatocytes (Figure 3A–D). These results suggest that ATP5A, catalase, and PMP70 in patients infected with HCV and SVR individuals were affected in a zonal pattern, indicating the greatest perturbations in zone 1, the most metabolically active area, and in patients with cirrhosis.

#### 3.1.2. Mitochondrial and Peroxisomal Status Improves in Zone 1 Hepatocytes After DAA Therapy

The expression of the mitochondrial marker ATP5A (*p* = 0.01) was significantly higher in zone 1 of SVR cirrhotic patients than in HCVi cirrhotic patients. No significant changes were observed in the peroxisomal marker catalase and PMP70 across zone 1 when comparing SVR cirrhotic patients with HCV cirrhotic patients (Figure 2A–D). Similarly, only ATP5A expression (*p* = 0.03) was significantly increased across zone 2 in SVR cirrhotic patients after HCV clearance (Figure 3A–D). Moreover, in HCV non-cirrhotic and SVR non-cirrhotic patients, there was a significant increase in ATP5A (*p* = 0.0005) and PMP70 (*p* = 0.04) levels in zone 1 hepatocytes. However, catalase expression was still low compared with that in HCVi non-cirrhotic patients in zone 1 hepatocytes (Figure 2A–D). In zone 2 hepatocytes, only ATP5A expression (*p* = 0.007) was significantly higher, and no such changes in PMP70 or catalase were observed after HCV clearance (Figure 3A–D). These results suggest that post DAA therapy, the expression levels of ATP5A and PMP70 in zone 1 increase, indicating improved mitochondrial and peroxisomal functions after HCV clearance. Globally, ATP5A (*p* = 0.0001) and PMP70 (*p* = 0.01) expression levels increased, whereas catalase expression decreased (*p* = 0.001) after DAA therapy (Supplementary Figure S1), possibly because of the morphological changes in peroxisomes [23]. The partial recovery of organelle marker expression after DAA therapy suggests that mitochondrial and peroxisomal injury in chronic HCV is, at least in part, reversible, and highlights these organelles as potential therapeutic targets; however, catalase expression remained below HCVi levels even after SVR, indicating that recovery was incomplete at 6 months and may require a longer time course.

#### 3.1.3. Copy Numbers of Circulatory Cell-Free mtDNA Increased During HCVi Compared to SVR

**Figure 4:**
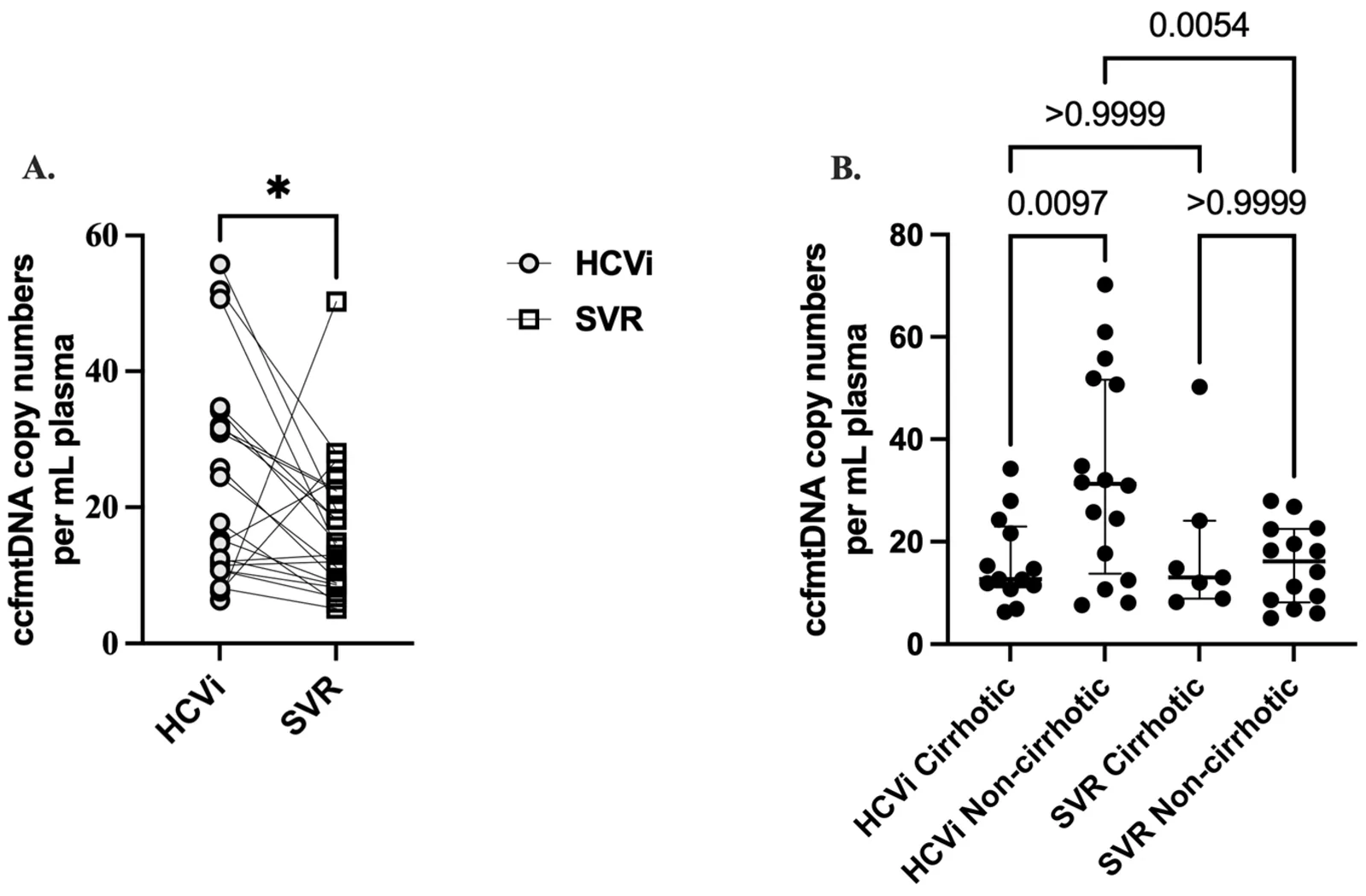
ccfmtDNA copy numbers in patients with HCV infection following SVR. ccfDNA was extracted from 500 μL of plasma and quantified using TBS-380 Mini-Fluorometer. Representative samples were run on an agarose gel to verify the product size. β-actin was used as the control gene. (**A**) Individual ccfmtDNA copy numbers of HCVi compared to SVR. (**B**) ccfmtDNA copy numbers in all four groups, (1) HCVi cirrhotic, (2) HCVi non-cirrhotic, (3) SVR cirrhotic, and (4) SVR non-cirrhotic. *p*-values calculated using Wilcoxon test for paired samples and multiple comparisons using one-way ANOVA followed by the Bonferroni correction was performed. * *p* < 0.05.

Given the mitochondrial derangement in liver biopsies of patients with HCVi, we sought to identify a noninvasive marker. Therefore, we evaluated the ccfmtDNA status during HCV infection and post clearance in cirrhotic and non-cirrhotic patients. The ccfmtDNA copies were significantly higher in HCVi than in SVR (*p* = 0.02) (Figure 4A), suggesting infection-mediated mitochondrial breakdown. During HCVi, patients with cirrhosis had significantly lower ccfmtDNA copies than non-cirrhotic patients (*p* = 0.009) (Figure 4B), suggesting that healthy mitochondria maintain ccfmtDNA, whereas cirrhotic livers do not. This finding was further supported by a significant reduction in ccfmtDNA copy numbers after SVR (*p* = 0.005) in non-cirrhotic patients. No differences in ccfmtDNA copy number were noted in cirrhotic patients during or after HCV infection (Figure 4B), indicating that both HCV infection and liver fibrosis affect ccfmtDNA levels, with cirrhotic mitochondria possibly being unable to release ccfmtDNA. We compared mtDNA genes between the non-cirrhotic and cirrhotic groups during HCVi and SVR. During HCV infection, the copy numbers of MT-ND1, MT-ND6, and MT-COXIII were significantly higher (*p* = 0.05, 0.03, and 0.01, respectively) than those in SVR (Supplementary Figure S2A). All individual ccfmtDNA copy numbers were significantly higher (*p* < 0.05) in non-cirrhotic patients than in cirrhotic patients (Supplementary Figure S2B) during infection. After SVR, no changes in ccfmtDNA copy number were observed between the groups (Supplementary Figure S2B). These results indicate that HCV infection impairs mitochondrial integrity and/or function, as infected patients have higher copy numbers of ccfmtDNA than SVR patients, with a major decrease in non-cirrhotic patients post clearance. It is crucial to understand why mitochondria in the cirrhotic liver do not appear to respond to HCV infection in the same manner and seem to release less ccfmtDNA, suggesting a “burnt out” state impacting the changes observed in both transcriptional analysis and confocal microscopy presented above, which is relevant for the metabolic changes observed in cirrhosis.

## 4. Discussion

We built on previous data by providing detailed transcriptomic evidence for mitochondrial and peroxisomal gene dysregulation during HCV infection and cirrhosis. These findings revealed the molecular mechanisms underlying HCV-induced liver pathology and suggested potential therapeutic targets. Genes such as *ACADL*, *CBR4*, *ACSM3*, *PHYH*, *CAT*, and *ACOX1* were downregulated in the HCV vs. SVR cirrhotic and non-cirrhotic groups, suggesting their potential roles in liver health and disease [24,25,26,27,28,29]. The significant dysregulation of mitochondrial and peroxisomal genes in chronic HCV infection and cirrhosis provides potential targeted therapeutic approaches to ameliorate cirrhosis in SVR, with implications for the development of hepatocellular carcinoma [30] and possibly other liver diseases.

The interplay between peroxisomes and mitochondria is vital for maintaining cellular redox balance [1,31,32] and peroxisomes have been shown to regulate mitochondrial function [33]. Our findings of concurrent mitochondrial and peroxisomal dysfunction highlight a potential synergistic effect in exacerbating oxidative stress and liver damage and offer a broader perspective on the metabolic disruptions that occur in chronic HCV infection and cirrhosis.

This study elucidated the zonal distribution of mitochondrial and peroxisomal damage during chronic HCV infection. Our findings indicated a pronounced gradient of mitochondrial and peroxisomal damage across the hepatic zones, with the periportal region (zone 1) exhibiting the most severe alterations during HCVi infection. Diminished mitochondrial expression in the periportal zone was characterized by significantly reduced ATP5A expression in cirrhotic patients compared with that in non-cirrhotic patients. The small sample size of the pericentral (zone 3) region made it difficult to draw conclusions about this region. This zonal heterogeneity is consistent with previous studies that have highlighted the metabolic specialization of hepatic zones and their differential susceptibility to various insults [4,6,34,35]. Preferential damage to the periportal zone can be attributed to several factors. First, the higher oxygen tension in the periportal region facilitates oxidative phosphorylation but also predisposes hepatocytes to oxidative damage, especially during chronic inflammatory conditions, and likely exacerbates fibrosis progression [36,37,38,39]. Second, periportal hepatocytes produce a substantial amount of ROS as part of their detoxification role, which can overwhelm antioxidant defenses and lead to further damage [36]. Finally, the spatial distribution of HCV receptors and the propensity of the virus to infect periportal hepatocytes may contribute to the observed zonal disparities in organelle damage [40]. Peroxisomes, which are essential for lipid metabolism and ROS detoxification, also cause significant damage to the periportal region of the liver. The decreased expression of PMP70 and catalase in cirrhotic patients compared to that in non-cirrhotic patients suggests a compromised capacity to mitigate oxidative damage, further accentuating the vulnerability of periportal hepatocytes. Further research should explore the molecular mechanisms driving zonal organelle damage differences, recovery and the long-term outcomes of viral clearance on hepatic function.

Interestingly, the observed partial recovery of both organelles after viral clearance is consistent with the liver’s capacity for metabolic recovery following removal of a chronic injurious stimulus; however, given the catalase levels remained reduced at 6 months post SVR and that cirrhotic patients showed limited recover overall, full organelle regeneration likely requires a longer interval than examined here, or may not occur in patients with established fibrosis [41,42]. These insights reinforce the importance of early antiviral intervention and the remarkable capacity of the liver to repair. Therefore, antioxidants that specifically localize to mitochondria, such as MitoQ and SkQ1, could potentially ameliorate oxidative stress and protect against mitochondrial dysfunction [43]. Strategies to enhance peroxisome biogenesis and function may be beneficial for peroxisome dysfunction. PPAR agonists, key regulators of lipid metabolism and peroxisome proliferation, may restore peroxisomal functions and mitigate lipid metabolic disturbances and are increasingly being utilized in the treatment of multiple liver diseases [44,45,46,47].

Recent findings have shown that the circadian clock helps govern liver metabolism. Disrupting it has been linked to steatotic liver disease, fibrosis, and viral hepatitis. Using human liver chimeric mice, Mukherji, Jühling, and colleagues showed that chronic HCV infection disrupts the rhythmicity of more than 1000 hepatocyte genes and alters the diurnal epigenome, with the pathways governing fatty acid metabolism, peroxisome organization, and fibrosis among those most affected [48]. These rhythmic disturbances remained abnormal in patients with advanced liver disease and were only partially reversed after viral clearance in both HCVi and DAA cured patients suggesting that HCV-induced circadian disruption can outlast SVR. Because the CC transcriptionally regulates both mitochondrial biogenesis and peroxisomal proliferation, it may contribute directly to the zonal, cirrhosis-dependent organelle injury we describe here; incomplete resolution of circadian dysregulation after SVR could also explain why the organelle markers we observed recovered only partially, not completely. Testing this hypothesis directly would require circadian gene expression data from biopsies taken at different times of day. This is harder to do in patients than in mice: repeat morning and evening biopsies from the same person are rarely feasible, so whether CC targeting strategies could complement antiviral therapy in restoring hepatic organelle and metabolic homeostasis remains an open question.

Given the aforementioned mitochondrial derangement, we investigated the levels of circulating ccf-mtDNA in the plasma of individuals infected with HCV. HCVi showed a significantly higher ccfmtDNA copy number than SVR. Mitochondrial DNA (mtDNA) is a circular, maternally inherited, double-stranded extrachromosomal DNA that measures approximately 16.5 kb [49]. Previous studies have shown significantly lower mitochondrial DNA copy numbers in the livers of HCV-infected patients than in those of healthy individuals [50]. Conversely, ccfmtDNA levels were higher in patients with HCV than in healthy controls [20]. Our findings showed elevated ccfmtDNA levels in plasma during HCV infection compared with those after SVR. This suggests an improvement in the hepatic mitochondrial health following viral clearance. Interestingly, in our study, non-cirrhotic patients exhibited significantly higher ccfmtDNA levels during HCV infection than did cirrhotic patients. Similarly, when evaluating ccfmtDNA in hepatitis B, Li et al. reported the highest ccfmtDNA copy numbers in non-cirrhotic HBV patients compared to their cirrhotic counterparts and HCC patients [19]. We observed a significant reduction in ccfmtDNA copy numbers in SVR patients compared to HCVi non-cirrhotic patients (Figure 4A), suggesting mitochondrial recovery in non-cirrhotic patients after antiviral therapy. Normally, cellular mitochondria are contained within the mitochondrial matrix, limiting their release [51]. While the exact origins of ccfmtDNA in cell-free samples (serum or plasma) remain unclear, they are thought to be derived from necrotic and apoptotic cells in the bloodstream or target tissues [19] in this case hepatocytes, similar to reports on HBV [52]. Based on our ccfmtDNA findings, we propose two mechanisms: (1) viral clearance enhances overall improvement in mitochondria, and non-cirrhotic patients show the greatest initial improvement; and (2) cirrhosis itself is associated with mitochondrial depletion in the liver extending beyond viral infection. Further research is required to elucidate the mechanisms underlying this hypothesis.

These findings raise the possibility that the diagnostic and monitoring value of ccfmtDNA is largely confined to non-cirrhotic HCV patients, in whom this marker tracked significantly with viral clearance. In cirrhotic patients, the absence of a significant change in ccfmtDNA despite viral cure suggests that hepatic mitochondria in fibrotic/cirrhotic livers may already have reached a state of chronic depletion or dysfunction that is not readily reversed by antiviral therapy alone, limiting the discriminatory value of ccfmtDNA once cirrhosis is established. Future studies with larger cirrhotic cohorts and longer follow-up will be needed to determine whether ccfmtDNA can detect more gradual mitochondrial recovery in this population, or whether alternative circulating biomarkers are needed for monitoring cirrhotic patients.

Notably, the intrinsic mitochondrial transcription machinery (TFAM, TFB1M, TFB2M, POLRMT) and the peroxisome proliferation factors (PEX11A/B/G) were unchanged between HCVi and SVR (Supplementary Table S2), indicating that the downregulation of downstream metabolic effectors occurs without a corresponding change in the organelle’s transcriptional apparatus. This dissociation argues that HCV-associated mitochondrial and peroxisomal dysfunction is driven predominantly at the functional and post-transcriptional level consistent with our protein level (ATP5A, PMP70, catalase) and ccfmtDNA findings while the canonical upstream regulators (PGC-1α, NRF1, ERRα, PPAR/RXR), which lie outside the organellar gene inventories analyzed here, remain an important target for future mechanistic study.

Despite these promising results, this study had several limitations. This study was conducted in a single cohort from one clinical site (NIH); given the paired, pre- and post-antiviral design of our dataset which differs structurally from the case–control design of most public HCV liver transcriptomics datasets, future studies incorporating multi-cohort meta-analysis will be valuable in confirming the generalizability of these findings. A direct transcriptomic comparison between cirrhotic and non-cirrhotic patients during active HCV infection was previously reported in this cohort as part of our multi-omics analysis of the gut–liver axis [2]; a dedicated adequately powered comparison restricted to the mitochondrial and peroxisomal gene set described here remains a goal for future work with a larger cohort. Our post SVR biopsies were obtained at a single time point (6 months), and we cannot distinguish partial, ongoing organelle recovery from incomplete response; longitudinal sampling beyond 6 months would be needed to determine the full trajectory or durability of organelle recovery. Whole-liver transcriptomics may mask cell-specific gene expression patterns because of the averaging across all cell types. Consequently, subtle changes in gene expression that are specific to particular cell populations may not be revealed. Although single-cell or single-nucleus RNA sequencing would help resolve this issue, such data were not generated in this cohort, and no publicly available dataset with our matched clinical design currently exists for comparison. Therefore, generating matched single-cell/nucleus dataset in a future cohort is an important next step. Immunofluorescence identifies the presence of ATP5A/PMP70/catalase expression in a given location; however, there is a gap in direct information on the functional status of the protein (e.g., phosphorylation state, protein–protein interactions, or cellular activity), which may be important in understanding disease mechanisms. Moreover, due to tissue unavailability no zonation markers were added in the immunofluorescence study. While the current study focused on HCV, the elevation of ccf-mtDNA is not specific to this infection and reflects global mitochondrial stress; it can be observed in other pathological conditions, such as other viral infections, autoimmune diseases, and metabolic disorders. Therefore, establishing specific ccf-mtDNA patterns in combination with other biomarkers is essential to understand the pathogenesis and improve the specificity of liver disease progression.

## 5. Conclusions

Our transcriptomic analysis revealed dysregulation of mitochondrial and peroxisomal genes in chronic HCV infection and cirrhosis, highlighting the vulnerability of the hepatic periportal zone to damage. Future research should elucidate the molecular mechanisms underlying this susceptibility and explore interventions to enhance organelle resilience. Additionally, elevated ccf-mtDNA levels in HCV-infected individuals suggest their potential as a disease progression tool and a pro-biomarker. Further studies are needed to explore the mechanisms of cf-mtDNA release and to assess its clinical utility as a biomarker for liver diseases. The central role of peroxisomes and mitochondria in HCV-related liver damage offers insight into the metabolic alterations and therapeutic pathways. Further research using larger and more diverse cohorts is crucial to validate these findings.

## Figures and Tables

**Table 1:** Clinical and demographic characteristics of the study cohort and sample distribution across experimental analysis.

	HCVi	SVR	Wilcoxon Paired Test (HCVi vs. SVR)
Number of subjects, *n*	29	23	22
Non-cirrhotic, *n*	16	14	-
Cirrhotic, *n*	13	9	-
Age; median (IQR)	59 (54.5–62)	58 (48–67)	-
Gender; males, *n* (%)	18 (62.1)	14 (61)	-
Race/ethnicity, *n* (%)			
Caucasian	19 (65.5)	15 (65.2)	-
African American	5 (17.2)	5 (21.7)	
Asian	2 (6.8)	1 (4.3)	-
Hispanic	3 (10.3)	2 (8.7)	-
HCV genotype; *n* (%)			
1a	10 (34.5)	-	-
1b	8 (27.5)	-	-
2a/c	1 (3.4)	-	-
2b	5 (17.2)	-	-
3a	4 (13.8)	-	-
4	1 (3.4)	-	-
Histological and laboratory parameters, median (IQR)			
Log HCV RNA (IU/mL)	6.4 (5.75–6.88)	Undetectable	-
Ishak fibrosis score	4 (2–6)	3 (0–6)	0.2483
HAI inflammatory score	8 (7–10)	3 (2–3)	<0.0001
Laboratory parameters, median (IQR)			
ALT (IU/L)	85 (46.0–145.5)	21 (17–29)	<0.0001
AST (IU/L)	72 (34.5–109.5)	24 (21–28)	<0.0001
ALP (IU/L)	81 (73.0–108.5)	76 (63–106)	0.1172
BMI (kg/m^2^)	27.3 (24.4–31.4)	27.7 (23.8–31.1)	0.2313
Samples included in individual analysis, *n*			
Transcriptomic analysis			22
Non-cirrhotic	14	14	-
Cirrhotic	8	8	-
Immunofluorescence analysis			
Non-cirrhotic	13	11	-
Cirrhotic	10	5	-
ccf-mtDNA analysis			
Non-cirrhotic	16	14	-
Cirrhotic	13	8	-

Note: Data are presented as *n* (%) or median (interquartile range (IQR)). Patients with Ishak fibrosis scores of 0–4 were classified as non-cirrhotic, whereas those with scores 5–6 were classified as cirrhotic. The initial HCVi cohort included 29 patients, of whom 23 returned for evaluation 6 months after SVR. Sample numbers varied among experimental analysis because of tissue availability and quality control requirements. Transcriptomic analysis included 22 paired HCVi-SVR samples which included HCVi non-cirrhotic *n* = 14, HCVi cirrhotic *n* = 8, SVR non-cirrhotic *n* = 14, and SVR cirrhotic *n* = 8. Immunofluorescence analysis included HCVi non-cirrhotic *n* = 13, HCVi cirrhotic *n* = 10, SVR non-cirrhotic *n* = 11, and SVR cirrhotic *n* = 5. ccfmtDNA analysis included HCVi non-cirrhotic *n* = 16, HCVi cirrhotic *n* = 13, SVR non-cirrhotic *n* = 14, and SVR cirrhotic *n* = 8. For paired analysis Wilcoxon matched-pairs signed rank test was performed. Abbreviations: ALP, alkaline phosphatase; ALT, alanine aminotransferase; AST, aspartate aminotransferase; BMI, body mass index; HAI, hepatic activity index; HCVi, patients infected with HCV; SVR, sustained virologic response.

**Table 2:** List of primers.

Gene Names	Forward Sequence	Reverse Sequence
MT-ND1	5′-GTATTCCCCCAGGTTTACATGTTC-3′	5′-ACTCACTCCTGGAAGATGGTGAT-3′
MT-ND6	5′-CCAATCCTACCTCCATCGCT-3′	5′-GAGTATCCTGAGGCATGGGG-3′
MT-COXIII	5′-ATGACCCACCAATCACATGC-3′	5′-ATCACATGGCTAGGCCGGAG-3′
b-actin	5′-CACCATTGGCAATGAGCGGTTC-3′	5′-AGGTCTTTGCGGATGTCCACGT-3′

## Data Availability

The original contributions presented in this study are included in the article/Supplementary Material. Further inquiries can be directed to the corresponding author.

## Supplementary Materials

The following supporting information can be downloaded at https://www.mdpi.com/article/10.3390/cimb48090877/s1.

## Abbreviations

ACADL	Acyl-CoA Dehydrogenase Long Chain
ACOX1	Acyl-CoA Oxidase 1
ACSM3	Acyl-CoA Synthetase Medium-chain family Member 3
ATP5A	ATPase 5A
ALT	Alanine Aminotransferase
ALP	Alkaline Phosphatase
AST	Aspartate Aminotransferase
BSA	Bovine Serum Albumin
BMI	Body Mass Index
CBR4	Carbonyl Reductase 4
CAT	Catalase
Ccf-mtDNA	Circulating Cell-Free Mitochondrial DNA
cfDNA	Cell-free DNA
DAA	Direct-Acting Antiviral
DAMP	Damage-Associated Molecular Pattern
DEGs	Differentially Expressed Genes
FDR	False Discovery Rate
HAI	Hepatic Activity Index
HCV	Hepatitis C virus
HCVi	Hepatitis C infected
H_2_O_2_	Hydrogen Peroxide
IL-1β	Interleukin 1 beta
KEGG	Kyoto Encyclopedia of Genes and Genomes
LRPPRC	Leucine Rich Pentatricopeptide Repeat Containing
mtDNA	Mitochondrial DNA
MT-ND1	Mitochondrially Encoded NADH: Ubiquinone oxidoreductase core subunit 1
MT-ND6	Mitochondrially encoded NADH dehydrogenase subunit 6
MT-COXIII	Mitochondrially encoded Cytochrome C Oxidase III
MTERF3	Mitochondrial Transcription Termination Factor 3
MTERF4	Mitochondrial Transcription Termination Factor 4
PEX 11A	Peroxisomal Biogenesis Factor 11 Alpha
PEX 11B	Peroxisomal Biogenesis Factor 11 Beta
PEX 11G	Peroxisomal Biogenesis Factor 11 Gamma
PHYH	Phytanoyl-CoA 2-Hydroxylase
PMP70	Peroxisomal Membrane Protein 70
POLRMT	RNA Polymerase Mitochondrial
PPAR	Peroxisome Proliferator-Activated Receptor
ROS	Reactive Oxygen Species
SLIRP	SRA Stem-loop Interacting RNA Binding Protein
SIRT3	Sirtuin3
SVR	Sustained Virologic Response
TGFβ	Transforming Growth Factor beta
TLR9	Toll-Like Receptor 9
TNFα	Tumor Necrosis Factor alpha
TEFM	Transcription Elongation Factor, Mitochondrial
TFAM	Transcription Factor A, Mitochondrial
TFB1M	Transcription Factor B1, Mitochondrial
