# Supplementary material for: Transcriptomic and Zonal Signatures of Mitochondrial Peroxisomal Dysfunction in HCV Associates with Circulating Mitochondrial DNA Biomarkers
Source: Curr Issues Mol Biol. 2026 Aug 29;48(9):877. doi: 10.3390/cimb48090877 (PMC13605421; doi:10.3390/cimb48090877)
Supplement: Supplementary file 1 [file cimb-48-00877-s001.zip › cimb-4481151-supplementary.pdf]

Supplementary Table S1: A complete list of differentially expressed genes (DEGs) specific to mitochondria and peroxisomes for each group, including associated statistical values.

Gene	Organelle	FoldChange	PValue	Adjusted PValue	Cirr_Fold Change	Cirr_P Value	Cirr_AdjustedPValue	NC_Fold Change	NC_P Value	NC_AdjustedPValue	HCVi_Fold Change	HCVi_P value	HCVi_AdjustedPValue	SVR_Fold Change	SVR_P value	SVR_AdjustedPValue
AADAT	Mitochondria	-0.6961926	6.973E-05	0.0015651	-0.8798368	0.003806	0.0913217	-0.6089457	0.0136725	0.1409975	-0.4665502	0.0617605	0.9998749	-0.0017495	0.9931457	
AARS2	Mitochondria	0.3387484	0.2331479		0.2850085	0.5604701		0.3706564	0.3562115		0.1537328	0.6307112	0.9998749	0.3686574	0.3196356	
AASS	Mitochondria	-0.4146011	0.0085257	0.0698189	-0.4524774	0.0447569	0.4267521	-0.4028205	0.0366202	0.2746512	-0.5603675	0.0858842	0.9998749	-0.5795826	9.909E-05	0.0060905
ABAT	Mitochondria	-0.7082537	5.633E-10	5.689E-08	-0.7308557	1.339E-05	0.001372	-0.7083653	8.087E-06	0.0006672	-0.2117992	0.3833839	0.9998749	0.0566808	0.6561264	0.9365555
ABCA9	Mitochondria	-0.1163203	0.4233263	0.7479033	0.065587	0.7991563	0.9854749	-0.2367406	0.2075312	0.5988145	-0.0086688	0.9727256	0.9998749	-0.1023036	0.6148824	0.9365555
ABCB10	Mitochondria	-0.1285748	0.5015923	0.803711	0.012334	0.9725413		-0.2304027	0.3879263		-0.0116867	0.9592738	0.9998749	-0.1554833	0.5152634	
ABCB6	Mitochondria	0.3171861	0.2558089		0.0932127	0.8619532		0.4319129	0.2410082		-0.3659862	0.2677903	0.9998749	-0.0429278	0.9083179	
ABCB7	Mitochondria	0.0208447	0.9333023		0.0948776	0.8438415		-0.0297191	0.9283482		0.0029149	0.99207	0.9998749	-0.2779273	0.3845625	
ABCB8	Mitochondria	0.4614888	0.4016162		-0.0147065	0.9877999		0.7594272	0.3196091		0.0356325	0.9514072	0.9998749	0.5285831	0.4773879	
ABCD1	Peroxisome	0.699642	0.0626378		0.4663019	0.4589499		0.8762493	0.1075265		0.0864393	0.825985	0.9998749	0.6762528	0.1928522	
ABCD3	Peroxisome	-0.0829468	0.3085866	0.6900496	0.0141303	0.9315187	0.9909626	-0.165737	0.1824738	0.5645282	0.0247286	0.8714847	0.9998749	0.0350491	0.7579252	0.9526674
ABHD10	Mitochondria	-0.3576505	0.045138	0.2298624	-0.1755477	0.5847717		-0.5105468	0.0470509	0.3024698	0.1968848	0.3817047	0.9998749	-0.0426884	0.8398269	
ABHD11	Mitochondria	0.4289827	0.4259982		0.2514525	0.8004626		0.5236007	0.463738		0.1546251	0.7924571	0.9998749	0.2327509	0.7440965	
ACAA1	Peroxisome	-0.1938769	0.0477134	0.2383612	-0.3962645	0.026413	0.3451674	-0.0886297	0.4834123	0.8111495	-0.2159573	0.1076796	0.9998749	-0.02048	0.9131302	0.9660934
ACAA2	Mitochondria	-0.2355684	0.0433148	0.2243483	-0.3741758	0.0516316	0.4451681	-0.1685644	0.2440843	0.6298	-0.1321357	0.420333	0.9998749	0.0035816	0.9840022	0.9915424

ACACA	Mitochondria	-0.1610373	0.4414012	0.7513739	-0.2283976	0.5210169		-0.1299837	0.6708648		0.260211	0.3327923	0.9998749	0.4548725	0.0889137	
ACACB	Mitochondria	-0.3533445	0.0009751	0.0140694	-0.5124507	0.0060912	0.1206858	-0.2729345	0.068875	0.3581046	-0.2786315	0.15482	0.9998749	0.0003053	0.9985063	0.9985063
ACAD10	Mitochondria	-0.0074679	0.9720197	0.988921	-0.0701926	0.8469583		0.0236879	0.9352722	0.9778656	-0.0935089	0.7325104	0.9998749	-0.0610848	0.8032333	
ACAD11	Mitochondria	-0.2387725	0.0536981	0.2542271	-0.3017695	0.188656	0.7813025	-0.2151758	0.2190762	0.6138361	-0.3679756	0.0639393	0.9998749	-0.2250964	0.178477	0.5274096
ACAD8	Mitochondria	-0.0867512	0.7112391		-0.1880211	0.6563689		-0.0359899	0.9128745		-0.0272052	0.9216971	0.9998749	0.2502371	0.3828766	
ACAD9	Mitochondria	0.1182208	0.4758217	0.7878359	0.0580145	0.8497559	0.9854749	0.1515053	0.5238997	0.831187	0.1295247	0.4920576	0.9998749	0.1992875	0.3593035	
ACADL	Mitochondria	-0.5689616	0.0003309	0.0060757	-0.4621942	0.1313741	0.6874549	-0.6421137	0.0032127	0.0513004	-0.2472174	0.3001608	0.9998749	-0.2741659	0.1547289	
ACADM	Mitochondria	-0.419106	0.0016407	0.0220943	-0.2080998	0.2917279	0.9358893	-0.5689118	0.0028859	0.0476176	-0.003797	0.985921	0.9998749	-0.1528458	0.2734809	0.6659767
ACADS	Mitochondria	-0.1352278	0.4812348	0.7903206	-0.4469187	0.1992141		0.0625074	0.8127496	0.9493368	-0.4018021	0.0951847	0.9998749	0.1881406	0.5498846	
ACADSB	Mitochondria	-0.6676822	3.62E-12	7.312E-10	-0.5602271	0.0003583	0.014689	-0.7426184	6.155E-08	1.523E-05	-0.4320189	0.0293808	0.9998749	-0.5298651	0.0001422	0.0060905
ACADVL	Mitochondria	0.1858723	0.0503513	0.2460718	0.0727953	0.6745554	0.9854749	0.2545928	0.0783533	0.3729317	-0.0572694	0.6450231	0.9998749	0.1466665	0.3632615	0.770466
ACAT1	Mitochondria	-0.6012387	1.819E-09	1.378E-07	-0.5952598	0.0001222	0.0073418	-0.6214657	1.418E-05	0.0008856	-0.1402595	0.405512	0.9998749	-0.0195351	0.8883837	0.9660934
ACCS	Mitochondria	0.3684728	0.4490779		0.3372341	0.7087762		0.3954377	0.542534		-0.3080037	0.5965597	0.9998749	0.0782941	0.9029926	
ACLY	Mitochondria	0.3983865	0.2044571		0.471115	0.3856796		0.3199615	0.4719522		0.3592175	0.2919004	0.9998749	0.3297171	0.4270253	
ACO2	Mitochondria	0.3027258	0.0355607	0.1995351	0.1879469	0.4751157	0.9698776	0.3561665	0.0872458	0.4078875	0.1609875	0.4027667	0.9998749	0.3868774	0.0356973	
ACOT13	Mitochondria	-0.2393453	0.0041716	0.0407742	-0.2286399	0.1812083	0.7739103	-0.2535022	0.0356539	0.2733544	-0.1252129	0.4141153	0.9998749	-0.4303984	8.246E-06	0.0021686
ACOT2	Mitochondria	-0.0732504	0.6759824	0.9184946	-0.3261403	0.3305494		0.0587557	0.8048793	0.9493368	-0.3779685	0.1535712	0.9998749	0.0437586	0.8747739	
ACOT7	Mitochondria	0.4730313	0.2054403		0.3242189	0.6045551		0.5769708	0.281462		0.3715929	0.353784	0.9998749	0.6581967	0.1831344	

ACOT9	Mitochondria	0.4882028	0.1081935		0.4926616	0.3637256		0.4776576	0.2493625		0.2113904	0.51541	0.9998749	0.0881895	0.830532	
ACP6	Mitochondria	-0.1618619	0.5900319		-0.1714806	0.7492072		-0.160626	0.7030904		0.1316048	0.7123259	0.9998749	0.1089075	0.7709731	
ACSF2	Mitochondria	0.3040896	0.1472771	0.4747337	0.1527238	0.6879295		0.3926955	0.1764384		0.0155525	0.9489966	0.9998749	0.3285573	0.2221073	
ACSF3	Mitochondria	-0.1233625	0.7835085		-0.0754719	0.9229753		-0.1650205	0.7931076		0.3284555	0.5325454	0.9998749	0.2495623	0.6536385	
ACSL1	Peroxisome	-0.3402996	0.0050695	0.0470952	-0.4251192	0.0129069	0.2300802	-0.3022971	0.0674563	0.356451	-0.1520499	0.5045706	0.9998749	0.2889046	0.031709	0.2527115
ACSL6	Peroxisome	-0.2531132	0.6283463		0.1342026	0.8830627		-0.5004672	0.4878272		0.3217841	0.6073578	0.9998749	-0.0592748	0.9270349	
ACSM1	Mitochondria	0.9151079	0.012109		1.4367159	0.0110643		0.4691854	0.3822665		0.0488284	0.9354199	0.9998749	0.3689084	0.5662182	
ACSM2A	Mitochondria	-0.041043	0.8673613	0.9644421	-0.351174	0.3171578	0.9358893	0.1364114	0.6793043	0.9036861	-0.446236	0.3820823	0.9998749	-0.2226822	0.3020991	0.7028402
ACSM2B	Mitochondria	-0.0481036	0.7942066	0.9456563	-0.3637594	0.0980931	0.6002714	0.1326073	0.5642349	0.8510009	-0.3147929	0.4183364	0.9998749	0.0348526	0.8622034	0.9660934
ACSM3	Mitochondria	-0.9909865	3.944E-10	4.78E-08	-0.9433891	0.000165	0.0075151	-1.0324137	1.821E-06	0.0002577	-0.4559776	0.0604276	0.9998749	-0.4871483	0.0035143	0.0577657
ACSM5	Mitochondria	-0.137576	0.1678112	0.499869	-0.204619	0.308128	0.9358893	-0.1153442	0.3675058	0.7247624	-0.2891167	0.1528978	0.9998749	-0.3102147	0.0256949	0.2179918
ACSS1	Mitochondria	0.7171624	0.1418067		0.4426269	0.5956954		0.8953422	0.1872718		0.3098956	0.5316189	0.9998749	0.6449971	0.3401523	
ACSS3	Mitochondria	-0.3380235	0.036247	0.1996881	-0.2103429	0.4261184	0.9506771	-0.4551339	0.0458619	0.2987058	0.535134	0.0473481	0.9998749	0.5286277	0.0045417	0.0663589
ADCK1	Mitochondria	0.9436233	0.0368682		0.8375962	0.3157971		0.9825687	0.0962879		-0.1060329	0.8192367	0.9998749	-0.0611282	0.9247706	
ADCK2	Mitochondria	0.2352347	0.5018621		0.2664805	0.6728035		0.2070175	0.665805		0.1871448	0.6269087	0.9998749	0.1094433	0.8113899	
ADCK5	Mitochondria	0.1212754	0.8403869		0.0981892	0.9232908		0.1472691	0.8636526		0.0968551	0.8898585	0.9998749	0.3518264	0.6473406	
ADCY10	Mitochondria	-0.263631	0.57265		0.0543774	0.9518088		-0.4339564	0.4747271		-0.3033779	0.5993643	0.9998749	-0.6138565	0.3047469	
ADHFE1	Mitochondria	-0.1212713	0.3320174	0.713693	-0.1480341	0.5531949	0.9854749	-0.1124108	0.5167631	0.8259241	-0.4209344	0.0394634	0.9998749	-0.2104518	0.2057887	0.5697097

AFG1L	Mitochondria	0.1662969	0.6969879		0.681634	0.3826817		-0.1571757	0.7859188		0.2268791	0.6346307	0.9998749	-0.3475074	0.5377213	
AFG3L2	Mitochondria	0.0970336	0.3860032	0.731203	0.07121	0.7483964	0.9854749	0.0920629	0.5712922	0.8510009	0.0610809	0.6577413	0.9998749	0.0541839	0.7015249	0.9386991
AGK	Mitochondria	0.0529953	0.7672329	0.9456563	0.2485531	0.4467539		-0.0902879	0.7198164	0.9159826	0.0555984	0.7990064	0.9998749	-0.0050349	0.9819977	
AGMAT	Mitochondria	-0.0356897	0.7257207	0.9456563	-0.0868562	0.6240003	0.9854749	-0.0127238	0.9312099	0.9778656	0.0126061	0.9468714	0.9998749	0.0659046	0.6425461	0.9365555
AGPAT4	Mitochondria	-0.0110969	0.9849362		0.6000768	0.5201457		-0.4786486	0.5724505		1.1298184	0.0800655	0.9998749	0.2311845	0.7490649	
AGPAT5	Mitochondria	0.0410365	0.8303942	0.9514354	0.4352482	0.2429057		-0.1800107	0.4765655	0.8051192	-0.1527851	0.5251734	0.9998749	-0.7813651	0.0055302	
AGXT	Peroxisome	0.0291625	0.8241058	0.9476434	-0.3655679	0.0367867	0.3770636	0.246769	0.1268965	0.4889271	-0.3326657	0.0452134	0.9998749	0.1694321	0.500028	0.8767158
AGXT2	Mitochondria	-0.3067795	0.0155243	0.103659	-0.3865265	0.1201085	0.6565929	-0.271091	0.1028775	0.4475676	-0.4700112	0.010711	0.9998749	-0.1471531	0.3511984	0.7567204
AHCYL1	Mitochondria	0.2646438	0.0340609	0.1947257	0.2675916	0.2151964	0.8236914	0.2541736	0.1335315	0.5007432	-0.0262372	0.8862531	0.9998749	-0.1580085	0.2648005	0.6629064
AIFM1	Mitochondria	-0.0827255	0.4563443	0.7660516	-0.1755535	0.4072491	0.9464092	-0.0517867	0.7466606	0.922111	0.0447916	0.7564281	0.9998749	0.1739637	0.1664305	0.5205244
AIFM2	Mitochondria	0.5740255	0.0863444		0.3859011	0.5002197		0.6892533	0.1472319		0.2224754	0.5238445	0.9998749	0.5255098	0.2442861	
AK2	Mitochondria	0.1536643	0.3321145	0.713693	0.2294362	0.4450438	0.9553296	0.0870972	0.69585	0.9088278	0.0139334	0.9407306	0.9998749	-0.0195501	0.923153	
AK3	Mitochondria	-0.0141165	0.9159196	0.9715772	0.2134423	0.3492465	0.9358893	-0.1669517	0.3690106	0.7248422	0.1878158	0.3836162	0.9998749	-0.0534619	0.7629117	0.9526674
AK4	Mitochondria	-0.233723	0.0118519	0.0855031	-0.2347764	0.2204555	0.8236914	-0.2494953	0.0676897	0.356451	-0.1430683	0.2267596	0.9998749	-0.0956156	0.445421	0.8367551
AKAP1	Mitochondria	-0.0089373	0.9412376	0.981362	-0.1679256	0.4646183	0.96694	0.0851663	0.6274357	0.8785326	0.021282	0.8868961	0.9998749	0.2513247	0.0869677	0.4315568
AKAP10	Mitochondria	0.2100625	0.402662		0.563719	0.1967973		-0.0439631	0.9008101		0.3337153	0.2338541	0.9998749	0.0737356	0.8209376	
AKR1B10	Mitochondria	2.3575565	0.0026319		2.484538	4.13E-05		2.0458335	0.4892164		0.78862	0.4246655	0.9998749	3.29836	0.0006534	
AKR7A2	Mitochondria	-0.1388033	0.4998859	0.803711	-0.2348078	0.5305541		-0.0905407	0.7552189		-0.0151992	0.949911	0.9998749	0.1727624	0.5030906	

ALAS1	Mitochondria	-0.1115075	0.4330058	0.7499764	-0.419692	0.1025236	0.6091981	0.0633866	0.7478589	0.922111	-0.2741123	0.2052789	0.9998749	0.2544167	0.1555693	0.5114342
ALDH18A1	Mitochondria	-0.1626288	0.633969		0.1159058	0.8559734		-0.3393003	0.4637453		-0.0438348	0.9117994	0.9998749	-0.3011825	0.48383	
ALDH1B1	Mitochondria	-0.1042527	0.2609409	0.6376217	-0.2882737	0.1060093	0.6189302	-0.000918	0.994147	0.9946164	-0.048907	0.7805201	0.9998749	0.2969677	0.1206715	0.4871108
ALDH1L1	Mitochondria	-0.4517505	0.0003975	0.0070851	-0.6203069	0.0029782	0.0763176	-0.3536846	0.0530832	0.3204414	-0.3719753	0.1845859	0.9998749	-0.0981606	0.7102704	0.9386991
ALDH1L2	Mitochondria	0.764971	0.5815514		1.1173624	0.3921786					0.9581323	0.2338604	0.9998749			
ALDH2	Mitochondria	-0.6128188	7.089E-12	1.074E-09	-0.6134482	5.861E-06	0.0012016	-0.627351	3.153E-06	0.0003122	-0.1120305	0.491358	0.9998749	-0.2051628	0.2017415	0.5644469
ALDH3A2	Mitochondria	-0.2451834	0.0109681	0.0810571	-0.1319457	0.4290492	0.9506771	-0.3373402	0.0168797	0.1606814	0.1156537	0.5153361	0.9998749	0.0647225	0.4918397	0.8740124
ALDH4A1	Mitochondria	-0.0333088	0.8083128	0.9456563	-0.2817433	0.1955045	0.7865408	0.1408057	0.490707	0.8181458	0.1953191	0.2466263	0.9998749	0.6740722	0.0001853	0.0060905
ALDH5A1	Mitochondria	-0.4346439	2.775E-05	0.0007645	-0.3650753	0.0464005	0.4275315	-0.5102539	0.0016324	0.0288576	0.0522048	0.7859681	0.9998749	0.0715206	0.5422882	0.8951228
ALDH6A1	Mitochondria	-0.3724934	0.0009202	0.0136014	-0.3182977	0.088084	0.5920397	-0.4145237	0.0066362	0.0912484	-0.2109539	0.3193103	0.9998749	-0.2163653	0.0835046	0.4243582
ALDH7A1	Mitochondria	-0.4917029	7.797E-07	2.953E-05	-0.4568728	0.0238725	0.3451674	-0.5226481	0.0003807	0.0082392	-0.1708938	0.2188759	0.9998749	-0.1891379	0.1020734	0.440087
ALDH9A1	Mitochondria	-0.270768	0.002816	0.0310273	-0.2262959	0.2046316	0.8145528	-0.3224212	0.0216555	0.1948992	-0.0811199	0.5830966	0.9998749	-0.0491185	0.6512162	0.9365555
ALKBH1	Mitochondria	0.0174951	0.9638477		0.0705739	0.9201615		-0.0314105	0.9524774		-0.0140539	0.9749615	0.9998749	-0.0299797	0.9516355	
ALKBH7	Mitochondria	-0.0565686	0.843772		-0.499021	0.3459822		0.200439	0.6095513		-0.245878	0.4526568	0.9998749	0.137045	0.7079931	
AMACR	Peroxisome	-0.3379779	0.0019311	0.0238822	-0.1834084	0.3362468	0.9358893	-0.4402199	0.0034671	0.0531655	0.0616433	0.7319555	0.9998749	-0.0173667	0.9275755	0.9660934
AMT	Mitochondria	-0.2450624	0.3078032		-0.2517329	0.5634531		-0.2433281	0.464797		-0.0669755	0.8189139	0.9998749	0.0391473	0.8918887	
ANGEL2	Mitochondria	-0.1916811	0.4326526		0.019525	0.9636276		-0.3577614	0.2985443		0.3033911	0.2933994	0.9998749	0.0213397	0.9424747	

APEX1	Mitochondria	0.1255101	0.3749363	0.731203	0.1194871	0.593126	0.9854749	0.1274629	0.5387059	0.8443472	0.1155641	0.4675274	0.9998749	0.1393993	0.510198	0.8827768
APOO	Mitochondria	0.3254738	0.3656493		0.219244	0.7434486		0.3719797	0.4419979		-0.0406395	0.9158067	0.9998749	-0.0497216	0.9176882	
APOOL	Mitochondria	-0.2319797	0.1459741	0.4747337	-0.1996836	0.4693196	0.96694	-0.2801581	0.234659	0.6211561	0.2196999	0.3205952	0.9998749	0.2894053	0.1335705	
ARF5	Mitochondria	0.1559485	0.5044851		-0.0459539	0.9129441		0.2813329	0.3909481		0.0414822	0.8761291	0.9998749	0.3539909	0.233292	
ARG2	Mitochondria	-0.4027768	0.3789662		-0.2063016	0.7959645		-0.6066818	0.3481945		0.2305679	0.6756858	0.9998749	-0.0595048	0.9132589	
ARL2	Mitochondria	0.4481663	0.4946021		0.7143941	0.5400424		0.2987916	0.7350561		0.6666134	0.2989137	0.9998749	-0.164032	0.8592935	
ARMC10	Mitochondria	-0.191901	0.4693736		0.1057983	0.8265753		-0.3952485	0.2791217		0.1718003	0.5791561	0.9998749	-0.2080721	0.5232432	
ARMCX1	Mitochondria	0.4065777	0.3424341		0.6967081	0.3214539		0.1920018	0.74876		0.752462	0.0912889	0.9998749	0.5097782	0.3775084	
ARMCX2	Mitochondria	0.5945027	0.4653264		0.9509647	0.4524082		0.3920468	0.7178891		0.4797229	0.5083392	0.9998749	-0.0799711	0.9393935	
ARMCX3	Mitochondria	0.165705	0.4245524	0.7479033	0.4074952	0.2869254		0.0268171	0.9214202	0.9778656	0.1136231	0.6281703	0.9998749	-0.313312	0.3096698	
ARMCX6	Mitochondria	0.6797668	0.1321337		0.1304747	0.8693416		1.0266114	0.0990138		-0.000881	0.9984828	0.9998749	0.5190672	0.4069683	
ATAD1	Mitochondria	0.2047817	0.3278291	0.713693	0.1138184	0.7675834		0.2485958	0.3868351		-0.0131237	0.9558238	0.9998749	-0.0129189	0.9617424	
ATAD3A	Mitochondria	0.3250756	0.378689		0.3005842	0.6546437		0.3192661	0.5286769		0.0346016	0.9348023	0.9998749	0.1260697	0.7965092	
ATAD3B	Mitochondria	0.6517253	0.1712773		0.2890336	0.7274182		0.8970826	0.1728544		-0.0930755	0.8516572	0.9998749	0.6336726	0.3232445	
ATP23	Mitochondria	0.011288	0.9819005		0.3030683	0.7475984		-0.1478234	0.823701		-0.3571512	0.5561862	0.9998749	-0.3711354	0.5696781	
ATP5F1A	Mitochondria	-0.070913	0.4850221	0.7937656	-0.1820702	0.3344677	0.9358893	-0.0300154	0.8465439	0.9545313	0.159328	0.2927193	0.9998749	0.3355427	0.0062783	0.0869051
ATP5F1B	Mitochondria	0.1730742	0.1136664	0.4071278	0.1723644	0.3775206	0.9464092	0.1516048	0.3026869	0.6872937	0.2487972	0.1986686	0.9998749	0.266727	0.0277013	0.2276704
ATP5F1C	Mitochondria	0.1289659	0.3051352	0.6869339	0.0153346	0.9496664	0.9958138	0.1711577	0.3378429	0.7027967	-0.0733277	0.6689804	0.9998749	-0.0532402	0.7285994	0.9482562
ATP5F1D	Mitochondria	0.2994616	0.2381703		-0.2313137	0.6161257		0.598023	0.0764768		-0.311055	0.2829536	0.9998749	0.2415388	0.4559569	
ATP5F1E	Mitochondria	0.4275405	0.0019818	0.0240194	0.5444691	0.0008367	0.0285859	0.3482238	0.0672518	0.356451	0.1713457	0.448571	0.9998749	-0.4695693	0.0364334	0.2704669

ATP5IF1	Mitochondria	0.0377458	0.7714795	0.9456563	-0.0310627	0.8938487	0.9854749	0.0649934	0.7223839	0.9159826	0.1710409	0.4180911	0.9998749	-0.0787021	0.6749723	0.9365555
ATP5MC1	Mitochondria	0.142999	0.6450261		-0.1980715	0.7314172		0.329521	0.4297376		-0.0162529	0.9620076	0.9998749	0.3069866	0.4330345	
ATP5MC2	Mitochondria	0.1718978	0.1504256	0.4823169	-0.027559	0.901793	0.9872524	0.289047	0.0891269	0.4084982	0.1632525	0.2779651	0.9998749	0.2251385	0.1420424	0.4871108
ATP5MC3	Mitochondria	-0.0705882	0.6346317	0.8971453	-0.257769	0.3577777	0.9464092	0.0276372	0.8942982	0.9722587	-0.0135746	0.9400338	0.9998749	0.2047197	0.2549125	
ATP5MD	Mitochondria	0.0790538	0.5385887	0.8297981	0.043754	0.8532729	0.9854749	0.0724469	0.6839682	0.9036861	-0.089824	0.6931532	0.9998749	-0.3251483	0.083725	0.4243582
ATP5ME	Mitochondria	0.0482918	0.7367219	0.9456563	-0.2670948	0.2655054	0.931818	0.2174412	0.2438768	0.6298	0.1931961	0.4754586	0.9998749	0.0304207	0.8669655	0.9660934
ATP5MF	Mitochondria	0.2710723	0.0575378	0.2641506	0.05938	0.8288842	0.9854749	0.3783641	0.0496864	0.3083484	-0.008046	0.967544	0.9998749	0.0898304	0.6170663	
ATP5MF-PTCD1	Mitochondria	0.3215273	0.3973119		-0.1198198	0.8572966		0.6184847	0.2397509		-0.0432201	0.9159408	0.9998749	0.5237439	0.2887567	
ATP5MG	Mitochondria	0.0395114	0.7087153	0.9355783	0.0636526	0.7628617	0.9854749	0.0097152	0.9494869	0.9832552	0.0349085	0.8054582	0.9998749	-0.1489923	0.2586305	0.6603866
ATP5MPL	Mitochondria	0.1480116	0.2379254	0.6083661	0.1253351	0.6210643	0.9854749	0.1443874	0.4092321	0.7656195	0.0383572	0.8407611	0.9998749	-0.2387069	0.1416801	0.4871108
ATP5PB	Mitochondria	0.0173047	0.8561555	0.9633793	0.0368068	0.848395	0.9854749	-0.0192988	0.8931407	0.9722587	0.0494255	0.6769712	0.9998749	0.0152916	0.8988361	0.9660934
ATP5PD	Mitochondria	0.2698069	0.0695676	0.2989928	0.2278239	0.286307	0.9358893	0.2823065	0.1441912	0.5098188	-0.0276823	0.9173148	0.9998749	-0.2466365	0.0451285	0.2978001
ATP5PF	Mitochondria	-0.0188848	0.8668784	0.9644421	-0.0323527	0.8731076	0.9854749	-0.0176476	0.9125158	0.9762162	0.0513414	0.8188884	0.9998749	-0.4251491	0.0111675	0.1174818
ATP5PO	Mitochondria	0.0262045	0.8097852	0.9456563	0.0618385	0.7726603	0.9854749	-0.020305	0.898853	0.9722587	0.1050704	0.5059951	0.9998749	-0.1281498	0.3323104	0.7451529
ATPAF1	Mitochondria	-0.2061346	0.166125	0.499869	-0.1131983	0.6986427	0.9854749	-0.2774524	0.1781421	0.5578491	0.0112899	0.9492804	0.9998749	-0.3252865	0.0851941	
ATPAF2	Mitochondria	0.1534	0.6216125		-0.1019017	0.8519523		0.3160834	0.4722807		0.0370799	0.9157516	0.9998749	0.4329586	0.2782414	
AUH	Mitochondria	-0.0812007	0.7501109		0.0194231	0.9661153		-0.1758832	0.6252034		0.1297014	0.66971	0.9998749	0.0589051	0.8518649	
AURKAIP1	Mitochondria	0.1932424	0.2894734	0.6728314	-0.1447251	0.6305736	0.9854749	0.3791261	0.1052413	0.4490901	0.0907107	0.7379585	0.9998749	0.1366233	0.5767876	

BAD	Mitochondria	0.2111789	0.6602977		-0.4348577	0.6178513		0.6074071	0.3570099		-0.0013732	0.9978988	0.9998749	0.5212667	0.4054612	
BAK1	Mitochondria	0.3616012	0.5941551		0.1745816	0.8744389		0.514501	0.6103748		0.4014156	0.5458379	0.9998749	0.6853108	0.4298912	
BAX	Mitochondria	0.8164744	0.151386		0.8994941	0.3154124		0.7754334	0.372915		0.6703339	0.2494651	0.9998749	0.7101058	0.383202	
BBC3	Mitochondria	1.0360672	0.0128251		0.9495874	0.1894926		1.0650547	0.0559985		-0.1893824	0.6750503	0.9998749	0.0112053	0.9851458	
BCAT2	Mitochondria	0.0670506	0.8005434	0.9456563	-0.1492182	0.7137544		0.1959277	0.5598522	0.8510009	-0.3359247	0.4225976	0.9998749	-0.0879586	0.7907694	
BCKDHA	Mitochondria	-0.2482737	0.2521392	0.6262146	-0.4042641	0.2653626		-0.1577842	0.6172621		0.1442361	0.5893072	0.9998749	0.4633969	0.0781127	
BCKDHB	Mitochondria	-0.4914228	0.0021821	0.025928	-0.425384	0.1638032	0.7221431	-0.5362414	0.0154438	0.1528939	-0.2354849	0.2461317	0.9998749	-0.2355341	0.2111598	
BCKDK	Mitochondria	0.0293797	0.8880561	0.9646758	-0.0409081	0.9116887		0.0646403	0.8287335		0.1266605	0.6173734	0.9998749	0.3227382	0.2215528	
BCL2	Mitochondria	0.8716104	0.0302003		1.1816453	0.0574728		0.594202	0.3058225		1.2657958	0.0015294	0.5561846	0.6792909	0.2319631	
BCL2A1	Mitochondria	1.0351344	0.4660203								1.0892364	0.1217804	0.9998749			
BCL2L1	Mitochondria	0.2025623	0.49944		0.1878727	0.7082868		0.1799349	0.6746847		0.5421889	0.1126506	0.9998749	0.4903223	0.1967837	
BCL2L10	Mitochondria	-0.0676938	0.9079369		0.3134109	0.7688924		-0.2954499	0.7124882		0.0784829	0.9027673	0.9998749	-0.2443856	0.7460493	
BCL2L11	Mitochondria	-0.4602204	0.3852719		-0.1157117	0.8997885		-0.7217865	0.3292703		0.2782956	0.6647452	0.9998749	-0.014561	0.9814099	
BCL2L13	Mitochondria	-0.1355796	0.5306922	0.8267338	-0.239612	0.5219149		-0.0848071	0.7858669		0.0912316	0.748519	0.9998749	0.2797376	0.2883132	
BCL2L2	Mitochondria	0.0163434	0.9389502	0.981362	0.0431594	0.9135877		-0.0108515	0.970703		0.0824862	0.7411096	0.9998749	-0.0272912	0.9202865	
BCO2	Mitochondria	0.2523607	0.1785709	0.5153047	0.0838234	0.7829508	0.9854749	0.3462763	0.1692917	0.5549629	-0.2277232	0.5292345	0.9998749	-0.0156626	0.97124	
BCS1L	Mitochondria	0.1775908	0.6428772		0.147098	0.8446187		0.1935023	0.6980564		-0.3294236	0.4578141	0.9998749	-0.1825889	0.7169138	
BDH1	Mitochondria	0.0255168	0.8102938	0.9456563	-0.1449574	0.436639	0.9506771	0.1148214	0.4220709	0.7809995	-0.2505648	0.1260032	0.9998749	0.0433933	0.7674052	0.9526674
BID	Mitochondria	0.1884458	0.4721902		0.4068148	0.4081207		0.0507274	0.8876942		0.0740049	0.8013057	0.9998749	-0.1720392	0.6196172	

BLOC1S1	Mitochondria	0.0381275	0.8610447	0.9644421	-0.12229	0.7543331		0.1360694	0.6602108		0.2769581	0.2982522	0.9998749	0.1366985	0.6272759	
BNIP3	Mitochondria	-0.2449903	0.0122422	0.0862648	-0.2414684	0.2086003	0.8186655	-0.260362	0.0652247	0.356451	-0.2641081	0.0397358	0.9998749	-0.238611	0.0746879	0.4179347
BNIP3L	Mitochondria	0.3400546	0.0562055	0.2600043	0.6376929	0.0427069	0.4169008	0.15043	0.5160279	0.8259241	0.4163474	0.0777107	0.9998749	-0.1734829	0.4643464	
BOK	Mitochondria	-0.1182482	0.4204454	0.7471844	-0.418229	0.1091128	0.6213367	0.0682273	0.7423364	0.922111	-0.233436	0.2132497	0.9998749	0.2289912	0.3429066	0.751537
BOLA1	Mitochondria	-0.1232402	0.7640658		-0.5496725	0.4868143		0.1051102	0.8450965		-0.4318398	0.3770493	0.9998749	-0.0002684	0.9995838	
BOLA3	Mitochondria	0.3080502	0.3832702		0.2685885	0.6710555		0.3232549	0.5067986		0.2585345	0.4921817	0.9998749	0.0934543	0.8416348	
BPHL	Mitochondria	-0.1272641	0.2736418	0.6554423	-0.1263333	0.5667095	0.9854749	-0.1359829	0.3916543	0.7514299	-0.0599281	0.7555127	0.9998749	-0.0700335	0.6368648	0.9365555
C12orf65	Mitochondria	0.5387857	0.0483802	0.2383612	0.5598575	0.2201425		0.5168951	0.1250895	0.4875534	0.0768111	0.8512749	0.9998749	-0.3983386	0.2163435	
C15orf61	Mitochondria	-0.2865586	0.8796382													
C16orf91	Mitochondria	0.2560172	0.6391533		-0.0452464	0.9626308		0.4376393	0.5627481		0.0267269	0.9644966	0.9998749	0.3790193	0.6009413	
C1QBP	Mitochondria	0.0866109	0.5395057	0.8297981	0.0212248	0.9368935	0.9909626	0.1012441	0.6179116	0.8714138	0.1727845	0.3067243	0.9998749	0.0751397	0.6740711	
C2orf69	Mitochondria	0.3470502	0.0590439	0.269027	0.462041	0.1677626		0.2688065	0.2889613	0.6806876	-0.0023541	0.9914942	0.9998749	-0.1766762	0.4622995	
C3orf33	Mitochondria	-0.0305756	0.9570982		0.1336622	0.8981988		-0.1148767	0.8798225		0.0194684	0.9763485	0.9998749	-0.0572637	0.9368949	
C5orf63	Mitochondria	0.3806032	0.2329124		0.7155426	0.2264439		0.1810063	0.6654496		0.2197325	0.5437036	0.9998749	-0.269854	0.5282964	
C6orf136	Mitochondria	0.535605	0.1658666		0.345195	0.6101229		0.6518467	0.225251		0.0976577	0.8162734	0.9998749	0.4510744	0.3868948	
C8orf82	Mitochondria	-0.155574	0.5733646		-0.5618726	0.2754683		0.08444	0.8213811		-0.3165538	0.3409224	0.9998749	0.2441695	0.4800385	
CA5A	Mitochondria	0.3118063	0.3645683		0.4128844	0.5191973		0.2538582	0.5727646		-0.0087909	0.9814725	0.9998749	-0.1909171	0.6766387	
CA5B	Mitochondria	0.0654197	0.8783269		0.3549586	0.6158818		-0.1684037	0.783223		0.6916074	0.1438614	0.9998749	0.2886158	0.5882496	
CARS2	Mitochondria	0.0095541	0.9704472		-0.0201508	0.9637951		0.0375137	0.9195048		0.1704696	0.5596536	0.9998749	0.3129673	0.347014	

CASP3	Mitochondria	0.1508754	0.5862486		0.2511785	0.6391946		0.0823322	0.8226655		-0.0451766	0.8852667	0.9998749	-0.4483402	0.2333125	
CASP8	Mitochondria	0.7232082	0.0320575		0.8689925	0.1334436		0.6161293	0.1921738		0.3521485	0.3062845	0.9998749	0.2307713	0.6186412	
CASP9	Mitochondria	0.3235107	0.5663105		0.4874007	0.644131		0.2253022	0.7626048		-0.1807629	0.778688	0.9998749	-0.1290636	0.8659161	
CAT	Peroxisome	-0.5064507	3.934E-08	2.384E-06	-0.4301389	0.0024343	0.0665385	-0.5631971	2.491E-05	0.0012332	-0.3162253	0.0309146	0.9998749	-0.4059436	0.0014932	0.0329625
CBR4	Mitochondria	-0.4632624	1.226E-05	0.0003713	-0.2857815	0.1617033	0.7206342	-0.5778901	0.0001662	0.0054857	-0.0338485	0.800901	0.9998749	-0.2504227	0.0452928	0.2978001
CCDC127	Mitochondria	0.1292968	0.6532955		0.2802794	0.5927837		0.0148516	0.9702388		0.0969876	0.764463	0.9998749	-0.0302803	0.9356892	
CCDC51	Mitochondria	0.138058	0.7791948		0.1610064	0.8570709		0.0834955	0.9008813		-0.0331609	0.9516486	0.9998749	-0.0586859	0.9277449	
CCDC58	Mitochondria	0.0739631	0.7253976	0.9456563	0.0039759	0.991972		0.1040615	0.7142957		-0.1471462	0.5570792	0.9998749	-0.1996177	0.4565682	
CCDC90B	Mitochondria	-0.0031462	0.9855806	0.9918134	0.077914	0.8081554	0.9854749	-0.0516646	0.8308481	0.9517856	0.0888832	0.6465496	0.9998749	-0.2659236	0.2883131	
CDK5RAP1	Mitochondria	0.190748	0.4955539		0.1218987	0.8100924		0.2185196	0.5708521		0.1494477	0.6298776	0.9998749	0.1190592	0.7417028	
CEP89	Mitochondria	0.2387733	0.553954		0.0501954	0.9405547		0.3678141	0.5184665		0.199665	0.6549697	0.9998749	0.4399359	0.4019606	
CHCHD1	Mitochondria	0.0368256	0.8961373		-0.1109408	0.8313726		0.1037702	0.7886099		-0.0707941	0.8248544	0.9998749	0.0924194	0.7972662	
CHCHD10	Mitochondria	0.2253308	0.1685377	0.499869	-0.1392311	0.5691545	0.9854749	0.4176818	0.0329287	0.2672079	-0.1677644	0.4817186	0.9998749	0.0245854	0.9079655	0.9660934
CHCHD2	Mitochondria	0.0376659	0.8062173	0.9456563	-0.0712348	0.811137	0.9854749	0.0934784	0.6618436	0.9036861	-0.0008983	0.9960219	0.9998749	0.0198353	0.9189699	
CHCHD3	Mitochondria	0.1784422	0.3554696	0.7245031	0.2870642	0.417397		0.0846651	0.7551722		0.1529419	0.4997939	0.9998749	-0.021989	0.9303217	
CHCHD4	Mitochondria	0.2228277	0.429045		0.3754291	0.4862497		0.1301942	0.7295148		0.0848529	0.7855873	0.9998749	-0.3616025	0.3475635	
CHCHD5	Mitochondria	0.0505153	0.9286886		-0.2281651	0.824109		0.2039738	0.7923062		0.0349575	0.954082	0.9998749	0.2204511	0.7590448	
CHCHD6	Mitochondria	0.112104	0.8507111		0.062762	0.9515258		0.1450181	0.8650169		-0.0199434	0.9771427	0.9998749	0.3545043	0.6449672	
CHCHD7	Mitochondria	0.2264927	0.4673465		0.664806	0.2407695		-0.0529356	0.8998599		0.4768329	0.163412	0.9998749	-0.4006855	0.3367866	
CHDH	Mitochondria	0.3722582	0.0467649	0.2361626	0.3587512	0.151994	0.7094408	0.3699609	0.1274174	0.4889271	-0.0228532	0.9450297	0.9998749	-0.0481694	0.8552256	0.9660934

CHPT1	Mitochondria	-0.1231494	0.2667392	0.6465758	-0.2168922	0.303847	0.9358893	-0.0924015	0.5724915	0.8510009	-0.0272717	0.8481083	0.9998749	-0.0223822	0.8649151	0.9660934
CISD1	Mitochondria	0.1778853	0.2816685	0.6615935	0.0963561	0.7651223		0.2146833	0.3425188	0.7027967	-0.1561358	0.4318356	0.9998749	-0.1313969	0.5484151	
CISD3	Mitochondria	0.0535868	0.7352371	0.9456563	-0.1807943	0.5366434	0.9854749	0.1789546	0.4043781	0.7639967	0.0506425	0.838825	0.9998749	-0.0068026	0.9719467	
CLPB	Mitochondria	-0.0781041	0.844118		-0.1124806	0.870468		-0.0724771	0.8969832		0.2095651	0.6439109	0.9998749	0.3108469	0.5308262	
CLPP	Mitochondria	0.03245	0.8803354	0.9646758	0.0729025	0.8577496		-0.0013219	0.99646		-0.0643235	0.7996482	0.9998749	-0.0781279	0.7869929	
CLPX	Mitochondria	-0.1074988	0.358888	0.7245031	-0.1024954	0.6351489	0.9854749	-0.1432632	0.3985921	0.7588581	0.0091854	0.9642312	0.9998749	0.0727983	0.5779344	0.9150195
CLYBL	Mitochondria	-0.1184007	0.4711702	0.7824687	-0.3152415	0.3141629	0.9358893	-0.0031679	0.9888244	0.9946164	-0.1315047	0.5088505	0.9998749	0.1352765	0.5057566	
CMC1	Mitochondria	0.051823	0.8744318		-0.1165456	0.8449834		0.1289359	0.7764044		-0.1844989	0.6299625	0.9998749	0.1460645	0.7239772	
CMC2	Mitochondria	0.0340482	0.88128		-0.1603331	0.7024008		0.1339933	0.6686679		-0.0241974	0.9278788	0.9998749	0.0702191	0.8090981	
CMC4	Mitochondria	0.0015174	0.9948225		0.0175427	0.9685388		-0.0155853	0.9605102		0.0905123	0.7418315	0.9998749	-0.1597176	0.5908903	
CMPK2	Mitochondria	2.6497097	3.033E-14	9.189E-12	2.1908269	7.782E-05		2.964113	1.936E-11		-0.3953962	0.4062497	0.9998749	0.4339747	0.4210302	
COA1	Mitochondria	-0.0155969	0.9717528		0.3219346	0.7094962		-0.1928926	0.7342878		-0.0235432	0.9625457	0.9998749	-0.5411962	0.3474215	
COA3	Mitochondria	0.1079148	0.5951918	0.8718668	-0.145955	0.7074799		0.2463417	0.3677588		-0.0612665	0.8009227	0.9998749	0.0407451	0.8742321	
COA4	Mitochondria	-0.0161103	0.9421431	0.981362	-0.0814313	0.8452037		0.0129162	0.9660893		-0.0585865	0.8203335	0.9998749	0.0313551	0.910358	
COA5	Mitochondria	-0.030705	0.8737403	0.9646758	-0.0164056	0.9645695		-0.0493951	0.8524562		-0.0238171	0.9165821	0.9998749	-0.1379157	0.5757	
COA6	Mitochondria	0.2126641	0.4298645		0.2237445	0.6723996		0.1953244	0.5822703		-0.198318	0.5220583	0.9998749	-0.3910714	0.2830773	
COA7	Mitochondria	0.0428994	0.9021799		0.0784648	0.8964875		0.0050929	0.9918466		0.2489664	0.531457	0.9998749	0.1988969	0.6557716	
COASY	Mitochondria	-0.013119	0.9592859		-0.3298385	0.4884896		0.1805331	0.6067528		-0.1763249	0.5542449	0.9998749	0.2481055	0.4404075	

COMT	Mitochondria	-0.179431	0.1948083	0.5429961	-0.3418428	0.1761027	0.760022	-0.0934278	0.6106734	0.8714138	-0.1893366	0.4664533	0.9998749	0.0260364	0.9209727	0.9660934
COMTD1	Mitochondria	0.269547	0.5218194		-0.2709562	0.7358966		0.55216	0.3137443		-0.38362	0.4272969	0.9998749	0.0291657	0.9577612	
COQ10A	Mitochondria	-0.188419	0.3538885	0.7245031	-0.2404002	0.566083		-0.1731489	0.5087084	0.8234714	-0.5523767	0.0445414	0.9998749	-0.4216749	0.1146353	
COQ10B	Mitochondria	0.3961698	0.0963654		0.3957146	0.3280247		0.3701474	0.2806354		0.4118626	0.1145027	0.9998749	0.4010111	0.1973975	
COQ2	Mitochondria	0.4778517	0.0481106	0.2383612	0.6474856	0.1507177		0.3618901	0.248216		0.0593986	0.8383537	0.9998749	-0.4150753	0.2020257	
COQ3	Mitochondria	0.0528453	0.8531539		-0.012674	0.9815977		0.0809538	0.8304702		-0.1173342	0.7221896	0.9998749	-0.2274175	0.5389362	
COQ4	Mitochondria	0.1345706	0.648644		-0.0177714	0.9730813		0.2212791	0.5836572		0.1222322	0.7176285	0.9998749	0.1370016	0.7122025	
COQ5	Mitochondria	-0.0935896	0.591816	0.8704867	-0.0094239	0.9767546		-0.1691772	0.4958343	0.8181458	0.1189905	0.567071	0.9998749	0.0346731	0.8727987	
COQ6	Mitochondria	0.1788363	0.5662468		0.0829833	0.8861774		0.2286696	0.5908578		-0.0771913	0.8278924	0.9998749	0.1108985	0.7848212	
COQ7	Mitochondria	-0.0101552	0.9735986		0.1611063	0.7663121		-0.1518097	0.7237093		0.3156823	0.3696683	0.9998749	0.0741486	0.8464962	
COQ8A	Mitochondria	0.1741024	0.1519757	0.4847224	0.138957	0.523753	0.9854749	0.2022762	0.2613449	0.6427056	-0.0877577	0.5668525	0.9998749	-0.0154157	0.933876	0.9660934
COQ8B	Mitochondria	0.1166611	0.8314838		0.4126705	0.6545069		-0.0850396	0.9140644		0.5610172	0.3513191	0.9998749	0.3793957	0.5853349	
COQ9	Mitochondria	0.3042943	0.0121248	0.0862648	0.2094336	0.3815823	0.9464092	0.3544738	0.0401517	0.2802461	0.0374466	0.7905838	0.9998749	0.1175858	0.4577913	0.8432327
COX10	Mitochondria	0.3685945	0.2355771		0.3741191	0.4835074		0.3296642	0.461114		0.3843776	0.2554806	0.9998749	0.2936594	0.4731849	
COX11	Mitochondria	-0.288436	0.0996542	0.3750958	-0.1458828	0.6475068		-0.4023302	0.107472	0.4508359	0.1137164	0.6034866	0.9998749	-0.0640125	0.7639669	
COX14	Mitochondria	0.0973339	0.6947428		-0.1727684	0.7215336		0.2343958	0.4715868		0.0510934	0.8698351	0.9998749	0.0072494	0.9816135	
COX15	Mitochondria	-0.0381296	0.8737658		0.1740362	0.6791725		-0.2132781	0.5315167		0.3621875	0.2020266	0.9998749	0.0122716	0.9672365	
COX16	Mitochondria	-0.0889752	0.502651	0.803711	-0.1324016	0.6107118	0.9854749	-0.0705203	0.7071049	0.9139984	0.0394187	0.8188504	0.9998749	-0.0526434	0.7507768	

COX17	Mitochondria	-0.0840211	0.5490072	0.8401473	-0.0809671	0.7666874	0.9854749	-0.0818829	0.6756698	0.9036861	-0.0042592	0.982202	0.9998749	-0.2747061	0.1345319	
COX18	Mitochondria	-0.0286136	0.9301216		-0.0100503	0.9861325		-0.0461418	0.919133		0.1047248	0.7837149	0.9998749	0.0983643	0.8119527	
COX19	Mitochondria	0.3004094	0.2308306		0.3370443	0.443657		0.2722034	0.4327895		0.2140358	0.4399431	0.9998749	0.090964	0.779055	
COX20	Mitochondria	-0.2078953	0.3828909	0.731203	-0.1273904	0.7770783		-0.2559668	0.4029003		-0.2155969	0.4774487	0.9998749	-0.468024	0.0997044	
COX4I1	Mitochondria	0.2674724	0.0300174	0.1801047	0.2864072	0.2209904	0.8236914	0.254147	0.1557429	0.5280325	0.3078753	0.0839918	0.9998749	0.0230114	0.8859776	0.9660934
COX5A	Mitochondria	0.0423033	0.8146764	0.9456563	-0.0550108	0.8722083		0.0780409	0.7541159	0.922111	0.1324671	0.5406376	0.9998749	0.042099	0.8504036	
COX5B	Mitochondria	-0.0618748	0.6087946	0.8826065	-0.2544523	0.2828976	0.9358893	0.0411797	0.8076735	0.9493368	0.247016	0.2437969	0.9998749	0.1442391	0.3539035	0.7567204
COX6A1	Mitochondria	0.0302472	0.7930382	0.9456563	-0.1831923	0.4345108	0.9506771	0.139505	0.3516282	0.7113638	0.0634875	0.7390873	0.9998749	0.0556768	0.6806973	0.9365555
COX6A2	Mitochondria	0.3405575	0.9079767													
COX6B1	Mitochondria	0.20629	0.1687491	0.499869	-0.0788805	0.7805849	0.9854749	0.3668567	0.0665415	0.356451	0.0650885	0.7723527	0.9998749	0.1881233	0.3110635	
COX6C	Mitochondria	0.1111567	0.3356421	0.713827	0.0781608	0.6738557	0.9854749	0.1195766	0.4394314	0.7881107	0.3059463	0.2645546	0.9998749	-0.3719731	0.0165238	0.1552056
COX7A2	Mitochondria	-0.0115846	0.9401978	0.981362	-0.1510915	0.5959727	0.9854749	0.0455728	0.8348387	0.9517856	0.182459	0.3985416	0.9998749	0.1276055	0.4900712	
COX7A2L	Mitochondria	0.1369133	0.3647911	0.7245031	0.1459831	0.6115587	0.9854749	0.1240388	0.560389	0.8510009	0.0534922	0.75724	0.9998749	0.0310429	0.882174	
COX7B	Mitochondria	0.0368234	0.7697299	0.9456563	-0.076764	0.752113	0.9854749	0.0822549	0.6292408	0.8785326	0.2330491	0.33041	0.9998749	0.0135933	0.9252021	0.9660934
COX7C	Mitochondria	0.0181993	0.8518242	0.9633793	-0.0862841	0.6354906	0.9854749	0.0679602	0.6169093	0.8714138	-0.2090037	0.1325159	0.9998749	-0.2000588	0.0897085	0.4362021
COX8A	Mitochondria	0.2392973	0.3508255		-0.1659137	0.7246954		0.4790333	0.1680767		-0.082402	0.7704976	0.9998749	0.2928813	0.372693	
CPOX	Mitochondria	-0.277743	0.0641938	0.2849268	-0.2580916	0.3688108	0.9464092	-0.3010749	0.1545392	0.5275649	-0.1104588	0.55669	0.9998749	-0.063356	0.7267348	
CPS1	Mitochondria	-0.1525768	0.0961763	0.3712282	0.0018569	0.9902358	0.9970186	-0.2530319	0.0339646	0.271169	-0.3498609	0.0333551	0.9998749	-0.4971535	4.881E-05	0.0060905

CPT1A	Mitochondria	0.4929217	0.0009109	0.0136014	0.406766	0.0596822	0.469108	0.5385658	0.0083345	0.0962193	0.0294006	0.8869299	0.9998749	0.2841659	0.170102	0.5205244
CPT1B	Mitochondria	0.4961246	0.1985377		0.7143968	0.3143732		0.3698354	0.4671473		-0.1294005	0.7650474	0.9998749	-0.2185572	0.6762115	
CPT2	Mitochondria	0.1196026	0.3905474	0.73132	0.1181182	0.6338782	0.9854749	0.1204433	0.5521775	0.8510009	0.2535578	0.1234659	0.9998749	0.3562746	0.0522683	
CRAT	Peroxisome	-0.0559289	0.755391	0.9456563	-0.3609612	0.2791043	0.9358893	0.1219897	0.609549	0.8714138	-0.1709371	0.4308009	0.9998749	0.3286852	0.1718395	
CRLS1	Mitochondria	-0.2031132	0.2267761	0.5923547	-0.1640111	0.5737111	0.9854749	-0.2547118	0.2949297	0.684413	0.234188	0.2835239	0.9998749	0.1345208	0.5000155	
CROT	Peroxisome	0.051072	0.7854858	0.9456563	0.1782036	0.6015421		-0.0326547	0.8980174	0.9722587	0.0276833	0.8980427	0.9998749	-0.1803713	0.4432312	
CRY1	Mitochondria	-0.0317322	0.9005035		0.5599395	0.2027749		-0.4441786	0.2026056		0.4862802	0.0941276	0.9998749	-0.3541644	0.2643857	
CRYZ	Mitochondria	-0.1069957	0.4073912	0.7366398	-0.0038748	0.9852166	0.9970186	-0.1864022	0.2901517	0.6806876	0.0299686	0.8958659	0.9998749	-0.0284528	0.8599385	0.9660934
CS	Mitochondria	0.4104841	0.002586	0.0290203	0.4807848	0.053203	0.4451681	0.3488952	0.073283	0.366415	0.2431649	0.1693406	0.9998749	0.2642887	0.1439499	
CYB5B	Mitochondria	-0.0395334	0.7953498	0.9456563	0.0211717	0.9404356	0.9909626	-0.1034483	0.6351511	0.8831455	0.1318497	0.473221	0.9998749	0.0723798	0.6992108	
CYB5R3	Mitochondria	0.0887198	0.6267307	0.8936442	0.0372487	0.8773558	0.9854749	0.1264671	0.6138068	0.8714138	0.2848305	0.318324	0.9998749	0.4503321	0.1298647	0.4871108
CYC1	Mitochondria	0.1189309	0.4922063	0.7954053	-0.085345	0.7885059	0.9854749	0.2435156	0.316716	0.6998859	0.0615614	0.7480186	0.9998749	0.3025411	0.1853495	
CYCS	Mitochondria	0.3436986	0.0005293	0.0086859	0.4446858	0.0264287	0.3451674	0.2676209	0.0600775	0.3457949	0.1365305	0.4346253	0.9998749	-0.0899799	0.5571458	0.9045021
CYP11A1	Mitochondria	1.1507171	0.0180822		0.7252355	0.395398		1.3859811	0.0372751		-0.0432868	0.9263067	0.9998749	0.3781904	0.6020208	
CYP27A1	Mitochondria	0.0187869	0.8747076	0.9646758	-0.3004223	0.0717242	0.4984223	0.1939102	0.2080729	0.5988145	-0.2463794	0.0813259	0.9998749	0.0644105	0.7601764	0.9526674
D2HGDH	Mitochondria	0.213357	0.5691993		-0.0615055	0.925073		0.4004011	0.4382323		-0.0592314	0.8911174	0.9998749	0.449904	0.3497936	
DAP3	Mitochondria	0.0325204	0.8545914	0.9633793	0.0296427	0.9263088		0.0114693	0.9641863	0.9876706	0.1056064	0.6153543	0.9998749	0.179548	0.4242052	
DARS2	Mitochondria	-0.0791905	0.777126		0.0163492	0.9738582		-0.1795366	0.6479996		0.1310995	0.6848051	0.9998749	0.0635032	0.8537263	
DBI	Mitochondria	0.1326475	0.2238764	0.5873122	-0.0497356	0.8015507	0.9854749	0.221746	0.0960218	0.4278735	-0.4146837	0.0009876	0.5561846	-0.2986999	0.0961967	0.4362021

DBT	Mitochondria	-0.4062633	9.4E-05	0.0020345	-0.2939153	0.1508309	0.7094408	-0.4803855	0.0011168	0.0207802	0.0219049	0.8727956	0.9998749	-0.1094367	0.3509986	0.7567204
DCAKD	Mitochondria	0.2684709	0.2154608	0.5701715	0.190573	0.6274601		0.3095739	0.3030736		0.0202634	0.9344291	0.9998749	0.1086498	0.7001734	
DCXR	Mitochondria	-0.1510001	0.3695234	0.7270492	-0.6032899	0.020637	0.3254303	0.0746424	0.6758442	0.9036861	-0.5433197	0.0466039	0.9998749	-0.1520079	0.5414678	0.8951228
DDX28	Mitochondria	-0.0644062	0.8826336		0.1277971	0.8655812		-0.2039882	0.7391732		0.3515787	0.4807659	0.9998749	0.1819242	0.7390234	
DECR1	Mitochondria	-0.1519527	0.0314703	0.183375	-0.1432814	0.3423364	0.9358893	-0.1756464	0.1065714	0.4508359	0.1087597	0.4540012	0.9998749	-0.0593089	0.5727211	0.9135733
DELE1	Mitochondria	-0.070243	0.6537193	0.914905	-0.0729709	0.8014258	0.9854749	-0.0739939	0.7408716	0.922111	0.1134941	0.5557036	0.9998749	0.1916193	0.3188803	
DGLUCY	Mitochondria	0.4127661	0.0653804	0.2849268	0.297747	0.4445999		0.4735105	0.1393062		0.13134	0.5959908	0.9998749	0.3326918	0.2603133	
DGUOK	Mitochondria	0.1274498	0.7971909		0.0543774	0.9518088		0.1470823	0.825686		-0.2109092	0.7179895	0.9998749	0.0380845	0.9532227	
DHODH	Mitochondria	-0.1497121	0.4138403	0.7420282	-0.4642255	0.1945854		0.0089579	0.968587	0.9876706	-0.3526109	0.2096096	0.9998749	0.0747941	0.7509851	
DHRS1	Mitochondria	-0.3382052	0.0094197	0.0731836	-0.5480254	0.0312906	0.3467332	-0.2323161	0.1958823	0.5841837	-0.4287794	0.0522081	0.9998749	-0.120052	0.4985302	0.8767158
DHRS2	Mitochondria	-0.1777235	0.7662992					-0.1098504	0.85109		-2.6022422	0.0038472	0.8394634	-2.7343537	0.001453	
DHRS4	Peroxisome	-0.1953033	0.165531	0.499869	-0.3963927	0.1488575	0.7094408	-0.0861654	0.6590756	0.9036861	-0.3950284	0.0668264	0.9998749	-0.0803122	0.722764	0.9457061
DHRS7B	Mitochondria	0.3510639	0.0909154	0.3577578	0.2877451	0.4709307		0.3730606	0.1882619		-0.0058674	0.9797332	0.9998749	-0.1038056	0.7115049	
DHTKD1	Mitochondria	-0.3154498	0.0074049	0.0641056	-0.4007887	0.0396815	0.3968148	-0.2788423	0.0594538	0.3457949	-0.0091374	0.9683005	0.9998749	0.0466073	0.7064015	0.9386991
DHX30	Mitochondria	-0.0280384	0.9247454		0.0503182	0.9243151		-0.083581	0.840073		0.1631014	0.6337676	0.9998749	0.0367182	0.9232017	
DIABLO	Mitochondria	0.1020985	0.7079758		0.3137094	0.510914		-0.0682599	0.8593596		0.4973609	0.104341	0.9998749	0.0558802	0.8721829	
DLAT	Mitochondria	0.2823392	0.2327296		0.471216	0.2682053		0.1246885	0.702259		0.2144705	0.4494495	0.9998749	-0.0637092	0.8342378	
DLD	Mitochondria	-0.1502412	0.3406296	0.7142613	0.0131044	0.9527494	0.9964981	-0.2700466	0.2255248	0.6138361	0.2311504	0.3784658	0.9998749	0.1474162	0.4122965	0.7885242

DLST	Mitochondria	0.2967267	0.0398771	0.2101351	0.2393613	0.3736176	0.9464092	0.3205091	0.1233402	0.4869367	0.0087473	0.9618329	0.9998749	0.1493078	0.4330316	
DMAC1	Mitochondria	-0.1394637	0.6242008		-0.4046411	0.4317145		0.0139502	0.9716642		-0.1330929	0.6940358	0.9998749	0.192326	0.5799634	
DMAC2	Mitochondria	0.2195897	0.6471925		0.1339582	0.874978		0.2718956	0.679636		0.0490787	0.9276615	0.9998749	0.0907491	0.8855241	
DMAC2L	Mitochondria	-0.0664834	0.897188		0.0450005	0.95846		-0.16604	0.8207808		0.365396	0.530277	0.9998749	0.2839321	0.660273	
DMGDH	Mitochondria	-0.6728225	6.822E-09	4.593E-07	-0.6035935	0.0018649	0.0546152	-0.7324439	1.61E-05	0.0008856	-0.0976786	0.5807044	0.9998749	-0.1070156	0.3910982	0.7798739
DMPK	Mitochondria	0.2628651	0.5951288		0.3187229	0.7122906		0.2269856	0.7370052		0.1739847	0.7544718	0.9998749	0.0282688	0.9656588	
DNA2	Mitochondria	0.2414493	0.5668911		0.2762128	0.7181801		0.20992	0.7109848		0.0540356	0.9079358	0.9998749	0.0167713	0.975709	
DNAJA3	Mitochondria	-0.0900914	0.6679771	0.9184946	-0.0889763	0.8142539		-0.125565	0.6748222		0.2078283	0.4125539	0.9998749	0.2080203	0.4180002	
DNAJC11	Mitochondria	0.2407602	0.1971273	0.5429961	0.183511	0.5821565		0.2670366	0.3200182	0.7009248	0.138941	0.522976	0.9998749	0.3222799	0.1801569	
DNAJC15	Mitochondria	0.2166267	0.1227802	0.4180046	0.2780263	0.3139729	0.9358893	0.1774475	0.363073	0.724682	0.0061393	0.9697169	0.9998749	-0.1001668	0.5952039	
DNAJC19	Mitochondria	-0.0841873	0.6689854	0.9184946	0.0502963	0.8958511		-0.1629445	0.5390176	0.8443472	-0.1059285	0.6426543	0.9998749	-0.4945629	0.0547831	
DNAJC28	Mitochondria	-0.0307227	0.9444927		0.0681398	0.9370209		-0.0896767	0.875135		-0.2587285	0.6279462	0.9998749	-0.470063	0.4163744	
DNAJC30	Mitochondria	0.0441208	0.902806		0.0508299	0.9377189		0.0224831	0.9635899		0.234765	0.5661543	0.9998749	0.1091264	0.8107194	
DNAJC4	Mitochondria	0.2220622	0.4418088		-0.0496064	0.9239753		0.3977832	0.3199536		-0.0088007	0.9779804	0.9998749	0.2906171	0.4403404	
DNM1L	Mitochondria	0.1097833	0.6632708		0.4290743	0.3520321		-0.0956958	0.7771935		0.1253451	0.6571088	0.9998749	-0.219774	0.4925881	
DTYMK	Mitochondria	-0.1712391	0.777212		-0.4822844	0.639897		0.0028261	0.9974737		0.1532191	0.8205024	0.9998749	0.5344421	0.4726511	
DUS2	Mitochondria	-0.1966293	0.7439881		-0.4822844	0.639897		-0.0115637	0.9894121		-0.1085104	0.8808194	0.9998749	0.5212398	0.4830943	
DUT	Mitochondria	0.2706149	0.2117821	0.5653742	0.4575922	0.2358022		0.1469356	0.6262096		0.2289283	0.3439756	0.9998749	-0.0351398	0.9004927	
EARS2	Mitochondria	0.003558	0.9898543		0.0526808	0.9160291		-0.0440319	0.910675		0.2080117	0.5253637	0.9998749	0.1168547	0.7401084	

ECH1	Peroxisome	-0.115913	0.3570369	0.7245031	-0.3647861	0.0866586	0.5920397	0.0130865	0.9326018	0.9778656	-0.131266	0.3810787	0.9998749	0.05049	0.7873771	0.9587045
ECHDC1	Mitochondria	-0.3715609	0.0521757	0.2528419	0.0023714	0.9940654	0.9970186	-0.6311114	0.0168498	0.1606814	0.406726	0.0683009	0.9998749	-0.1809984	0.5108308	
ECHDC2	Mitochondria	-0.1074203	0.4403013	0.7513739	-0.0033729	0.9890962	0.9970186	-0.1739947	0.3575234	0.7164942	-0.2610403	0.2887716	0.9998749	-0.2958706	0.1003148	0.4397133
ECHDC3	Mitochondria	-0.0227669	0.893187	0.9646758	-0.2365404	0.4934138		0.0873609	0.6909135	0.9088278	-0.1993299	0.4809806	0.9998749	-0.0270176	0.9226684	
ECHS1	Mitochondria	-0.314884	0.0002419	0.0045816	-0.4519533	0.0046221	0.09974	-0.247054	0.0382131	0.2800571	-0.1090728	0.3185483	0.9998749	0.0807607	0.4540557	0.8432327
ECI1	Mitochondria	-0.0331658	0.8671127	0.9644421	-0.3092935	0.3923867		0.1489189	0.5940591		-0.037828	0.8672764	0.9998749	0.2681648	0.3053124	
ECI2	Peroxisome	-0.3068178	0.0034219	0.0363806	-0.3747975	0.0680894	0.489766	-0.2846415	0.0569816	0.3398298	-0.0483396	0.7416756	0.9998749	-0.0209426	0.8705884	0.9660934
ECSIT	Mitochondria	0.0902603	0.782321		-0.0482074	0.9337679		0.1771346	0.6979945		-0.001099	0.9976742	0.9998749	0.2815313	0.5013609	
EFHD1	Mitochondria	-0.3844688	0.2674395		-0.3279802	0.6882566		-0.2709181	0.454337		-2.6823181	0.0011456	0.5561846	-1.4911928	0.0240967	
EHHADH	Peroxisome	-0.4161739	9.568E-07	3.411E-05	-0.24195	0.092641	0.5998205	-0.5336341	1.458E-05	0.0008856	0.0124756	0.9316676	0.9998749	-0.1293788	0.2696995	0.6629064
ELAC2	Mitochondria	0.3474363	0.0953401	0.3703596	0.4094131	0.27542		0.2990121	0.3092224		0.2778266	0.2272388	0.9998749	0.1524317	0.5786382	
ENDOG	Mitochondria	-0.0069551	0.9814776		-0.3511251	0.5597741		0.1597176	0.6770488		-0.1808267	0.6134712	0.9998749	-0.0626822	0.8749121	
EPHX2	Peroxisome	-0.3386851	0.015566	0.103659	-0.2071716	0.3732637	0.9464092	-0.4360893	0.0262373	0.2201266	-0.1369359	0.5174798	0.9998749	-0.3181893	0.0994776	0.4397133
ERAL1	Mitochondria	0.0219139	0.9253049		-0.0055109	0.9896118		0.0216653	0.9475123		0.1223756	0.6519442	0.9998749	0.1626109	0.5816159	
ETFA	Mitochondria	0.0295225	0.7887941	0.9456563	0.1047526	0.6118787	0.9854749	-0.029503	0.8449841	0.9545313	0.0039763	0.9775655	0.9998749	-0.275519	0.0509447	0.3190108
ETFB	Mitochondria	0.2444577	0.155213	0.492456	-0.1948462	0.3286648	0.9358893	0.5002092	0.026228	0.2201266	-0.0643338	0.6773827	0.9998749	0.1841889	0.5445614	0.8951228
ETFBKMT	Mitochondria	-0.1899476	0.6744266		-0.1795427	0.8276239		-0.2165221	0.7215448		-0.1668137	0.7572727	0.9998749	-0.0956446	0.8650317	

ETFDH	Mitochondria	-0.2371722	0.0105398	0.0788531	-0.2461933	0.186856	0.7813025	-0.2491653	0.0613627	0.3491327	-0.1742096	0.2181778	0.9998749	-0.0922043	0.5056489	0.8806998
ETFRF1	Mitochondria	-0.2254589	0.1094992	0.3949793	-0.2223186	0.4017706	0.9464092	-0.2462461	0.2228324	0.6138361	-0.1781627	0.3026191	0.9998749	-0.184695	0.2969809	0.7028402
ETHE1	Mitochondria	-0.2753149	0.5343663		-0.085579	0.9161967		-0.4009771	0.5013384		-0.0384205	0.9411594	0.9998749	-0.2840377	0.6056607	
EXD2	Mitochondria	0.2233078	0.4963699		0.4512535	0.4599147		0.0763159	0.8629205		0.061013	0.8685418	0.9998749	-0.2530412	0.5615117	
EXOG	Mitochondria	0.1783279	0.7712728		0.6161018	0.5780017		-0.1211425	0.8835423		0.7754356	0.2178413	0.9998749	-0.3624965	0.6539202	
FABP1	Mitochondria	0.4730577	5.5E-05	0.0013331	0.3057165	0.0962157	0.5998205	0.5562565	0.0001253	0.0044311	-0.0607421	0.7710441	0.9998749	-0.029145	0.8805607	0.9660934
FAHD1	Mitochondria	-0.0055183	0.9600393	0.988921	-0.0472074	0.8275597	0.9854749	0.0188595	0.9044356	0.9753717	-0.0675303	0.6949008	0.9998749	-0.0871027	0.6678462	0.9365555
FAHD2A	Mitochondria	-0.0371646	0.770295	0.9456563	-0.3053994	0.2096582	0.8186655	0.1210095	0.4941902	0.8181458	-0.1544084	0.3139131	0.9998749	0.130753	0.4584877	0.8432327
FAM136A	Mitochondria	0.0612563	0.70131	0.9340525	0.0548773	0.8580812	0.9854749	0.0567898	0.7972354	0.9463585	-0.0178266	0.9253513	0.9998749	-0.189229	0.3696244	
FAM162A	Mitochondria	-0.1447322	0.2703436	0.6527022	-0.2009966	0.4328018	0.9506771	-0.132866	0.4645461	0.7956759	0.0312094	0.8652989	0.9998749	-0.0672913	0.6751616	0.9365555
FAM185A	Mitochondria	-0.0103576	0.9864545		0.4800656	0.7145905		-0.2665627	0.7280094		-0.215952	0.763185	0.9998749	-1.1507979	0.1788818	
FAM210A	Mitochondria	-0.0740855	0.7485318		-0.0034114	0.9938363		-0.1213828	0.6979596		-0.0976846	0.7252866	0.9998749	-0.2086729	0.4738005	
FAM210B	Mitochondria	-0.2221309	0.0102984	0.0780105	-0.1359855	0.4524172	0.9610935	-0.2769906	0.0261135	0.2201266	-0.0143949	0.9172378	0.9998749	-0.2594218	0.0136475	0.1329368
FARS2	Mitochondria	0.0031784	0.9908512		0.0854738	0.861187		-0.0792566	0.8409071		0.1243518	0.6960394	0.9998749	0.1228902	0.7238925	
FASN	Mitochondria	0.1732728	0.3848913	0.731203	-0.1414576	0.4972396	0.9854749	0.3677999	0.251362	0.6348174	0.0542109	0.8510838	0.9998749	0.384412	0.3742181	0.7798739
FASTK	Mitochondria	0.3537222	0.4445176		0.1610436	0.8498751		0.4626046	0.4550947		-0.0540554	0.9160598	0.9998749	0.2064677	0.7364164	
FASTKD1	Mitochondria	0.0298948	0.9183613		0.003411	0.9948359		0.0366125	0.9274231		-0.0267004	0.9366282	0.9998749	0.1129354	0.7589612	
FASTKD2	Mitochondria	-0.0070837	0.9771664		0.1067067	0.8089132		-0.1003786	0.772511		0.2366241	0.4094497	0.9998749	0.0719675	0.8164344	

FASTKD3	Mitochondria	0.1331285	0.7412819		0.4959536	0.4847108		-0.123117	0.8262671		0.4486669	0.310537	0.9998749	-0.2477924	0.6423551	
FASTKD5	Mitochondria	0.2149953	0.347777		0.1767576	0.6709397		0.2207551	0.4916419		0.0370029	0.8864137	0.9998749	0.0989408	0.7422092	
FBXL4	Mitochondria	-0.2173569	0.4851817		0.0569314	0.9148688		-0.4300903	0.3336927		0.3950067	0.2890727	0.9998749	0.061633	0.8695928	
FDPS	Mitochondria	-0.3368149	0.0547779	0.2553494	-0.291759	0.3099969	0.9358893	-0.3756314	0.0722269	0.3648193	0.169715	0.5993941	0.9998749	-0.1787647	0.4813641	0.8730949
FDX1	Mitochondria	-0.0950561	0.4170045	0.7454418	-0.0053814	0.9814823	0.9970186	-0.1668118	0.3257208	0.7013319	-0.0235442	0.8686719	0.9998749	-0.1601455	0.269278	0.6629064
FDX2	Mitochondria	0.5567381	0.1772901		0.4120378	0.5878513		0.6484431	0.244287		-0.0780613	0.8566985	0.9998749	0.1557829	0.7840069	
FDXR	Mitochondria	0.3549164	0.435893		0.0095582	0.9897396		0.6267429	0.343359		0.2749343	0.5881081	0.9998749	0.8380088	0.1610279	
FECH	Mitochondria	-0.1038779	0.605121	0.8793845	-0.0410323	0.9090786		-0.1776361	0.5396219		0.1669967	0.4944847	0.9998749	0.1962961	0.4220322	
FH	Mitochondria	-0.1309672	0.1284738	0.4254379	-0.1116687	0.529187	0.9854749	-0.1720975	0.1959081	0.5841837	-0.1196314	0.3319317	0.9998749	-0.146375	0.1633023	0.5203616
FHIT	Mitochondria	0.2800217	0.6107789		0.2018619	0.8335002		0.3187408	0.6726142		0.260209	0.6513672	0.9998749	0.5147364	0.4521127	
FIS1	Mitochondria	0.0893506	0.6039066	0.8793845	-0.1458014	0.6330478	0.9854749	0.2471204	0.3214503	0.7009599	0.1467926	0.4538927	0.9998749	0.4483245	0.0409248	
FKBP10	Mitochondria	0.3681026	0.3810811		0.7843858	0.2330253		0.0096411	0.9874924		0.9697933	0.0298851	0.9998749	0.4366685	0.4287322	
FKBP8	Mitochondria	0.184241	0.3609607	0.7245031	0.0422383	0.908285		0.2743698	0.3369666		-0.0336898	0.8896416	0.9998749	0.2520103	0.3593666	
FLAD1	Mitochondria	0.5514675	0.1612806		0.7629359	0.2862714		0.4274817	0.4221572		0.2404684	0.5492036	0.9998749	0.1313548	0.804824	
FMC1	Mitochondria	-0.1798507	0.7557995		-1.1295939	0.359815		0.2028557	0.7742694		-0.4987402	0.4684674	0.9998749	-0.0642098	0.9288121	
FOXRED1	Mitochondria	0.0411773	0.877723		-0.1250714	0.7898082		0.158464	0.6745117		0.0112261	0.9705422	0.9998749	0.3441301	0.3062847	
FPGS	Mitochondria	-0.0630757	0.8639326		-0.2675457	0.7063918		0.0459024	0.9245779		-0.4366565	0.3301917	0.9998749	-0.1337763	0.7759093	
FTH1	Mitochondria	-0.1802956	0.0360914	0.1996881	-0.0735266	0.5915251	0.9854749	-0.2560114	0.035895	0.2733544	0.2237832	0.0574517	0.9998749	-0.0103321	0.9357961	0.9660934
FUNDC1	Mitochondria	0.2665439	0.3614616		0.5413583	0.2925559		0.0719181	0.8605968		0.3190014	0.3129024	0.9998749	0.0226389	0.9533739	

FUNDC2	Mitochondria	0.1443124	0.4304802	0.7496293	0.3237975	0.3312247		0.0262266	0.9177656	0.9778656	0.27411	0.1848644	0.9998749	-0.0899888	0.7052624	
FXN	Mitochondria	-0.0091565	0.9652679	0.988921	-0.1799478	0.6624765		0.072751	0.7955866		-0.3605862	0.15666	0.9998749	-0.1058972	0.689416	
GADD45GIP1	Mitochondria	0.27435	0.1649343	0.499869	0.0531271	0.8790788		0.4287977	0.132462	0.5007432	-0.0356395	0.8661475	0.9998749	0.2592664	0.3527075	
GATB	Mitochondria	0.1144835	0.6307257		0.1813299	0.6692575		0.0525622	0.8750593		0.0527897	0.8450599	0.9998749	0.0446754	0.8821543	
GATC	Mitochondria	-0.0248386	0.8839609	0.9646758	0.0938546	0.7595963	0.9854749	-0.1134639	0.6405564	0.8881665	0.1525613	0.4591322	0.9998749	0.0790161	0.7114861	
GATD3A	Mitochondria	-0.0523113	0.8311963		-0.23278	0.582961		0.0598257	0.8658261		-0.0818992	0.7766572	0.9998749	0.5297041	0.0984472	
GATM	Mitochondria	-0.3317185	0.0037857	0.0385088	-0.245964	0.1550114	0.7123869	-0.3938074	0.0083584	0.0962193	-0.1104342	0.6036534	0.9998749	-0.2465148	0.0960077	0.4362021
GCAT	Mitochondria	-0.1343681	0.5760965	0.8642121	-0.6009312	0.1601045		0.145049	0.6580344		-0.3914452	0.2120503	0.9998749	0.278481	0.4018049	
GCDH	Mitochondria	-0.3518551	0.0044842	0.0431334	-0.5498039	0.0244776	0.3451674	-0.2344291	0.1784218	0.5578491	-0.4621196	0.0137391	0.9998749	-0.1561802	0.517851	0.8901623
GCSH	Mitochondria	-0.2474347	0.0528039	0.2528419	-0.3650709	0.1293911	0.6874549	-0.1943524	0.2401829	0.6298	-0.3663234	0.0307707	0.9998749	-0.3160627	0.0361988	0.2704669
GDAP1	Mitochondria	0.1142146	0.8575504		0.2055417	0.848396		0.0491234	0.9566943		0.066325	0.9248855	0.9998749	0.1512742	0.8505318	
GFER	Mitochondria	0.0838497	0.7706392		-0.0241007	0.9638361		0.1298805	0.7424425		0.0332235	0.9206935	0.9998749	0.0959999	0.7957861	
GFM1	Mitochondria	-0.1804622	0.1871559	0.5277187	-0.0628404	0.8040834	0.9854749	-0.2908891	0.1473884	0.5137836	0.1094729	0.5866383	0.9998749	0.0059759	0.9715146	0.9865186
GFM2	Mitochondria	-0.147205	0.5467741		0.0595773	0.8946997		-0.296412	0.3739045		0.1808959	0.5259188	0.9998749	-0.2545721	0.4053264	
GHITM	Mitochondria	-0.0588791	0.4523064	0.7635032	-0.050316	0.7351623	0.9854749	-0.090343	0.4711059	0.798895	0.0727551	0.5335549	0.9998749	0.0655718	0.4876584	0.8740124
GLDC	Mitochondria	-0.1132647	0.2814386	0.6615935	-0.0335702	0.871075	0.9854749	-0.1669772	0.2491475	0.6348174	-0.0198512	0.9000497	0.9998749	-0.1076949	0.6325711	0.9365555
GLOD4	Mitochondria	-0.0782294	0.5970705	0.8718668	-0.0820857	0.7642439	0.9854749	-0.0896148	0.677521	0.9036861	0.1214076	0.4998788	0.9998749	0.1991843	0.283912	
GLRX2	Mitochondria	0.1729577	0.6638177		0.2095773	0.7886749		0.1256131	0.8095382		0.0102337	0.9816305	0.9998749	-0.3100825	0.5557892	

GLRX5	Mitochondria	-0.0580639	0.6811734	0.9193565	-0.1076331	0.6971291	0.9854749	-0.0395089	0.8414793	0.9531631	-0.0645719	0.7110749	0.9998749	-0.0250412	0.890851	
GLS	Mitochondria	0.7902462	0.0011603	0.0163518	0.8965285	0.0262953		0.7167485	0.023417		0.4399275	0.1174898	0.9998749	0.6896338	0.0738149	
GLS2	Mitochondria	-0.0845652	0.5427028	0.8326022	-0.0446449	0.8647311	0.9854749	-0.1143492	0.5135343	0.8259241	-0.3203249	0.2361222	0.9998749	-0.6512343	0.0001698	0.0060905
GLUD1	Mitochondria	-0.5167207	3.058E-07	1.323E-05	-0.485594	0.0006068	0.0226184	-0.5520786	0.0002405	0.0066142	-0.2278715	0.1474855	0.9998749	-0.2015889	0.0944788	0.4362021
GLUD2	Mitochondria	-0.7335884	0.0001155	0.0024067	-0.5954834	0.0040092	0.0913217	-0.8173972	0.0018102	0.0308981	0.1220633	0.7347527	0.9998749	0.2419425	0.1546891	0.5114342
GLYAT	Mitochondria	-0.2945103	0.0002346	0.0045816	-0.3217295	0.0697628	0.4931509	-0.2855398	0.0150054	0.1515856	-0.313579	0.0218854	0.9998749	-0.3651369	0.002017	0.0363838
GLYCTK	Mitochondria	-0.0383974	0.816834	0.9456563	-0.5418425	0.0676573	0.489766	0.2248067	0.2765747	0.6581947	-0.5461474	0.0274322	0.9998749	0.1596892	0.5016273	
GOLPH3	Mitochondria	0.1119843	0.3873204	0.731203	0.1991732	0.4047147	0.9464092	0.0319815	0.8622265	0.9656156	0.1000206	0.5906883	0.9998749	0.105402	0.5324726	0.8919763
GOT2	Mitochondria	-0.0204118	0.8584568	0.9633793	0.0809173	0.6825011	0.9854749	-0.094461	0.5482198	0.8480275	0.0645391	0.6223478	0.9998749	-0.0022418	0.9894829	0.9932596
GPAM	Mitochondria	-0.3342825	0.0018171	0.0234287	-0.0906646	0.6169522	0.9854749	-0.4889154	0.0002744	0.0067904	0.1381211	0.4667546	0.9998749	-0.2263696	0.2140725	0.5864693
GPD2	Mitochondria	0.1136735	0.7387862		0.4145424	0.4695911		-0.1303664	0.7882157		0.6307472	0.0898522	0.9998749	0.1639037	0.7051911	
GPT2	Mitochondria	0.0168168	0.8944312	0.9646758	0.1361285	0.5714746	0.9854749	-0.0649382	0.7121718	0.9159826	-0.3035634	0.1136377	0.9998749	-0.4058929	0.0074584	0.093407
GPX1	Mitochondria	0.171919	0.4340126	0.7499764	-0.1221389	0.6848645	0.9854749	0.4010667	0.2064642	0.5988145	0.546872	0.0337898	0.9998749	0.7832247	0.009217	
GPX4	Mitochondria	-0.2004972	0.1762111	0.5133844	-0.4331232	0.0307828	0.3467332	-0.060246	0.7725569	0.9311141	-0.0840714	0.6007205	0.9998749	0.0367505	0.8895265	0.9660934
GRHPR	Mitochondria	-0.1744973	0.0848914	0.3384487	-0.3939234	0.0241981	0.3451674	-0.0556845	0.684388	0.9036861	-0.272179	0.0282194	0.9998749	0.0363227	0.8475041	0.9660934
GRPEL1	Mitochondria	0.1084454	0.4302894	0.7496293	-0.0010026	0.9970186	0.9970186	0.1478421	0.4458402	0.7923079	-0.0780031	0.6277593	0.9998749	0.0266649	0.8780016	
GRPEL2	Mitochondria	0.0519995	0.8280118		0.2311822	0.5903252		-0.0833572	0.8024716		0.3004782	0.2628123	0.9998749	-0.0165538	0.9568238	

GRSF1	Mitochondria	0.0622262	0.7125575	0.9355783	0.1862618	0.5394149	0.9854749	-0.0357013	0.8817077	0.9722587	0.1584966	0.4430937	0.9998749	-0.0161494	0.94153	
GSR	Mitochondria	0.1752244	0.3870846	0.731203	0.1008184	0.7803793		0.1945834	0.494136		0.3089359	0.2177321	0.9998749	0.265553	0.2931414	
GSTK1	Peroxisome	0.1637006	0.1438776	0.4720639	0.026529	0.9029747	0.9872524	0.2376313	0.1438655	0.5098188	-0.0024356	0.986344	0.9998749	0.1663214	0.2831255	0.6788078
GSTZ1	Mitochondria	-0.2067219	0.1085476	0.3949793	-0.4180917	0.1159112	0.651008	-0.0982138	0.570142	0.8510009	-0.4186476	0.1169203	0.9998749	-0.2348051	0.4062505	0.7885242
GTPBP10	Mitochondria	-0.0495133	0.7662385	0.9456563	0.0700113	0.8222908	0.9854749	-0.1324275	0.5668867	0.8510009	0.0082672	0.966791	0.9998749	-0.1321769	0.5249176	
GTPBP3	Mitochondria	0.1956972	0.7383745		0.2923753	0.7807918		0.1472743	0.8539852		-0.1464669	0.8285825	0.9998749	0.028344	0.9710694	
GTPBP6	Mitochondria	0.1931908	0.6556368		0.0432067	0.9551222		0.2926237	0.6272696		0.1513082	0.7547393	0.9998749	0.3397205	0.5478379	
GUF1	Mitochondria	0.0891655	0.7358726		0.2612237	0.5839497		-0.0352809	0.9228112		0.1008361	0.741535	0.9998749	-0.0687002	0.838956	
GUK1	Mitochondria	0.0528774	0.7913351	0.9456563	-0.0690471	0.8431787		0.1367281	0.636686		0.4391029	0.0599017	0.9998749	0.4024045	0.1197723	
HADH	Mitochondria	-0.4516992	0.0004288	0.0074246	-0.4855686	0.0277818	0.3451674	-0.4598411	0.0136673	0.1409975	-0.1033799	0.5752589	0.9998749	-0.0510126	0.7222818	0.9457061
HADHA	Mitochondria	-0.04415	0.6162145	0.8891095	-0.002411	0.9888444	0.9970186	-0.0939711	0.4539669	0.7923079	0.1378791	0.367979	0.9998749	0.1271078	0.2190365	0.5908073
HADHB	Mitochondria	-0.029192	0.8146819	0.9456563	0.0168706	0.9383473	0.9909626	-0.0772477	0.6711787	0.9036861	0.2609138	0.153226	0.9998749	0.2715841	0.0929062	0.4362021
HAGH	Mitochondria	-0.1679821	0.215424	0.5701715	-0.2464137	0.3320907	0.9358893	-0.1241784	0.5090551	0.8234714	-0.0825095	0.6562328	0.9998749	-0.0664928	0.7510613	0.9526674
HAO2	Peroxisome	-0.730626	1.664E-07	8.405E-06	-0.948315	1.019E-05	0.001372	-0.6160279	0.0003548	0.0082392	-0.3968956	0.1251659	0.9998749	-0.1714483	0.2421761	0.630617
HARS2	Mitochondria	0.2831777	0.3165411		0.4547273	0.3839806		0.1633982	0.6681949		0.0657026	0.8379036	0.9998749	-0.2183475	0.5635805	
HCCS	Mitochondria	0.265603	0.3866539		0.4934428	0.3862538		0.1136378	0.785839		0.1812577	0.5905122	0.9998749	-0.1595212	0.6957556	
HDHD3	Mitochondria	-0.0290382	0.8911028	0.9646758	-0.0185859	0.9637641		-0.0400094	0.8875402		-0.1447309	0.5895064	0.9998749	-0.0872333	0.7494749	
HDHD5	Mitochondria	0.1431687	0.6287884		-0.1631741	0.7624447		0.3332785	0.4112619		-0.0957153	0.77428	0.9998749	0.3578096	0.3444234	

HEBP1	Mitochondria	-0.0297852	0.8005352	0.9456563	-0.1819062	0.4436038	0.9553296	0.0476676	0.7716316	0.9311141	-0.1169747	0.4351159	0.9998749	-0.0727549	0.6207104	0.9365555
HEMK1	Mitochondria	0.1239717	0.5806485	0.8642121	0.1757272	0.6656755		0.0824328	0.787433		-0.006106	0.9833495	0.9998749	0.1221552	0.6750947	
HIBADH	Mitochondria	-0.0522278	0.5372657	0.8297981	0.0054971	0.975064	0.9970186	0.1145484	0.3765508	0.7338294	-0.1275825	0.3200632	0.9998749	-0.1750071	0.1079452	0.4435874
HIBCH	Mitochondria	-0.3508685	0.0006166	0.0098327	-0.2670342	0.1853505	0.7813025	-0.4063513	0.0070172	0.0919675	0.0836072	0.6423209	0.9998749	0.0301249	0.8805579	0.9660934
HIGD1A	Mitochondria	0.0478375	0.6775034	0.9184946	-0.0391466	0.8495299	0.9854749	0.0723177	0.669679	0.9036861	-0.0105487	0.9596948	0.9998749	0.2302937	0.0839035	0.4243582
HIGD2A	Mitochondria	0.2455848	0.3971964		-0.0264714	0.9609273		0.4014017	0.3080281		-0.0417958	0.8959866	0.9998749	0.2064	0.5862803	
HINT1	Mitochondria	-0.1386371	0.1651508	0.499869	-0.1665573	0.4177002	0.9506771	-0.1352981	0.3414783	0.7027967	-0.1128561	0.4756858	0.9998749	-0.2165823	0.1413769	0.4871108
HINT2	Mitochondria	0.0251673	0.8991615	0.9661203	-0.2096034	0.5890329		0.142297	0.5898082	0.8586913	-0.111382	0.6539161	0.9998749	-0.0394638	0.8751209	
HINT3	Mitochondria	-0.1219701	0.4236655	0.7479033	-0.0209774	0.9415059	0.9909626	-0.2056542	0.3435895	0.7027967	0.1027724	0.5887583	0.9998749	0.0123808	0.9483459	
HMGCL	Peroxisome	0.0029849	0.9819429	0.9918134	-0.0950668	0.6608754	0.9854749	0.0514795	0.7804352	0.9357761	-0.1571747	0.4488902	0.9998749	-0.1850818	0.283912	0.6788078
HMGCS2	Mitochondria	-0.4368119	6.006E-05	0.0013999	-0.1659593	0.3483278	0.9358893	-0.6018406	2.082E-06	0.0002577	-0.1156741	0.4352032	0.9998749	-0.4930906	0.0003766	0.011005
HOGA1	Mitochondria	-0.443397	0.0093338	0.0731836	-0.5670598	0.1008608		-0.3896532	0.0873456	0.4078875	-0.4174291	0.0736319	0.9998749	-0.3299193	0.1242392	
HSCB	Mitochondria	-0.0131324	0.9718605		0.1068708	0.8791534		-0.0859298	0.861815		-0.030623	0.9417145	0.9998749	-0.2920733	0.5420965	
HSD17B10	Mitochondria	-0.1391713	0.3743225	0.731203	-0.3573769	0.2428672	0.8890673	-0.0279847	0.8952544	0.9722587	-0.1189238	0.5337674	0.9998749	0.0772965	0.694997	
HSD17B4	Peroxisome	-0.0879158	0.4057898	0.7366398	-0.0448101	0.7833398	0.9854749	-0.1286061	0.391538	0.7514299	-0.053031	0.7469418	0.9998749	-0.0489733	0.7085237	0.9386991
HSD17B8	Mitochondria	-0.1930692	0.601856		-0.3737771	0.5878351		-0.0835236	0.8667379		-0.0636948	0.8860363	0.9998749	0.1238396	0.7834623	
HSDL1	Mitochondria	0.3994433	0.2204982		0.7489136	0.1907779		0.1586408	0.7222531		0.4008063	0.2581434	0.9998749	-0.184971	0.6763003	

HSDL2	Mitochondria	8.098E-05	0.999441	0.9997657	0.1755368	0.4071104	0.9464092	-0.117822	0.4607705	0.7923079	0.2237798	0.1174983	0.9998749	-0.1389035	0.3343271	0.7451529
HSPA9	Mitochondria	0.0408514	0.5156626	0.8159047	0.0696944	0.6030474	0.9854749	0.0083942	0.9340524	0.9778656	0.0640979	0.5340112	0.9998749	0.0241042	0.7949968	0.9591016
HSPD1	Mitochondria	-0.0635443	0.4284726	0.7496293	0.0201973	0.8953333	0.9854749	-0.1269258	0.2967445	0.684413	0.0516107	0.7144948	0.9998749	0.0099462	0.9159758	0.9660934
HSPE1	Mitochondria	0.0375657	0.7323367	0.9456563	0.0956709	0.6163551	0.9854749	-0.0227855	0.8860313	0.9722587	0.0617136	0.7457533	0.9998749	-0.1685686	0.1702095	0.5205244
HTATIP2	Mitochondria	0.4286025	0.0076398	0.0643015	0.4295532	0.1522702	0.7094408	0.4043386	0.0755885	0.3704584	0.027425	0.8818487	0.9998749	-0.0079299	0.9707993	
HTD2	Mitochondria	0.075321	0.7605918		-0.0772554	0.8684869		0.1399782	0.6654016		-0.0513382	0.8692199	0.9998749	-0.142907	0.6411903	
HTRA2	Mitochondria	0.0109933	0.9830767		-0.192612	0.8383046		0.1194176	0.8641098		0.0172434	0.9770535	0.9998749	0.1398969	0.8325984	
IARS2	Mitochondria	-0.1179839	0.2353562	0.6069186	-0.0619465	0.7578095	0.9854749	-0.1754634	0.2234049	0.6138361	0.0661085	0.5899395	0.9998749	-0.0322032	0.7873117	0.9587045
IBA57	Mitochondria	-0.3944687	0.1865851		-0.5492119	0.3039948		-0.3140984	0.4368171		-0.0199213	0.9585995	0.9998749	0.064305	0.8524698	
IDE	Mitochondria	0.115156	0.6551618		0.1781619	0.6991454		0.0639067	0.8592198		0.3102887	0.2704303	0.9998749	0.1699949	0.6032893	
IDH2	Peroxisome	-0.0051886	0.9654377	0.988921	-0.3384167	0.0936844	0.5998205	0.1966479	0.2105002	0.5988369	0.0350039	0.8552264	0.9998749	0.6128471	0.001504	0.0329625
IDH3A	Mitochondria	0.2682992	0.1183027	0.4101232	0.3684235	0.2582868		0.1898949	0.4244219	0.7809995	0.1059678	0.5847015	0.9998749	-0.067487	0.7656481	
IDH3B	Mitochondria	0.0691293	0.7751002		-0.0280195	0.9486025		0.1123574	0.7402947		0.0726149	0.7995047	0.9998749	0.374048	0.2168189	
IDH3G	Mitochondria	0.3270653	0.1639752		0.2484367	0.5431345		0.3890023	0.2511761		0.245765	0.3192431	0.9998749	0.3645331	0.2436539	
IDI1	Mitochondria	0.0281714	0.8867588	0.9646758	0.2872599	0.265909	0.931818	-0.1461793	0.5762983	0.8540947	0.4589352	0.1647581	0.9998749	-0.0261449	0.9403799	0.9660934
IFI27	Mitochondria	2.6964786	4.737E-18	2.87E-15	2.1871297	1.604E-12	6.577E-10	2.9651966	6.289E-12	3.113E-09	0.32352	0.5794663	0.9998749	0.5976883	0.167357	
IMMP1L	Mitochondria	-0.9453687	0.1786414		-1.060245	0.3479295		-0.9101911	0.3889471		0.2036612	0.8185561	0.9998749	0.3837286	0.5967649	
IMMP2L	Mitochondria	0.0722886	0.8598413		-0.0466834	0.952044		0.1326197	0.8051439		-0.2041555	0.6665932	0.9998749	-0.1528762	0.7707079	
IMMT	Mitochondria	0.3274012	0.0314514	0.183375	0.289202	0.3109553	0.9358893	0.3322955	0.1237939	0.4869367	0.0630001	0.7151466	0.9998749	0.1076346	0.5934551	

ISCA1	Mitochondria	0.1643997	0.4084209	0.7366398	0.3548422	0.3212931		0.0157413	0.9554218		0.2556006	0.2524607	0.9998749	3.847E-05	0.9998801	
ISCA2	Mitochondria	0.0988014	0.8174586		0.257859	0.7386325		-0.0298529	0.9595004		0.1534107	0.7522818	0.9998749	-0.0973994	0.8624067	
ISCU	Mitochondria	0.364125	0.0061705	0.0549898	0.4317112	0.0558134	0.4576696	0.3165149	0.0965025	0.4278735	0.0581177	0.7571897	0.9998749	-0.2330692	0.1429109	0.4871108
ISOC2	Mitochondria	0.0131941	0.9597148		-0.1209675	0.7994661		0.0904436	0.8001728		0.078739	0.7898086	0.9998749	0.1724511	0.5987131	
IVD	Mitochondria	-0.2266243	0.0755505	0.3093486	-0.2609071	0.2870468	0.9358893	-0.2168762	0.2337351	0.6211561	-0.0732669	0.6887441	0.9998749	-0.0116396	0.939191	0.9660934
KMO	Mitochondria	-0.2468264	0.023696	0.1495807	0.0012025	0.9954749	0.9970186	-0.3946526	0.0059206	0.0837336	-0.2733544	0.1020778	0.9998749	-0.4772041	0.0016335	0.0330466
KYAT3	Mitochondria	0.0778467	0.7708674		-0.0035518	0.9943068		0.0997806	0.7861958		-0.0988349	0.7542682	0.9998749	0.025573	0.9404203	
L2HGDH	Mitochondria	-0.5815218	0.004095	0.0406815	-0.5459269	0.1515338		-0.6223711	0.0268506		-0.073268	0.7790407	0.9998749	-0.0952473	0.682259	
LACTB	Mitochondria	0.2429301	0.116773	0.4101232	0.2227955	0.4126555	0.9464092	0.2464519	0.2580466	0.6386654	0.1802657	0.3748833	0.9998749	0.0475567	0.8066281	
LACTB2	Mitochondria	-0.0939587	0.4791188	0.7903206	0.0180726	0.9424757	0.9909626	-0.1914343	0.3181732	0.699981	0.0786674	0.6558438	0.9998749	-0.0215827	0.8994133	0.9660934
LAP3	Mitochondria	0.6176852	9.126E-08	5.027E-06	0.599095	0.0012785	0.0403222	0.6037075	0.000214	0.006383	0.154215	0.5450897	0.9998749	0.3248026	0.0090234	0.103181
LARS2	Mitochondria	0.1437791	0.5643385		0.1470857	0.7384412		0.0982319	0.7823541		0.2550664	0.3792289	0.9998749	0.2415785	0.4470908	
LDHB	Mitochondria	0.3292691	0.2224022		0.7784295	0.0763627		-0.0011527	0.9974271		0.997501	0.0021443	0.5848451	0.4053785	0.2939045	
LDHD	Mitochondria	-0.2038748	0.3291753	0.713693	-0.5140639	0.1840359		-0.0097892	0.972742		-0.2173693	0.3915632	0.9998749	0.2166018	0.4136102	
LETM1	Mitochondria	0.250664	0.1622959	0.499869	0.0993331	0.760293		0.3438738	0.1767996	0.5578491	0.0824819	0.6837205	0.9998749	0.2903685	0.2120347	
LETMD1	Mitochondria	0.1787085	0.5752424		0.1663486	0.7761599		0.16972	0.6929554		0.1269442	0.7232549	0.9998749	0.071385	0.8598766	
LIAS	Mitochondria	-0.2367922	0.4176849		-0.1005023	0.8519145		-0.328518	0.4075946		-0.0395975	0.9110221	0.9998749	-0.2197455	0.5395464	
LIG3	Mitochondria	-0.0551199	0.8777056		-0.2825973	0.6662208		0.0666439	0.8917348		-0.0479703	0.9088507	0.9998749	0.1284355	0.7748945	
LIPT1	Mitochondria	0.1381475	0.7442931		0.3485189	0.6578136		-0.003401	0.9952064		0.1005974	0.8304676	0.9998749	-0.3479627	0.5386182	

LIPT2	Mitochondria	-0.3673396	0.5330169		-0.2440834	0.8196241		-0.4613796	0.567723		0.2555211	0.7071607	0.9998749	-0.0712465	0.9210842	
LONP1	Mitochondria	-0.029519	0.8914854	0.9646758	-0.1254213	0.7358629		0.0206565	0.9481735		0.2127619	0.4332954	0.9998749	0.3669915	0.1944472	
LRPPRC	Mitochondria	-0.2969151	0.0143963	0.0980246	-0.180194	0.4026756	0.9464092	-0.3956714	0.0213736	0.1948992	0.1839444	0.3496773	0.9998749	0.0663647	0.6777695	0.9365555
LYPLA1	Mitochondria	-0.2266903	0.2564648	0.6317793	0.0105145	0.9763213		-0.4097739	0.1498932	0.5152579	0.1076995	0.6736971	0.9998749	-0.1600866	0.501546	
LYPLAL1	Mitochondria	0.1889451	0.5000214		0.4215023	0.4045286		0.0237581	0.9505118		0.1344936	0.6750984	0.9998749	-0.2651685	0.4674969	
LYRM1	Mitochondria	0.159504	0.3434609	0.7177149	0.0321269	0.921657		0.2165591	0.3524976	0.7113638	-0.1373972	0.501472	0.9998749	-0.0378962	0.8661971	
LYRM2	Mitochondria	0.0540572	0.7638006	0.9456563	0.1902357	0.5611072		-0.0472335	0.8524582	0.9576831	0.2051148	0.331608	0.9998749	0.0724215	0.7513809	
LYRM4	Mitochondria	-0.0207161	0.9636665		-0.0645241	0.9342925		-0.0033063	0.9958741		0.4079794	0.4191202	0.9998749	0.3473904	0.5367706	
LYRM7	Mitochondria	-0.0562922	0.8038539	0.9456563	0.0331513	0.9363004		-0.1131815	0.7141259		0.0364457	0.8933572	0.9998749	-0.1286468	0.6484663	
LYRM9	Mitochondria	0.1224934	0.7038935		0.1948338	0.7279625		0.0771422	0.8648859		0.2466033	0.5015046	0.9998749	0.2847766	0.4873364	
MACROD1	Mitochondria	-0.2548368	0.4385639		-0.4382041	0.4705591		-0.1531437	0.730538		-0.1630164	0.6941637	0.9998749	0.1190962	0.7643377	
MAIP1	Mitochondria	-0.1209282	0.5375856	0.8297981	-0.1284357	0.7323184		-0.1390107	0.6079236		-0.1863846	0.4287774	0.9998749	-0.1502616	0.5379371	
MALSU1	Mitochondria	-0.0857455	0.7130126		-0.0164684	0.9699899		-0.1631567	0.6101814		-0.0002612	0.9992339	0.9998749	-0.2274945	0.4319444	
MAOA	Mitochondria	-0.4318981	0.0001191	0.0024067	-0.4578605	0.0270693	0.3451674	-0.4473324	0.0058016	0.0837336	-0.2305862	0.2201499	0.9998749	-0.1217713	0.302503	0.7028402
MAOB	Mitochondria	-0.2308425	0.0092667	0.0731836	-0.2457065	0.1341375	0.6874549	-0.2463105	0.0643883	0.356451	-0.1246749	0.4468285	0.9998749	-0.0339588	0.6908432	0.9365555
MARS2	Mitochondria	0.0099211	0.9835951		0.0710842	0.9346106		-0.0784769	0.9062234		0.3168884	0.5505763	0.9998749	0.2003958	0.7428368	
MAVS	Mitochondria	-0.0933307	0.521563	0.81999	-0.1152398	0.6779145	0.9854749	-0.0864364	0.6705196	0.9036861	-0.0358527	0.8530466	0.9998749	0.0595537	0.7337357	

MCAT	Mitochondria	0.1671002	0.7248709		-0.1705099	0.8500452		0.3345559	0.595018		-0.3684895	0.5020099	0.9998749	0.07472	0.9052122	
MCCC1	Mitochondria	-0.2431672	0.1242154	0.4205281	-0.2540044	0.3362646	0.9358893	-0.2645439	0.2531694	0.6361363	0.2439331	0.2946231	0.9998749	0.2996298	0.0877456	
MCCC2	Mitochondria	-0.1787029	0.0649713	0.2849268	-0.1929782	0.31797	0.9358893	-0.1949792	0.1791879	0.5578491	-0.1820448	0.1865201	0.9998749	-0.0603661	0.6216172	0.9365555
MCEE	Mitochondria	-0.470838	0.0484695		-0.2620021	0.5615421		-0.6064706	0.0609908		-0.1575398	0.5972644	0.9998749	-0.3475794	0.2198467	
MCL1	Mitochondria	0.027341	0.8305435	0.9514354	0.252368	0.2174783	0.8236914	-0.1273405	0.4583922	0.7923079	0.2758169	0.121307	0.9998749	-0.0548031	0.753261	0.9526674
MCRIP2	Mitochondria	0.3006296	0.4894936		-0.1233014	0.8765013		0.5822216	0.3216156		-0.1919956	0.6869517	0.9998749	0.2222419	0.7009522	
MCU	Mitochondria	0.235613	0.4307162		0.3238002	0.5310102		0.1534572	0.7150787		0.3189349	0.3338056	0.9998749	0.276526	0.4744885	
MCUB	Mitochondria	1.107721	0.0812924		1.4529044	0.1410577		0.8835821	0.326821		0.8742455	0.0994635	0.9998749	0.0201761	0.9836737	
MCUR1	Mitochondria	0.2072766	0.1863953	0.5277187	0.2845198	0.3473552	0.9358893	0.1521835	0.4820764	0.8111495	0.0912058	0.6200311	0.9998749	-0.1918161	0.3452485	
MDH2	Mitochondria	0.1587665	0.2334091	0.6044698	0.0396423	0.8659612	0.9854749	0.2135257	0.2561535	0.6375258	0.1495728	0.4277176	0.9998749	0.394582	0.0173225	0.1570975
ME2	Mitochondria	0.4481486	0.0396001	0.2101351	0.6397386	0.0914989		0.2757973	0.3683448		0.3719361	0.1780904	0.9998749	0.2107066	0.4594158	
ME3	Mitochondria	0.1991354	0.7145119		0.4560831	0.6612445		0.0398133	0.9556318		-0.1366967	0.8255066	0.9998749	-0.3713337	0.6134804	
MECR	Mitochondria	0.5238945	0.4289782		0.0255764	0.9821433		0.8518854	0.3588233		0.134209	0.8406361	0.9998749	0.7100716	0.4146971	
METAP1D	Mitochondria	0.1238103	0.7935798		0.2432812	0.772249		0.0328973	0.9592003		0.0240259	0.9655804	0.9998749	0.0314385	0.9580264	
METTL15	Mitochondria	0.2074714	0.4183879		0.2753848	0.5341334		0.1480071	0.6691657		0.1688821	0.5843842	0.9998749	0.0030899	0.9921389	
METTL17	Mitochondria	0.1490526	0.5997449		-0.0016734	0.9974532		0.230675	0.5501757		-0.1073534	0.7451135	0.9998749	0.119675	0.7419086	
METTL4	Mitochondria	0.3994243	0.3832647		0.4941844	0.5417467		0.3082256	0.626115		0.3505859	0.472587	0.9998749	-0.0092396	0.9880982	
METTL5	Mitochondria	0.1432528	0.4885719	0.7937656	0.1673336	0.6604279		0.1122011	0.6960262		0.0020625	0.9928943	0.9998749	-0.1988666	0.4602375	
METTL8	Mitochondria	0.1129286	0.8031505		0.4866441	0.564652		-0.1129363	0.850407		0.1796518	0.7268464	0.9998749	-0.4734888	0.4349251	
MFF	Mitochondria	-0.0638548	0.8043969		-0.1460238	0.7575496		-0.0309655	0.9308857		-0.0731156	0.809891	0.9998749	0.0703947	0.8274871	

MFN1	Mitochondria	-0.0490391	0.8031537	0.9456563	0.1961749	0.5738553		-0.2303309	0.4075704		0.3923367	0.0885593	0.9998749	0.0366089	0.8816143	
MFN2	Mitochondria	0.250005	0.0658247	0.2849268	0.2987713	0.2180509	0.8236914	0.185235	0.3455638	0.7039262	0.0945281	0.6576564	0.9998749	0.1421992	0.3850466	0.7798739
MGME1	Mitochondria	0.4803073	0.0989101		0.2979456	0.5798415		0.5775767	0.1391518		-0.2333353	0.4694745	0.9998749	0.0386438	0.9222216	
MGST1	Mitochondria	-0.14797	0.0610472	0.2760792	-0.1202625	0.4093104	0.9464092	-0.1816219	0.1239475	0.4869367	-0.0124281	0.9373533	0.9998749	0.017341	0.8600491	0.9660934
MGST3	Mitochondria	0.0052369	0.9742341	0.988921	-0.0617721	0.8400956	0.9854749	0.0252426	0.9128761	0.9762162	0.0219595	0.9109171	0.9998749	0.0506782	0.8054834	
MICU1	Mitochondria	-0.3836798	0.0092962	0.0731836	-0.4845881	0.0965565	0.5998205	-0.3445031	0.0925296	0.4202031	-0.2594943	0.1592978	0.9998749	-0.1124749	0.5227757	
MICU2	Mitochondria	0.0097494	0.9524419	0.9849485	0.1048293	0.7252384	0.9854749	-0.0614187	0.7932728	0.9439184	0.1812243	0.3300601	0.9998749	0.0202224	0.9250812	
MICU3	Mitochondria	0.2487344	0.4472205		0.3678594	0.5050904		0.1552469	0.7102898		-0.1208341	0.8129993	0.9998749	-0.2230173	0.5593135	
MIEF1	Mitochondria	0.668576	0.0049548	0.0469158	0.629889	0.0599578	0.469108	0.6898092	0.0225995	0.1997637	-0.2083176	0.5935387	0.9998749	-0.086522	0.7139629	
MIEF2	Mitochondria	0.5949519	0.460126					0.7445033	0.4946187		-0.1711808	0.8217562	0.9998749			
MIGA1	Mitochondria	-0.1323579	0.6952189		0.1664229	0.7831131		-0.3458746	0.4578688		0.2296806	0.5683006	0.9998749	-0.1569365	0.7101685	
MIGA2	Mitochondria	0.5701922	0.1885032		0.7764963	0.3366681		0.4417837	0.4415735		0.2641294	0.5555453	0.9998749	-0.1877243	0.7554565	
MIPEP	Mitochondria	0.0114562	0.9709062		0.2072412	0.7044656		-0.1448125	0.7452686		0.3141405	0.3834666	0.9998749	0.0922237	0.8156487	
MLYCD	Peroxisome	0.0153868	0.9377328	0.981362	-0.2199628	0.5442917		0.1524243	0.5800838		-0.0906988	0.6921218	0.9998749	0.3304217	0.1849331	
MMAA	Mitochondria	-0.112337	0.4483093	0.75887	-0.0378651	0.8944931	0.9854749	-0.1702618	0.4158869	0.7739249	-0.0562597	0.7553392	0.9998749	-0.0762341	0.680339	
MMAB	Mitochondria	0.0784585	0.6194053	0.8915905	-0.1712026	0.5219485	0.9854749	0.205044	0.312015	0.6998859	-0.2220368	0.3989638	0.9998749	0.005649	0.9817699	0.9915424
MMADHC	Mitochondria	-0.1664646	0.1872269	0.5277187	-0.0514808	0.8253903	0.9854749	-0.2643872	0.1592597	0.5362827	-0.0220431	0.8834688	0.9998749	-0.1587951	0.3099979	0.7028402
MMUT	Mitochondria	-0.4656606	5.729E-06	0.0001929	-0.4309229	0.0061815	0.1206858	-0.5050666	0.0008515	0.0168606	-0.1901913	0.2378293	0.9998749	-0.1578762	0.1897421	0.5463351

MOCS1	Mitochondria	-0.0743725	0.8198938		-0.1764881	0.7706446		-0.0177109	0.9681674		-0.15723	0.6850728	0.9998749	0.0558756	0.8919158	
MPC1	Mitochondria	0.3559826	0.0305767	0.1816614	0.2026566	0.3427311	0.9358893	0.4309876	0.047951	0.3043042	-0.524807	0.0928942	0.9998749	-0.1685341	0.4140742	0.7885242
MPC2	Mitochondria	-0.1734687	0.126227	0.4249643	-0.0918102	0.6721302	0.9854749	-0.2428334	0.1391521	0.5064728	-0.1428163	0.3252341	0.9998749	-0.2357293	0.1079159	0.4435874
MPST	Mitochondria	0.1095388	0.5761124	0.8642121	-0.2418308	0.4521249	0.9610935	0.2953138	0.245524	0.6298	-0.1627273	0.5856021	0.9998749	-0.0593273	0.8431613	
MPV17	Peroxisome	0.4307469	0.0683196		0.491048	0.239208		0.3819776	0.2488296		0.3310013	0.1913558	0.9998749	0.1850801	0.5584117	
MPV17L	Peroxisome	0.2018838	0.4771225		0.0994286	0.8474856		0.2614885	0.503436		0.0171636	0.9583957	0.9998749	0.2624686	0.4733396	
MPV17L2	Mitochondria	0.6877716	0.2585584		-0.0947761	0.9277876		1.2134279	0.1580952		-0.271282	0.671464	0.9998749	1.0583987	0.1945852	
MRM1	Mitochondria	-0.0199403	0.97011		0.0684562	0.9419871		-0.0888838	0.9022065		0.2121436	0.7293529	0.9998749	-0.0105751	0.9875903	
MRM2	Mitochondria	0.002664	0.9905587	0.9938387	0.0442161	0.9146292		-0.0264749	0.9324245		0.1568491	0.5415701	0.9998749	-0.0160225	0.9557143	
MRM3	Mitochondria	0.2578954	0.4097918		0.2909951	0.6095696		0.2203289	0.607554		0.1798095	0.6062496	0.9998749	0.0371051	0.9285872	
MRPL1	Mitochondria	0.3273475	0.0748573	0.309199	0.527443	0.1071806	0.6189302	0.1932429	0.4385266	0.7881107	0.4825779	0.0616774	0.9998749	-0.1691948	0.4677834	
MRPL10	Mitochondria	0.0508731	0.8653911		-0.0690669	0.8968094		0.1109103	0.7931282		0.0757152	0.8286323	0.9998749	0.3566233	0.3493581	
MRPL11	Mitochondria	-0.0214209	0.9477908		-0.1973754	0.7474336		0.0771239	0.8613302		0.0380438	0.9203568	0.9998749	-0.0013985	0.9973475	
MRPL12	Mitochondria	0.1649475	0.3817275	0.731203	-0.1016667	0.7675914		0.3372938	0.1978507	0.5864436	0.1209444	0.5788245	0.9998749	0.2682523	0.2817532	
MRPL13	Mitochondria	0.1216282	0.4646745	0.7778806	0.178634	0.5802682		0.0667371	0.7731069	0.9311141	0.0460946	0.8125211	0.9998749	-0.2445857	0.2626117	
MRPL14	Mitochondria	0.2901224	0.4189168		0.0775504	0.9055101		0.4241463	0.3880641		-0.1376045	0.7276548	0.9998749	0.2686034	0.5717347	
MRPL15	Mitochondria	0.2541813	0.1691307	0.499869	0.1922496	0.5856607		0.2584115	0.315478	0.6998859	-0.0352641	0.8721557	0.9998749	-0.0394471	0.871039	
MRPL16	Mitochondria	0.0702218	0.5963491	0.8718668	0.1450169	0.5665519	0.9854749	0.0164438	0.9311736	0.9778656	0.0799059	0.6005264	0.9998749	-0.0212061	0.9048035	
MRPL17	Mitochondria	0.3487327	0.1211581	0.4148123	0.3834975	0.3472531		0.3154646	0.3164944		0.2467242	0.3065614	0.9998749	0.1206158	0.6869578	
MRPL18	Mitochondria	0.0882338	0.6341345	0.8971453	0.1995943	0.5845654		0.0224304	0.9273424	0.9778656	0.0656275	0.7818241	0.9998749	-0.5492441	0.0248052	

MRPL19	Mitochondria	-0.0352262	0.784541	0.9456563	0.0611118	0.7855692	0.9854749	-0.1012201	0.5647878	0.8510009	-0.1045032	0.5824301	0.9998749	-0.2459361	0.0775195	0.4243582
MRPL2	Mitochondria	0.0549874	0.8014257	0.9456563	-0.2159382	0.5920425		0.2200639	0.4628183		-0.0432134	0.863784	0.9998749	0.2408323	0.3800339	
MRPL20	Mitochondria	0.0309553	0.8144958	0.9456563	0.0411827	0.8728963	0.9854749	0.0203317	0.9131073	0.9762162	-0.0113429	0.9490147	0.9998749	-0.2645361	0.1249875	0.4871108
MRPL21	Mitochondria	0.2605884	0.3089107		0.2185764	0.6520711		0.275136	0.4270083		0.1245104	0.65816	0.9998749	-0.1008564	0.769251	
MRPL22	Mitochondria	-0.0067618	0.9773684		-0.0704256	0.8720704		0.0129287	0.9689417		0.0765509	0.7774558	0.9998749	0.1184498	0.6932735	
MRPL23	Mitochondria	-0.0826136	0.7361293		-0.2730839	0.5515154		0.0372517	0.9107169		0.036699	0.8971279	0.9998749	-0.1054327	0.7431063	
MRPL24	Mitochondria	0.0382876	0.7889525	0.9456563	0.0749926	0.7913453	0.9854749	0.0126493	0.9489114	0.9832552	0.0397678	0.8212936	0.9998749	-0.2344795	0.2116979	
MRPL27	Mitochondria	0.2996754	0.2049927		0.0821279	0.8489582		0.4240202	0.1945149		0.1918111	0.467896	0.9998749	0.2035005	0.5106693	
MRPL28	Mitochondria	0.2129604	0.3933102		0.1519731	0.7359106		0.2521169	0.470485		0.1044294	0.7041188	0.9998749	0.1985746	0.5449215	
MRPL3	Mitochondria	-0.0941002	0.4921914	0.7954053	-0.034731	0.8948059	0.9854749	-0.1493286	0.4471985	0.7923079	0.0566123	0.7285054	0.9998749	-0.0162415	0.9240966	
MRPL30	Mitochondria	-0.1353202	0.3640336	0.7245031	0.0630201	0.8175507	0.9854749	-0.2854089	0.1774963	0.5578491	0.2784578	0.1149419	0.9998749	0.055365	0.7609918	
MRPL32	Mitochondria	0.0486822	0.7117443	0.9355783	-0.0335798	0.8965418	0.9854749	0.0768454	0.6796373	0.9036861	-0.0066548	0.9662808	0.9998749	0.0828826	0.6133139	
MRPL33	Mitochondria	-0.1004973	0.6433242	0.9045348	-0.0080161	0.9840117		-0.1706039	0.5710864		0.1887136	0.4557019	0.9998749	-0.1024573	0.7087226	
MRPL34	Mitochondria	-0.0384861	0.8163609	0.9456563	-0.2556331	0.436889	0.9506771	0.0718751	0.7416724	0.922111	-0.1874384	0.3585357	0.9998749	0.0066923	0.9736679	
MRPL35	Mitochondria	-0.0154203	0.9182623	0.9715772	0.1696877	0.5371152	0.9854749	-0.156132	0.4609792	0.7923079	0.1715916	0.353441	0.9998749	-0.0431634	0.8164112	
MRPL36	Mitochondria	0.0334891	0.9030776		-0.1565728	0.7628593		0.141774	0.7026774		0.2116042	0.5110785	0.9998749	0.0476762	0.8923015	
MRPL37	Mitochondria	-0.0485327	0.7418104	0.9456563	-0.0863178	0.7624876	0.9854749	-0.0455896	0.825668	0.9517856	-0.0933551	0.606199	0.9998749	0.0125082	0.9482725	
MRPL38	Mitochondria	0.2644275	0.2809994		-0.0593391	0.8906156		0.4865719	0.1609027		-0.0086927	0.9746845	0.9998749	0.525528	0.100691	

MRPL39	Mitochondria	-0.1116031	0.527824	0.8243849	0.0504585	0.8785709		-0.244201	0.3245853	0.7013319	0.1480238	0.4794713	0.9998749	-0.0871208	0.6879349	
MRPL4	Mitochondria	0.6655882	0.0166225		0.3145279	0.5287125		0.875267	0.0203444		-0.1540912	0.5968329	0.9998749	0.2508136	0.5049632	
MRPL40	Mitochondria	-0.0885752	0.695508		-0.0551239	0.9013254		-0.123119	0.684097		0.0545392	0.8375157	0.9998749	-0.3890025	0.1828421	
MRPL41	Mitochondria	0.0685424	0.7860481		-0.2307567	0.6233354		0.2480869	0.469742		0.210626	0.4869662	0.9998749	0.2231597	0.4889731	
MRPL42	Mitochondria	0.1200059	0.6331405		0.1555779	0.7397194		0.0721632	0.833792		0.0302787	0.9159512	0.9998749	-0.0258711	0.9360831	
MRPL43	Mitochondria	0.0401931	0.8404672	0.957979	-0.0152093	0.9688375		0.0661562	0.8055878		-0.0809814	0.7288188	0.9998749	-0.1271383	0.6208126	
MRPL44	Mitochondria	0.0702595	0.6767086	0.9184946	0.0233083	0.9413125		0.0842612	0.7235337	0.9159826	0.0406221	0.8356697	0.9998749	0.1557063	0.4661478	
MRPL45	Mitochondria	0.0066538	0.9689838	0.988921	0.1986639	0.5423871		-0.1159749	0.6132409	0.8714138	-0.1520574	0.4675886	0.9998749	-0.5149417	0.0184368	
MRPL46	Mitochondria	0.066868	0.6988285	0.9340525	0.1358774	0.6824648		0.0046605	0.9844793	0.9924995	0.0355873	0.8597699	0.9998749	-0.1127335	0.6107878	
MRPL47	Mitochondria	0.1854664	0.4396104		0.1681533	0.6980754		0.1711095	0.610301		0.1736187	0.5117559	0.9998749	0.1282591	0.6772728	
MRPL48	Mitochondria	-0.1660677	0.6370227		0.0291005	0.9609953		-0.3215526	0.5208887		0.629726	0.1175463	0.9998749	0.1560078	0.7170805	
MRPL49	Mitochondria	0.2016584	0.3384293	0.713827	0.1735669	0.6406964		0.1966032	0.5154371		0.1718928	0.5020264	0.9998749	0.2599932	0.3372892	
MRPL50	Mitochondria	0.1636059	0.471009		0.3002207	0.4597425		0.0405894	0.8989132		0.2703013	0.2913326	0.9998749	0.0829705	0.7750985	
MRPL51	Mitochondria	0.1106817	0.3358545	0.713827	0.0227328	0.9193586	0.9909626	0.1500073	0.3666672	0.7247624	0.0363632	0.7987175	0.9998749	0.0267099	0.8528088	0.9660934
MRPL52	Mitochondria	0.2683647	0.490833		0.0990138	0.8816402		0.3857869	0.483285		0.4399203	0.2844406	0.9998749	0.4515042	0.3740535	
MRPL53	Mitochondria	-0.1638038	0.5012277		-0.2388234	0.6196733		-0.1238468	0.6990048		-0.2208537	0.4532769	0.9998749	-0.3278209	0.2935296	
MRPL54	Mitochondria	0.0307899	0.9108553		-0.119991	0.8104698		0.1258588	0.7410072		0.2598561	0.4022429	0.9998749	0.1958337	0.5725915	
MRPL55	Mitochondria	0.1740454	0.6587687		0.281222	0.7236591		0.1226942	0.8069345		-0.3914839	0.3985231	0.9998749	-0.5225513	0.3311949	
MRPL57	Mitochondria	-0.0101197	0.9586511	0.988921	-0.0499518	0.8955295		0.0066537	0.9798902	0.9898891	-0.0251107	0.9139391	0.9998749	-0.1722637	0.4905907	
MRPL58	Mitochondria	0.2179897	0.3583204		0.0378155	0.9338557		0.3029712	0.3468964		-0.1368592	0.6063977	0.9998749	-0.0112072	0.9712904	

MRPL9	Mitochondria	0.1296701	0.5014448	0.803711	0.196532	0.5864152		0.0687005	0.7976247		0.0737205	0.7388447	0.9998749	-0.0519083	0.8356774	
MRPS10	Mitochondria	-0.0615283	0.6876946	0.9260954	0.02837	0.9219871	0.9909626	-0.1278219	0.5541518	0.8510009	0.1052737	0.5628957	0.9998749	-0.0299016	0.8768155	
MRPS11	Mitochondria	0.4799377	0.1311586		0.4787743	0.3913036		0.4787339	0.2743032		0.0946163	0.7840751	0.9998749	0.0979292	0.8201491	
MRPS12	Mitochondria	0.670301	0.3192162		0.6347169	0.5745291		0.719143	0.4552761		0.1337573	0.8412326	0.9998749	0.4828002	0.5964559	
MRPS14	Mitochondria	-0.1928846	0.2451439	0.6189884	-0.2522603	0.4343274		-0.1714862	0.4554937	0.7923079	-0.1732514	0.3901235	0.9998749	-0.1365394	0.507362	
MRPS15	Mitochondria	-0.0830517	0.5223039	0.81999	-0.2119393	0.4089806	0.9464092	-0.0168173	0.9257478	0.9778656	0.108046	0.5339045	0.9998749	0.0496108	0.7527863	0.9526674
MRPS16	Mitochondria	0.1026146	0.4884426	0.7937656	0.0074051	0.9792956	0.9970186	0.1534548	0.4573419	0.7923079	-0.0574813	0.7430657	0.9998749	0.0488624	0.7942324	
MRPS17	Mitochondria	0.1804562	0.4465767		0.1166725	0.7959416		0.1997847	0.5324926		0.0441433	0.8720658	0.9998749	-0.1345705	0.6632415	
MRPS18A	Mitochondria	0.1191703	0.6721627		-0.2992785	0.5410488		0.3966494	0.3233894		0.1084824	0.7276814	0.9998749	0.6675002	0.0597629	
MRPS18B	Mitochondria	-0.0046604	0.9726264	0.988921	-0.0826144	0.7506953	0.9854749	0.0397255	0.8364177	0.9517856	0.1468596	0.3921277	0.9998749	0.0869758	0.6106394	
MRPS18C	Mitochondria	0.351745	0.1027891	0.3804901	0.4562001	0.2618441		0.2685678	0.3611501		0.2028319	0.4523043	0.9998749	-0.2504233	0.383161	
MRPS2	Mitochondria	0.2666612	0.2909627		-0.1595538	0.7185517		0.5428644	0.1242474		-0.2257321	0.4226288	0.9998749	0.4373249	0.1775154	
MRPS21	Mitochondria	0.1254599	0.4811587	0.7903206	0.088519	0.7915895		0.1349019	0.5884325	0.8586913	0.0763345	0.7085541	0.9998749	0.0206549	0.9284892	
MRPS22	Mitochondria	-0.0128321	0.9414593	0.981362	0.0680474	0.8290672	0.9854749	-0.0910571	0.7173301	0.9159826	0.1636354	0.4175235	0.9998749	-0.0233501	0.9172464	
MRPS23	Mitochondria	0.1600628	0.5092527		0.1769621	0.6856132		0.1209104	0.7241675		0.2553788	0.3407329	0.9998749	0.0888879	0.7776523	
MRPS24	Mitochondria	-0.110627	0.7010661		-0.3747104	0.4963298		0.023247	0.9513832		-0.0578399	0.8687199	0.9998749	-0.0214765	0.9520653	
MRPS25	Mitochondria	0.3356976	0.0142392	0.0980246	0.3626735	0.174569	0.760022	0.3124977	0.0968118	0.4278735	-0.0807769	0.6360285	0.9998749	-0.1654361	0.3696751	
MRPS26	Mitochondria	-0.0609878	0.8268577		-0.122458	0.8075772		-0.0290055	0.9402931		0.1343342	0.6750342	0.9998749	0.1908916	0.5810091	
MRPS27	Mitochondria	-0.0943879	0.665063	0.9184946	0.0486451	0.8953437		-0.2271791	0.4744324		0.297261	0.2612916	0.9998749	0.2343276	0.3728766	

MRPS28	Mitochondria	-0.1109991	0.5527134	0.8415686	-0.1501196	0.6664801		-0.0930324	0.7168741	0.9159826	-0.0871904	0.6801285	0.9998749	-0.1390268	0.5637712	
MRPS30	Mitochondria	0.1946305	0.4536586		0.1598189	0.7456891		0.1968524	0.5759296		-0.081403	0.7810256	0.9998749	-0.1214799	0.7226195	
MRPS31	Mitochondria	0.1595889	0.279528	0.6615935	0.2643175	0.3488171	0.9358893	0.0851002	0.6806784	0.9036861	-0.0012695	0.9942594	0.9998749	-0.1559489	0.4129407	
MRPS33	Mitochondria	0.0646153	0.7636121	0.9456563	0.1274721	0.7528599		0.0166561	0.9541848		0.04532	0.8588845	0.9998749	-0.2528449	0.3561596	
MRPS34	Mitochondria	0.296604	0.104311	0.3831057	0.2349868	0.4708852		0.3352796	0.1890408	0.572091	-0.0460825	0.8281249	0.9998749	-0.1608164	0.502514	
MRPS35	Mitochondria	-0.0788383	0.5086826	0.8090857	-0.0319511	0.8881358	0.9854749	-0.1293677	0.460881	0.7923079	0.0531047	0.7208488	0.9998749	-0.0452009	0.7679296	0.9526674
MRPS36	Mitochondria	-0.1225824	0.5960826		-0.0783676	0.8588928		-0.1662771	0.5972869		0.0050046	0.9852934	0.9998749	-0.3065421	0.2916847	
MRPS5	Mitochondria	0.4530967	0.0175634	0.1144452	0.3107962	0.2732169	0.9358893	0.5268413	0.0308609	0.2546024	-0.1146557	0.7527444	0.9998749	-0.1211167	0.5191138	
MRPS6	Mitochondria	0.0645567	0.6218415	0.8929762	0.0391622	0.8833857	0.9854749	0.0701182	0.6927381	0.9088278	-0.0972336	0.5631745	0.9998749	-0.2017646	0.2345813	0.6193639
MRPS7	Mitochondria	0.0549333	0.7638217	0.9456563	-0.002302	0.9946546		0.0755701	0.7673767	0.9311141	3.375E-05	0.9998749	0.9998749	0.0908035	0.698757	
MRPS9	Mitochondria	-0.00546	0.9700855	0.988921	0.1291096	0.6434656	0.9854749	-0.0988695	0.6300587	0.8785326	0.0557039	0.7465629	0.9998749	-0.1216024	0.5135352	
MRRF	Mitochondria	0.1893029	0.4287814		0.3961967	0.3752261		0.0621624	0.8455504		-0.0992757	0.7278273	0.9998749	-0.2582444	0.4000283	
MRS2	Mitochondria	-0.1886806	0.4644522		-0.1106414	0.8178239		-0.2446016	0.4870564		0.044948	0.8845236	0.9998749	-0.0827284	0.7946303	
MSRA	Mitochondria	0.0562277	0.7667624	0.9456563	-0.1110612	0.7567745		0.1362947	0.6042925	0.8695488	-0.0381724	0.8644157	0.9998749	0.1086288	0.6489385	
MSRB2	Mitochondria	0.1420229	0.5389334	0.8297981	0.0202084	0.9629048		0.2049947	0.5113207		0.0038584	0.9887201	0.9998749	-0.0068104	0.981723	
MSRB3	Mitochondria	0.2915756	0.5053832		0.0011838	0.9986023		0.4779967	0.4048508		0.0404454	0.93654	0.9998749	1.1191376	0.0599438	
MTCH1	Mitochondria	0.181	0.3273942	0.713693	0.0980999	0.7754133		0.2216728	0.3847107	0.7467914	0.0757035	0.7237724	0.9998749	0.0967669	0.6809462	
MTCH2	Mitochondria	-0.0380218	0.7846073	0.9456563	-0.0953016	0.7280474	0.9854749	-0.0112528	0.9536178	0.9854714	-0.0644022	0.7039283	0.9998749	-0.0859388	0.61759	
MTERF1	Mitochondria	-0.0728036	0.827123		-0.1238598	0.8313903		-0.0599326	0.8986759		0.0478175	0.901951	0.9998749	0.2290071	0.5806869	
MTERF2	Mitochondria	0.1443379	0.4689648	0.7824687	0.3890704	0.306344		-0.0018561	0.994446	0.9946164	0.1173319	0.6137474	0.9998749	-0.3374841	0.1958543	

MTERF3	Mitochondria	0.0824506	0.8027382		0.2071433	0.7384032		-0.0024524	0.9955745		-0.0532806	0.8884518	0.9998749	-0.2865193	0.5075951	
MTERF4	Mitochondria	0.102032	0.6263218	0.8936442	0.0525152	0.8913613		0.1178665	0.6813398		0.1956292	0.426971	0.9998749	-0.0066725	0.9799756	
MTFMT	Mitochondria	0.0320403	0.9137691		0.0519876	0.9246859		0.0078907	0.984351		0.0076029	0.9825502	0.9998749	-0.0458218	0.9038818	
MTFP1	Mitochondria	1.2352326	0.0912577		0.6184618	0.5755675		1.6401854	0.1417554		0.5697681	0.3775948	0.9998749			
MTFR1	Mitochondria	-0.2283087	0.237207	0.6083661	-0.2124864	0.5386425		-0.2661259	0.3376259	0.7027967	0.0400757	0.8666795	0.9998749	-0.0156581	0.9467896	
MTFR1L	Mitochondria	-0.0219525	0.9049398	0.96821	0.0040646	0.9905482		-0.0398971	0.873846	0.9698515	-0.1032368	0.6323842	0.9998749	-0.1580285	0.4807169	
MTG1	Mitochondria	0.2095145	0.3174762	0.6996021	0.1919006	0.6268062		0.2162566	0.4476304		-0.1476635	0.5522544	0.9998749	-0.0374357	0.8900788	
MTG2	Mitochondria	0.3948506	0.1305078		0.4537234	0.3189705		0.3549213	0.3331324		0.1492941	0.5989021	0.9998749	0.2600939	0.4514678	
MTHFD1L	Mitochondria	1.3832348	0.3485137								0.4920053	0.5125929	0.9998749			
MTHFD2	Mitochondria	1.4051644	0.0126044		0.8841397	0.293349		1.751441	0.0276877		0.4576286	0.3649068	0.9998749	1.6744848	0.0502355	
MTHFD2L	Mitochondria	-0.3929352	0.1987697		-0.2756702	0.6367891		-0.4672129	0.2422149		-0.2448575	0.503042	0.9998749	-0.4436755	0.2306078	
MTHFS	Mitochondria	0.0699266	0.649723	0.911417	-0.0644876	0.8313381	0.9854749	0.1344803	0.5221066	0.8310057	-0.148863	0.4427223	0.9998749	-0.0524252	0.7922735	
MTIF2	Mitochondria	0.3206213	0.0816736	0.3296323	0.4402054	0.1181173	0.6544337	0.2403248	0.3165639	0.6998859	-0.0113678	0.9694004	0.9998749	-0.3352158	0.0784963	
MTIF3	Mitochondria	0.3325796	0.0983951	0.3738315	0.1789367	0.5165388	0.9854749	0.4153023	0.1218377	0.4869367	0.0093762	0.9772599	0.9998749	-0.1379189	0.5585453	
MTO1	Mitochondria	0.0234259	0.9022996	0.9677762	0.1181197	0.7311345		-0.0522948	0.8484472		0.1532944	0.4930906	0.9998749	0.0098084	0.9684382	
MTPAP	Mitochondria	0.0049484	0.9822517	0.9918134	0.1189344	0.7704333		-0.0877332	0.7768164		0.0868054	0.7398252	0.9998749	0.0211078	0.9399731	
MTRF1	Mitochondria	0.181715	0.4890299		0.3948976	0.4081654		0.0390958	0.913016		0.1216317	0.6857087	0.9998749	-0.1515623	0.6564085	
MTRF1L	Mitochondria	0.3643186	0.1597029		0.4798741	0.3172873		0.2871507	0.4020332		0.0326013	0.913406	0.9998749	-0.2506454	0.4605352	
MTX1	Mitochondria	0.0158679	0.9598173		-0.4303481	0.4632036		0.2672455	0.5310093		-0.4031122	0.2745661	0.9998749	0.2612426	0.509829	
MTX2	Mitochondria	0.1180156	0.5829167	0.8642121	0.1413106	0.7207314		0.0886785	0.76705		-0.0007808	0.9974892	0.9998749	0.0591549	0.8303508	

MTX3	Mitochondria	-0.173914	0.6023296		-0.1285089	0.8239002		-0.2203141	0.6401737		0.1482553	0.7074187	0.9998749	0.1562696	0.701545	
MUL1	Mitochondria	0.5269464	0.0347459		0.4407582	0.3121237		0.5650075	0.1132779		0.1430438	0.6056508	0.9998749	0.3680786	0.2737988	
MUTYH	Mitochondria	0.8151312	0.6021196								0.4292146	0.600128	0.9998749			
MYO19	Mitochondria	0.3926961	0.3025359		0.4101811	0.5302574		0.3768644	0.48326		0.1624487	0.6921363	0.9998749	0.4516325	0.3656213	
NADK2	Mitochondria	-0.3297211	0.0097537	0.0748197	-0.3423353	0.0628522	0.469108	-0.3365666	0.0673327	0.356451	-0.1928685	0.2236054	0.9998749	-0.1283714	0.4905625	0.8740124
NAGS	Mitochondria	-0.2675664	0.3007429		-0.4283018	0.3804063		-0.1775456	0.610834		-0.272926	0.4037739	0.9998749	0.0868148	0.7829136	
NARS2	Mitochondria	-0.2177748	0.3842933		-0.1125716	0.8129678		-0.293944	0.3860437		-0.1144473	0.7060604	0.9998749	-0.2490092	0.4238169	
NAXD	Mitochondria	0.1114403	0.586124	0.8642121	0.2734738	0.4940777		0.022828	0.9300006	0.9778656	-0.3342982	0.229141	0.9998749	-0.7174848	0.0059907	
NAXE	Mitochondria	0.0909402	0.6738699	0.9184946	-0.1400614	0.726448		0.221535	0.4535722		0.1693115	0.5148468	0.9998749	0.2134811	0.4333981	
NBR1	Mitochondria	-0.0423839	0.7388386	0.9456563	0.1929418	0.3688484	0.9464092	-0.1904181	0.2512268	0.6348174	0.0725153	0.6465342	0.9998749	-0.3688103	0.0370221	0.2704669
NDUFA1	Mitochondria	-0.0310016	0.8409981	0.957979	-0.1870578	0.5146178	0.9854749	0.0469591	0.8261431	0.9517856	0.3653455	0.1602361	0.9998749	0.0295292	0.876	
NDUFA10	Mitochondria	-0.0515227	0.6961426	0.933324	-0.0118616	0.9603305	0.9970186	-0.0976784	0.6228406	0.8758696	0.328355	0.1007732	0.9998749	0.3005899	0.082453	0.4243582
NDUFA11	Mitochondria	0.1580421	0.5499865		-0.1389947	0.7666493		0.3519887	0.3369095		0.0903834	0.7497687	0.9998749	0.3791072	0.2611378	
NDUFA12	Mitochondria	-0.0224166	0.8947353	0.9646758	-0.0912635	0.7727071	0.9854749	-0.0125362	0.9581063	0.9876706	0.1095419	0.6190896	0.9998749	-0.0198615	0.923579	
NDUFA13	Mitochondria	0.0401785	0.7982927	0.9456563	-0.2439702	0.3771368	0.9464092	0.2300606	0.3098048	0.6998859	0.3203623	0.1771851	0.9998749	0.2955475	0.1941725	
NDUFA2	Mitochondria	0.0661176	0.788474		-0.0523619	0.9094918		0.131314	0.6944399		0.1643659	0.567974	0.9998749	0.0319642	0.9184395	
NDUFA3	Mitochondria	0.3963075	0.1677521		0.1317999	0.8004305		0.5624935	0.1541629		0.0177775	0.9533875	0.9998749	0.2405028	0.531278	
NDUFA4	Mitochondria	0.0798819	0.5796706	0.8642121	-0.0426133	0.873716	0.9854749	0.1246811	0.5442387	0.845962	0.1478598	0.518415	0.9998749	0.0454208	0.8007289	

NDUFA5	Mitochondria	-0.0368823	0.7931918	0.9456563	0.137304	0.608391	0.9854749	-0.153633	0.4361416	0.7881107	-0.0390219	0.8138559	0.9998749	-0.3135907	0.0828113	
NDUFA6	Mitochondria	0.1393466	0.2052425	0.5552542	0.117689	0.5993725	0.9854749	0.1356956	0.3884011	0.75101	0.1887665	0.3601766	0.9998749	-0.1848803	0.1911134	0.5463351
NDUFA7	Mitochondria	0.3093303	0.1850586		0.2718789	0.5437361		0.3218777	0.3069452		0.1251459	0.6526383	0.9998749	-0.131988	0.6728351	
NDUFA8	Mitochondria	0.286541	0.1585778	0.499869	0.1978047	0.5990812		0.3078213	0.2809555		0.020588	0.9282889	0.9998749	0.0969697	0.7160293	
NDUFA9	Mitochondria	0.0220184	0.9064266	0.96821	-0.1105672	0.7473651		0.0879234	0.7405126		0.0814454	0.7029891	0.9998749	0.2541274	0.2763899	
NDUFAB1	Mitochondria	0.2000244	0.1183696	0.4101232	0.1884554	0.4105627	0.9464092	0.1763885	0.3370796	0.7027967	-0.1823587	0.3779833	0.9998749	-0.3351068	0.0554696	0.335375
NDUFAF1	Mitochondria	-0.0998471	0.6263443	0.8936442	-0.144757	0.7037379		-0.1015688	0.7248335		-0.060058	0.8038267	0.9998749	0.0070421	0.977765	
NDUFAF2	Mitochondria	0.1881993	0.3475405	0.7237441	0.0317607	0.9318925		0.2759825	0.3154511		0.2549893	0.3366842	0.9998749	0.0412228	0.8740598	
NDUFAF3	Mitochondria	0.1207346	0.7641599		-0.0023069	0.9975072		0.191541	0.7238352		0.0593507	0.8939036	0.9998749	0.230074	0.6501156	
NDUFAF4	Mitochondria	0.2337058	0.3883912		0.2487587	0.623317		0.2018486	0.5850048		-0.0324553	0.9144845	0.9998749	-0.0724083	0.8387821	
NDUFAF5	Mitochondria	0.1297868	0.7901511		0.4976482	0.5722986		-0.1112845	0.8650093		0.2400513	0.6539823	0.9998749	-0.3087537	0.6357279	
NDUFAF6	Mitochondria	0.2812532	0.2849415		0.0253763	0.9581372		0.4133153	0.2396079		-0.2165821	0.4939787	0.9998749	0.0316915	0.9254847	
NDUFAF7	Mitochondria	0.2580939	0.50031		0.3975762	0.5548688		0.137862	0.793457		0.4431571	0.2929884	0.9998749	0.058147	0.9058793	
NDUFAF8	Mitochondria	-0.2383476	0.3895556		-0.4215387	0.4048942		-0.1499108	0.6843288		0.2128913	0.5634692	0.9998749	-0.0505558	0.8806923	
NDUFB1	Mitochondria	0.0878052	0.4712889	0.7824687	0.0676135	0.7801295	0.9854749	0.0873364	0.5974419	0.8647185	0.2523807	0.389937	0.9998749	-0.399727	0.011762	0.118977
NDUFB10	Mitochondria	-0.0416317	0.7559551	0.9456563	-0.1754747	0.5089526	0.9854749	0.0235329	0.8995848	0.9722587	0.0525399	0.7715225	0.9998749	-0.0447795	0.7939182	
NDUFB11	Mitochondria	0.0708177	0.7654532		-0.1794478	0.6872877		0.2223828	0.4920639		-0.0587252	0.8267693	0.9998749	0.0526396	0.8660723	
NDUFB2	Mitochondria	0.0029617	0.9869034	0.9918134	-0.1770601	0.6129614		0.0944391	0.6995506	0.9112567	0.0759605	0.7527596	0.9998749	-0.0156332	0.9450336	
NDUFB3	Mitochondria	-0.1498208	0.5136557	0.814857	-0.2118768	0.5951597		-0.108658	0.7317982		0.1429291	0.571643	0.9998749	-0.0239285	0.9364811	

NDUFB4	Mitochondria	-0.0144546	0.9424962	0.981362	0.0104323	0.9778675		-0.0565813	0.8394114		0.0476142	0.8382696	0.9998749	-0.0756784	0.7634297	
NDUFB5	Mitochondria	0.0746061	0.6673105	0.9184946	0.1195197	0.7124024		0.0272472	0.9110212	0.9762162	0.0806359	0.6839443	0.9998749	-0.0478609	0.8286353	
NDUFB6	Mitochondria	-0.2025048	0.4917769		-0.241125	0.651996		-0.1987019	0.6256757		-0.0478442	0.8911472	0.9998749	0.0815002	0.8188095	
NDUFB7	Mitochondria	0.1214813	0.4885029	0.7937656	-0.1026841	0.744202	0.9854749	0.2752817	0.2661379	0.6427056	0.3196722	0.2343798	0.9998749	0.0548994	0.839196	
NDUFB8	Mitochondria	-0.0334501	0.8145288	0.9456563	-0.1815585	0.491751	0.9835019	0.0412497	0.8391102	0.9526595	0.0411396	0.8040521	0.9998749	0.1903629	0.2717389	
NDUFB9	Mitochondria	0.056405	0.6353701	0.8971453	-0.0933997	0.6824817	0.9854749	0.1307619	0.4235617	0.7809995	0.0642509	0.741996	0.9998749	-0.0751452	0.5922651	0.9216907
NDUFC1	Mitochondria	-0.4336735	0.1053816		-0.464047	0.3613778		-0.4227122	0.2369652		0.00764	0.9810273	0.9998749	-0.1976279	0.5340982	
NDUFC2	Mitochondria	-0.0517624	0.6583243	0.9184946	-0.1622086	0.5138524	0.9854749	-0.0074907	0.9628515	0.9876706	-0.2847612	0.0681547	0.9998749	-0.2346752	0.1330696	0.4871108
NDUFS1	Mitochondria	-0.2022348	0.2016045	0.5503258	-0.0883431	0.7470928	0.9854749	-0.318032	0.1755323	0.5578491	0.2612201	0.2326084	0.9998749	0.0710729	0.7088594	
NDUFS2	Mitochondria	0.1349616	0.2087779	0.5623085	0.0070345	0.9735788	0.9970186	0.1921279	0.2201999	0.6138361	0.1320408	0.2952576	0.9998749	0.2594632	0.0561084	0.335375
NDUFS3	Mitochondria	0.1783837	0.2597649	0.637318	-0.0107533	0.9721011	0.9970186	0.2744577	0.2099662	0.5988369	-0.0950147	0.6015974	0.9998749	0.0541731	0.7926567	
NDUFS4	Mitochondria	-0.0171958	0.9081247	0.96821	0.0381473	0.8929607	0.9854749	-0.0699543	0.7203397	0.9159826	0.2085258	0.3140198	0.9998749	-0.265543	0.1439144	0.4871108
NDUFS5	Mitochondria	0.1478922	0.1720798	0.50377	0.067527	0.741298	0.9854749	0.1762592	0.262502	0.6427056	0.200431	0.4014148	0.9998749	-0.1474184	0.4165794	0.7885242
NDUFS6	Mitochondria	0.3160825	0.0373871	0.2022909	0.1125132	0.6782583	0.9854749	0.4419041	0.0446171	0.2944727	0.1737499	0.3784946	0.9998749	0.13935	0.5181802	
NDUFS7	Mitochondria	0.1067272	0.7420472		-0.2714942	0.6180526		0.4161603	0.3800392		0.2251675	0.5209517	0.9998749	0.6594594	0.1149744	
NDUFS8	Mitochondria	0.2596883	0.4094058		-0.1538669	0.7851826		0.5256863	0.2244251		-0.0558987	0.8695242	0.9998749	0.4726579	0.24469	
NDUFV1	Mitochondria	0.0474722	0.6955576	0.933324	-0.082138	0.6911979	0.9854749	0.1451523	0.4351781	0.7881107	0.0674671	0.6653491	0.9998749	0.224636	0.3059624	0.7028402
NDUFV2	Mitochondria	-0.0956351	0.2932939	0.6760327	-0.1348997	0.4678977	0.96694	-0.0936656	0.4931019	0.8181458	0.0155267	0.9091913	0.9998749	-0.0056258	0.9581871	0.9767566

NDUFV3	Mitochondria	0.1153173	0.519005	0.8190548	0.1624043	0.6359653		0.0770436	0.7544545	0.922111	0.0527523	0.7991883	0.9998749	-0.1522895	0.5164051	
NEU4	Mitochondria	0.0376399	0.9387847		-0.3697857	0.7219029		0.187866	0.7575301		-1.4690564	0.0313559	0.9998749	-0.4102975	0.5367152	
NFS1	Mitochondria	-0.0447982	0.8239805	0.9476434	-0.0825186	0.8218312		-0.0476507	0.8677949		0.0514224	0.8295888	0.9998749	0.1535124	0.5372575	
NFU1	Mitochondria	-0.0077914	0.9768986		0.0877269	0.85556		-0.0949342	0.8017793		0.1167081	0.7056182	0.9998749	-0.0072192	0.9834004	
NGRN	Mitochondria	0.433284	0.0052069	0.0470952	0.6060616	0.034639	0.3737369	0.3169386	0.1194409	0.4846168	0.173696	0.3293324	0.9998749	-0.1504946	0.4596891	
NIF3L1	Mitochondria	-0.0658116	0.8514231		0.1404433	0.8268662		-0.2085132	0.6625828		0.2047208	0.6108051	0.9998749	-0.1564595	0.7216348	
NIPSNAP1	Mitochondria	-0.0699173	0.4343923	0.7499764	-0.2455464	0.1520006	0.7094408	0.0208274	0.8676493	0.9673117	-0.1025209	0.3258398	0.9998749	0.08475	0.466168	0.8514041
NIPSNAP2	Mitochondria	0.1421651	0.6455253		0.3764748	0.4721891		-0.0331696	0.9392967		0.4741386	0.1720589	0.9998749	0.317307	0.4213255	
NIPSNAP3A	Mitochondria	0.2157676	0.3174121	0.6996021	0.1427392	0.7190712		0.2226741	0.4587511		-0.1271833	0.6413068	0.9998749	0.1187901	0.6645965	
NIPSNAP3B	Mitochondria	0.0548069	0.9121758		0.1921442	0.8293168		-0.0234964	0.9722451		0.2407959	0.6768193	0.9998749	0.0708447	0.9116823	
NIT1	Mitochondria	-0.1784239	0.4532267		-0.2556407	0.5593242		-0.1385029	0.6722123		-0.0955752	0.7431723	0.9998749	0.1037025	0.7201796	
NIT2	Mitochondria	-0.1750014	0.1911589	0.5363069	-0.2243567	0.4021138	0.9464092	-0.1538205	0.4092759	0.7656195	-0.1398269	0.3962745	0.9998749	-0.0577279	0.731924	0.9482562
NLN	Mitochondria	-0.2820699	0.0366891	0.2003026	-0.2963649	0.2554578	0.9187517	-0.2834229	0.1411619	0.5098188	-0.000265	0.9987821	0.9998749	0.0658023	0.6902081	0.9365555
NLRX1	Mitochondria	0.1357614	0.8145558		0.0298004	0.9789994		0.1961003	0.7957358		-0.1538512	0.8203368	0.9998749	-0.1324432	0.862543	
NME3	Mitochondria	0.3891925	0.40477		0.5984857	0.4595063		0.2579476	0.6888783		0.1990887	0.694854	0.9998749	0.2239976	0.713019	
NME4	Mitochondria	0.0817565	0.6363102	0.8971453	-0.0374755	0.9128195		0.1363084	0.5451755	0.845962	-0.0474664	0.8532233	0.9998749	-0.288842	0.2167441	
NME6	Mitochondria	0.1480085	0.7906133		0.1751922	0.8665829		0.125871	0.8664851		-0.1868297	0.7736388	0.9998749	0.0567585	0.9388267	
NMNAT3	Mitochondria	0.2420242	0.7333009		0.0298004	0.9789994		0.3890973	0.7200338		0.2033205	0.7758816	0.9998749	0.4620612	0.613485	

NNT	Mitochondria	-0.1519027	0.1271939	0.4250269	-0.1323996	0.5269189	0.9854749	-0.1745495	0.2036615	0.5968843	-0.1527719	0.2899222	0.9998749	-0.139363	0.2623655	0.6629064
NOA1	Mitochondria	0.0507292	0.8592499		-0.0056793	0.9911624		0.0694638	0.861993		0.3491113	0.2823464	0.9998749	0.1848777	0.611979	
NOCT	Mitochondria	0.4669508	0.2060763		1.004068	0.1660168		0.1781698	0.7057503		0.0225642	0.9557824	0.9998749	-0.7333686	0.1586891	
NRDC	Mitochondria	0.1296623	0.3388602	0.713827	0.2130999	0.3405713	0.9358893	0.0673668	0.7227267	0.9159826	-0.0502509	0.814518	0.9998749	-0.1770875	0.3095223	0.7028402
NSUN2	Mitochondria	0.1691912	0.2814871	0.6615935	0.3339432	0.270902	0.9358893	0.0631829	0.7692366	0.9311141	0.0049983	0.9782155	0.9998749	-0.2131128	0.303491	
NSUN3	Mitochondria	0.0591814	0.8113392		0.2111982	0.6367706		-0.0497154	0.8851565		0.2301917	0.4244305	0.9998749	0.0312052	0.9210714	
NSUN4	Mitochondria	0.4721275	0.1409146		0.4610819	0.4254839		0.4641755	0.2792896		0.1362851	0.6936738	0.9998749	0.0487582	0.9096587	
NT5DC2	Mitochondria	-0.1975551	0.7092332		-0.0626285	0.9437744		-0.3121808	0.6771192		0.4194788	0.5101503	0.9998749	0.2748194	0.6719134	
NT5DC3	Mitochondria	-0.1863909	0.484663		-0.2667479	0.6182265		-0.1480249	0.6693844		-0.4611853	0.1646066	0.9998749	-0.4545224	0.200808	
NTHL1	Mitochondria	-0.183301	0.7124201		-0.039641	0.9639566		-0.2694517	0.6942132		0.0745186	0.8980152	0.9998749	0.116003	0.8489765	
NUBPL	Mitochondria	-0.0638236	0.8314295		0.0981552	0.8559294		-0.1849973	0.6415446		0.2963406	0.4157137	0.9998749	-0.1421287	0.6979911	
NUDT13	Peroxisome	-0.1405728	0.7381809		0.0365036	0.9628706		-0.2462989	0.6609957		-0.1444806	0.7724234	0.9998749	-0.2648789	0.6135863	
NUDT19	Peroxisome	-0.0472727	0.8863924		-0.0277938	0.9647637		-0.072052	0.8692891		-0.0816845	0.8365207	0.9998749	-0.2619003	0.5370346	
NUDT2	Mitochondria	0.0133436	0.9751342		-0.1777651	0.8081196		0.1364296	0.8229024		0.2481136	0.6062998	0.9998749	0.5274917	0.324032	
NUDT5	Mitochondria	-0.1378836	0.4379296	0.7513739	-0.0809937	0.8137445		-0.1929058	0.4316797	0.7881107	-0.1862217	0.3928466	0.9998749	-0.1906017	0.3875736	
NUDT6	Mitochondria	-0.326123	0.2119562		-0.1318532	0.7919678		-0.442462	0.2064394		-0.1397099	0.6630247	0.9998749	-0.3712965	0.2503552	
NUDT8	Mitochondria	0.2141046	0.9059738													
NUDT9	Mitochondria	-0.1394945	0.3668947	0.7245031	-0.2073408	0.4893976	0.9835019	-0.1181031	0.5792393	0.8558909	-0.118346	0.5497523	0.9998749	-0.1483812	0.4422688	

OAT	Mitochondria	-0.9053375	4.289E-05	0.0011302	-0.7518531	0.0362397	0.3770636	-1.0026074	0.0002192	0.006383	-0.5055457	0.1590741	0.9998749	-0.3929411	0.2508515	
OCIAD1	Mitochondria	0.0244299	0.8176962	0.9456563	0.1043186	0.6142623	0.9854749	-0.0360566	0.8094474	0.9493368	-0.1389757	0.3028654	0.9998749	-0.2645446	0.0449094	0.2978001
OCIAD2	Mitochondria	-0.2839637	0.1646074	0.499869	-0.2822494	0.4727379		-0.2982673	0.2823613		-0.029119	0.9073721	0.9998749	-0.1710833	0.4896473	
OGDH	Mitochondria	-0.0045803	0.9820669	0.9918134	-0.0474716	0.8918205		-0.0082076	0.9781929		0.4198125	0.0951378	0.9998749	0.4041575	0.1137564	
OGDHL	Mitochondria	-0.1375493	0.4028604	0.7366398	-0.0931926	0.7478039	0.9854749	-0.1563917	0.4958459	0.8181458	0.0549903	0.7741398	0.9998749	0.0551985	0.8185959	
OGG1	Mitochondria	0.0126819	0.9790241		0.0668376	0.9407094		-0.0261105	0.9671908		0.1056333	0.8503264	0.9998749	-0.2413509	0.6997708	
OMA1	Mitochondria	-0.1516619	0.3837467	0.731203	0.0595014	0.855205		-0.2904456	0.2314037	0.620897	0.136859	0.5052049	0.9998749	-0.1808525	0.4032579	
OPA1	Mitochondria	-0.1348901	0.5731432		0.0466323	0.9092841		-0.2896268	0.4060283		0.4078598	0.155058	0.9998749	0.2062433	0.4833113	
OPA3	Mitochondria	0.3438976	0.0745426	0.309199	0.3189153	0.3637549		0.3547338	0.1851497	0.5692492	0.049573	0.8231718	0.9998749	0.0575534	0.81846	
OSBPL1A	Mitochondria	0.3471277	0.0821361	0.3296323	0.1892215	0.5548379	0.9854749	0.4635127	0.109486	0.4554249	0.4633234	0.0586094	0.9998749	0.6081226	0.0236966	
OSGEPL1	Mitochondria	0.1508072	0.6430596		0.2240956	0.7178379		0.0967316	0.820319		-0.1404424	0.7163523	0.9998749	-0.265138	0.5321289	
OTC	Mitochondria	-0.1440828	0.2410345	0.6137266	-0.0327295	0.8724748	0.9854749	-0.2223069	0.1464619	0.5137836	0.1401687	0.5926614	0.9998749	0.1097329	0.5246214	0.890164
OXA1L	Mitochondria	-0.1038115	0.3926316	0.73132	-0.0624055	0.7878572	0.9854749	-0.13679	0.4424417	0.7906449	0.1867767	0.1941235	0.9998749	0.1206363	0.4167485	0.7885242
OXCT1	Mitochondria	0.6948725	0.3662581		0.5852544	0.6042633		0.7551182	0.4772886		0.6275355	0.3602929	0.9998749	0.6605461	0.5147553	
OXLD1	Mitochondria	0.620556	0.1287699		0.5073586	0.4613241		0.6844952	0.2383492		0.3957266	0.3486642	0.9998749	0.6110671	0.2681001	
OXNAD1	Mitochondria	-0.0690468	0.8264423		-0.0036314	0.9952054		-0.1025946	0.8063177		0.001521	0.9966728	0.9998749	-0.2584131	0.5198827	
OXR1	Mitochondria	-0.1722831	0.3934164	0.73132	0.0191512	0.9558396		-0.3287869	0.2556258		0.5021073	0.0518771	0.9998749	0.1833043	0.4477106	
OXSM	Mitochondria	0.2597769	0.4609142		0.4443944	0.4934232		0.1259765	0.7929876		-0.0508132	0.897529	0.9998749	-0.0820656	0.8600653	

PAICS	Mitochondria	-0.1464146	0.2438167	0.618213	-0.236765	0.3274863	0.9358893	-0.121257	0.5033177	0.8234714	-0.1366938	0.418742	0.9998749	0.0620151	0.6747975	0.9365555
PAM16	Mitochondria	0.275428	0.6238104		0.5899794	0.5628993		0.1050761	0.8886486		0.0771925	0.9022907	0.9998749	-0.1315849	0.8633249	
PANK2	Mitochondria	0.1582814	0.5459033		0.1462051	0.7624784		0.1534338	0.6656026		0.0384685	0.8982737	0.9998749	-0.0509618	0.8801812	
PARK7	Mitochondria	-0.1107468	0.3288932	0.713693	-0.0765103	0.7174976	0.9854749	-0.1494938	0.3366801	0.7027967	-0.0745959	0.6401396	0.9998749	-0.1913357	0.1942483	0.5493259
PARL	Mitochondria	0.0747258	0.7783643		0.1102452	0.8197108		0.0471222	0.8980539		0.2916298	0.3347301	0.9998749	0.0704902	0.8358275	
PARS2	Mitochondria	0.0608389	0.8997819		0.3070203	0.7227834		-0.1197128	0.8563195		0.1100865	0.8452534	0.9998749	-0.1531932	0.8086988	
PC	Mitochondria	-0.0684505	0.680209	0.9193565	-0.2870571	0.3330043	0.9358893	0.0661154	0.7807587	0.9357761	-0.158691	0.4460133	0.9998749	0.3940046	0.0579365	
PCBD2	Mitochondria	-0.3260617	0.2696373		-0.1922476	0.7266349		-0.4155818	0.2995973		-0.0762708	0.8348696	0.9998749	-0.1886827	0.6003635	
PCCA	Mitochondria	-0.1649149	0.4296535	0.7496293	-0.1063386	0.7782266		-0.2141297	0.4633825		0.0533474	0.8281552	0.9998749	-0.0676184	0.7907084	
PCCB	Mitochondria	-0.0956993	0.4373327	0.7513739	-0.1120664	0.6465582	0.9854749	-0.0961454	0.5833121	0.8567937	-0.0507947	0.7314376	0.9998749	-0.040224	0.79156	0.9591016
PCK2	Mitochondria	-0.4157336	0.0018889	0.0238476	-0.707828	0.000148	0.0075151	-0.2525046	0.1376097	0.5045688	-0.1810733	0.4123949	0.9998749	0.1122472	0.6351063	0.9365555
PDE12	Mitochondria	-0.0240069	0.9281989		0.0246784	0.9586067		-0.0875461	0.8146366		0.2682883	0.3727759	0.9998749	0.1749566	0.5945576	
PDE2A	Mitochondria	0.1881878	0.6512455		0.2420343	0.7391223		0.1238481	0.8305097		0.2354176	0.6135616	0.9998749	0.111891	0.836902	
PDF	Mitochondria	-0.6182181	0.2953437		-1.1746553	0.2835182		-0.3327434	0.6686326		0.2680788	0.6790286	0.9998749	0.1780895	0.7820689	
PDHA1	Mitochondria	0.1801795	0.1156965	0.4100122	0.163822	0.4621178	0.966675	0.1847152	0.2715269	0.6493035	0.0781339	0.5902284	0.9998749	0.0669783	0.6717395	0.9365555
PDHB	Mitochondria	0.0177444	0.9186696	0.9715772	0.1389207	0.6665947	0.9854749	-0.0823117	0.7340564	0.922111	0.0651503	0.7671928	0.9998749	0.0164835	0.9403772	
PDHX	Mitochondria	0.1879789	0.3053732	0.6869339	0.2476923	0.4621189		0.1454433	0.5701933	0.8510009	0.0546118	0.7933821	0.9998749	-0.0592318	0.807105	
PDK1	Mitochondria	-0.0604786	0.8768776		0.3720651	0.5464235		-0.4342476	0.4545294		0.9383992	0.0368097	0.9998749	0.2747894	0.5653621	

PDK2	Mitochondria	-0.2242618	0.2548677	0.6304075	-0.2729756	0.4428534		-0.2053771	0.4654151		0.0321868	0.8979531	0.9998749	0.1793194	0.4617489	
PDK3	Mitochondria	-0.3273813	0.1202822	0.4141537	-0.2275119	0.5522255		-0.4003463	0.1617441		0.0776808	0.7674671	0.9998749	-0.050453	0.8428683	
PDK4	Mitochondria	-0.273603	0.0529883	0.2528419	0.0768101	0.6334471	0.9854749	-0.4930619	0.0070601	0.0919675	0.2143006	0.4064296	0.9998749	-0.3594117	0.0086175	0.1030187
PDP1	Mitochondria	-0.0401828	0.9401518		0.5373877	0.5427748		-0.4776674	0.5327821		1.0131976	0.0892564	0.9998749	0.2752205	0.6818038	
PDP2	Mitochondria	0.0038897	0.9918107		-0.0281651	0.9666718		0.0065036	0.9898792		0.2802863	0.5233169	0.9998749	0.141008	0.7650173	
PDPR	Mitochondria	0.2124371	0.3782021		0.2564417	0.5418618		0.1716988	0.613281		0.2712324	0.3382391	0.9998749	0.1927045	0.5415514	
PDSS1	Mitochondria	0.166145	0.7616716		0.4493494	0.6664968		-0.0004335	0.9995228		0.2117296	0.7320103	0.9998749	-0.3699919	0.6168172	
PDSS2	Mitochondria	-0.2160638	0.1441119	0.4720639	-0.2640169	0.3456616	0.9358893	-0.1905906	0.3661912	0.7247624	0.0367907	0.8365214	0.9998749	0.0953032	0.5941307	
PET100	Mitochondria	0.0568788	0.7312506	0.9456563	-0.1473917	0.6549609		0.1598406	0.4694187	0.798895	0.2062391	0.4467809	0.9998749	-0.1366468	0.5115078	
PET117	Mitochondria	0.2955324	0.3849188		0.134768	0.8172162		0.3937307	0.4133693		0.1110233	0.7702689	0.9998749	0.4099917	0.3605056	
PEX11B	Peroxisome	0.0621093	0.7876395		-0.064686	0.8789354		0.1217824	0.7020304		-0.1676875	0.5286792	0.9998749	0.0886859	0.7608565	
PGAM5	Mitochondria	0.1652914	0.6073001		0.1570084	0.7870794		0.1474401	0.7408448		0.1162634	0.7483655	0.9998749	0.1203487	0.7750079	
PGS1	Mitochondria	0.1791128	0.7397747		0.0508853	0.9603329		0.2465684	0.7274839		0.028943	0.9614805	0.9998749	-0.0699812	0.9224662	
PHB	Mitochondria	0.3209891	0.0281053	0.1720387	0.139732	0.5775748	0.9854749	0.4412441	0.0400552	0.2802461	0.1050224	0.5055431	0.9998749	0.4304092	0.053682	
PHB2	Mitochondria	-0.0343153	0.7435428	0.9456563	-0.0634892	0.7374916	0.9854749	-0.0230043	0.8826015	0.9722587	0.0770804	0.5538528	0.9998749	0.0378933	0.8402127	0.9660934
PHYH	Peroxisome	-0.5046428	1.083E-09	9.375E-08	-0.6282892	0.0001253	0.0073418	-0.446381	0.0002678	0.0067904	-0.0425585	0.7864962	0.9998749	0.2163628	0.1350713	0.4871108
PICK1	Mitochondria	0.4165057	0.3488672		0.4845765	0.5119733		0.3624469	0.5665977		0.4867542	0.3168013	0.9998749	0.4217622	0.4745882	
PIGBOS1	Mitochondria	0.1540298	0.6874549		0.2338785	0.7425931		0.1010592	0.8428067		0.113285	0.7956134	0.9998749	-0.1768939	0.7235577	
PINK1	Mitochondria	2.772E-05	0.9997657	0.9997657	-0.0416158	0.8238324	0.9854749	0.0210921	0.8841637	0.9722587	0.1436371	0.223212	0.9998749	0.221744	0.1250792	0.4871108

PISD	Mitochondria	0.24332	0.5061077		0.2072768	0.7558668		0.2453276	0.6195695		0.1101993	0.7894405	0.9998749	-0.0096755	0.9840198	
PITRM1	Mitochondria	0.1859675	0.5312058		0.3232953	0.5430797		0.0842795	0.8382945		0.3152799	0.3220851	0.9998749	0.0769397	0.8410968	
PLD6	Mitochondria	-0.1880546	0.6008584		-0.5540048	0.421199		-0.0216086	0.9628516		-0.6163131	0.1902439	0.9998749	-0.1191036	0.785313	
PLGRKT	Mitochondria	0.2645889	0.4285033		0.335978	0.5813958		0.2203507	0.6305153		0.1196724	0.7418411	0.9998749	-0.1242825	0.7821482	
PLPBP	Mitochondria	-0.1851223	0.3060596	0.6869339	0.0029568	0.9932893		-0.296165	0.2212113	0.6138361	-0.0331198	0.8813266	0.9998749	-0.3911173	0.0873604	
PLSCR3	Mitochondria	1.0076941	0.7304931													
PMPCA	Mitochondria	0.052818	0.7777661	0.9456563	-0.1686012	0.594105	0.9854749	0.1812504	0.5080805	0.8234714	0.0551391	0.8006863	0.9998749	0.4562134	0.0454771	
PMPCB	Mitochondria	-0.0947563	0.3670337	0.7245031	0.0080871	0.9687011	0.9970186	-0.1796202	0.2455584	0.6298	0.0902499	0.4948214	0.9998749	-0.0233894	0.8551593	0.9660934
PNKD	Mitochondria	0.6607016	2.464E-05	0.0007111	0.3603063	0.2361757	0.8723607	0.8031713	8.584E-05	0.0032686	-0.3825564	0.1001956	0.9998749	0.0119214	0.958891	
PNPLA8	Mitochondria	0.0736727	0.7475813	0.9456563	0.0858815	0.8432527		0.058033	0.8489201		0.0181783	0.946545	0.9998749	-0.2689549	0.3786585	
PNPO	Mitochondria	0.033864	0.7132627	0.9355783	-0.075977	0.6822208	0.9854749	0.0792817	0.5607026	0.8510009	-0.0444035	0.6962881	0.9998749	0.0994128	0.3891385	0.7798739
PNPT1	Mitochondria	0.4262826	0.03228	0.1863016	0.4852894	0.1860707		0.3667966	0.1851208		0.1272291	0.5654764	0.9998749	0.0166011	0.9502835	
POLB	Mitochondria	0.329839	0.2222591		0.6036841	0.2277402		0.1473555	0.6890501		0.0012501	0.9967013	0.9998749	-0.3149664	0.3914893	
POLDIP2	Mitochondria	-0.0349792	0.7837476	0.9456563	-0.177654	0.4294875	0.9506771	0.0442165	0.8155365	0.9493368	0.033006	0.8307778	0.9998749	0.351256	0.0215156	0.1886197
POLG	Mitochondria	0.1785718	0.5132993		0.0767062	0.8699472		0.2500177	0.5240509		0.3092502	0.3190939	0.9998749	0.4559037	0.1956232	
POLG2	Mitochondria	-0.0970313	0.8066608		0.3057298	0.6562029		-0.3880829	0.4856562		0.3212405	0.486945	0.9998749	-0.0871784	0.8596306	
POLQ	Mitochondria	1.2380913	0.1289467		1.0338887	0.3656836		1.3134023	0.3116147		0.2363178	0.7287153	0.9998749			
POLRMT	Mitochondria	0.1571257	0.5253389		-0.1413088	0.7480842		0.3529872	0.3137093		0.0682245	0.805916	0.9998749	0.4912138	0.1236579	
PPA2	Mitochondria	-0.1209455	0.3597727	0.7245031	-0.0532583	0.8328448	0.9854749	-0.1840923	0.3377887	0.7027967	0.1475792	0.3616396	0.9998749	-0.0128405	0.9382713	0.9660934
PPIF	Mitochondria	-0.0650859	0.6118775	0.8849588	-0.1564904	0.4914636	0.9835019	-0.0148278	0.9363804	0.9778656	0.0493791	0.7931032	0.9998749	0.1342495	0.5659904	0.9132237

PPM1K	Mitochondria	0.3654456	0.0352902	0.1995351	0.5851081	0.0528453	0.4451681	0.2256841	0.3005209	0.6864316	0.0036528	0.989763	0.9998749	-0.3541802	0.1060489	
PPOX	Mitochondria	0.6449803	0.2574377		0.5124986	0.6102503		0.7354659	0.3370405		0.0791766	0.9005028	0.9998749	0.1603635	0.8412163	
PPTC7	Mitochondria	-0.1242642	0.6638071		0.1694792	0.7421307		-0.3303707	0.4066529		0.1823208	0.5867726	0.9998749	-0.2237535	0.5315988	
PRDX2	Mitochondria	0.1990927	0.1472688	0.4747337	-0.0426469	0.8639817	0.9854749	0.3468509	0.0752772	0.3704584	-0.1235812	0.4119654	0.9998749	0.0754288	0.700092	0.9386991
PRDX3	Mitochondria	-0.3036773	0.0075617	0.0643015	-0.2122072	0.3249159	0.9358893	-0.386687	0.0197022	0.1840115	-0.3556199	0.046	0.9998749	-0.3979746	0.0070764	0.0930543
PRDX4	Mitochondria	-0.2130481	0.0903079	0.3576902	-0.2546861	0.2897187	0.9358893	-0.22058	0.2264086	0.6138361	-0.0144028	0.9445626	0.9998749	-0.1388925	0.3798473	0.7798739
PRDX5	Peroxisome	-0.0606477	0.6770729	0.9184946	-0.1375416	0.6013047	0.9854749	-0.0339203	0.8727961	0.9698515	0.0990584	0.5742313	0.9998749	0.1142057	0.546983	
PRDX6	Mitochondria	-0.2487669	0.0022948	0.0262387	-0.1733483	0.2586183	0.9220303	-0.3048653	0.0126291	0.1369004	-0.3805304	0.0087646	0.9998749	-0.4548898	0.0001819	0.0060905
PRELID1	Mitochondria	0.1540186	0.3369264	0.713827	-0.0448841	0.8786203	0.9854749	0.2746453	0.2269333	0.6138361	0.3331133	0.1409793	0.9998749	0.3585856	0.0802109	
PRELID2	Mitochondria	0.8326794	0.1500951		0.6741888	0.5004299		0.9355235	0.2338781		0.2590763	0.6555372	0.9998749	0.3220124	0.6956112	
PRELID3B	Mitochondria	0.2183682	0.3305709	0.713693	0.5275561	0.2067866		0.0094299	0.9753961		0.0583663	0.8221549	0.9998749	-0.2772559	0.3429202	
PREPL	Mitochondria	-0.2896066	0.2452655		-0.1361767	0.7512566		-0.398863	0.2620587		0.2633407	0.378842	0.9998749	0.1980482	0.5324054	
PRIMPOL	Mitochondria	0.2027234	0.7511877		0.4566785	0.6965027		0.0526966	0.951051		-0.0536702	0.9385632	0.9998749	-0.4770877	0.5849084	
PRKACA	Mitochondria	0.142344	0.2984329	0.6841928	0.0241639	0.9270784	0.9909626	0.20092	0.3009205	0.6864316	-0.0212525	0.9049602	0.9998749	0.1466886	0.4103098	
PRKN	Mitochondria	0.4503781	0.4410891		0.1448719	0.8768606		0.6783277	0.4348211		0.3094334	0.6209379	0.9998749	0.8685257	0.2694595	
PRODH	Mitochondria	0.3738485	0.3300063		0.1361668	0.870291		0.5362265	0.2384545		-1.2161104	0.0397594	0.9998749	-0.59621	0.3729865	
PRODH2	Mitochondria	-0.0535538	0.7920155	0.9456563	-0.4632695	0.2189968		0.1844572	0.5039824	0.8234714	-0.4879287	0.0436333	0.9998749	0.1789365	0.5092006	
PRXL2A	Mitochondria	-0.3195458	0.0051499	0.0470952	-0.1842595	0.3821627	0.9464092	-0.4283446	0.0076661	0.0962193	0.0646263	0.7935642	0.9998749	-0.0738079	0.6824669	0.9365555
PTCD1	Mitochondria	0.6972465	0.187492		1.1104886	0.2304143		0.4115884	0.5690514		0.3779822	0.4954107	0.9998749	0.0684608	0.9262327	

PTCD2	Mitochondria	-0.2222686	0.6296813		0.0946132	0.9097925		-0.4412785	0.4833719		0.0133136	0.9811363	0.9998749	-0.2133671	0.711719	
PTCD3	Mitochondria	-0.0495886	0.6709369	0.9184946	0.0926364	0.6842231	0.9854749	-0.1473222	0.3748884	0.7334772	0.1266574	0.3899631	0.9998749	-0.086744	0.5731544	0.9135733
PTGES2	Mitochondria	0.4135176	0.1830116		0.3352199	0.5472417		0.454797	0.291666		0.1844521	0.5784978	0.9998749	0.1966464	0.6366286	
PTPMT1	Mitochondria	-0.0391212	0.8574266	0.9633793	-0.1383818	0.7335955		-0.0052156	0.986187		0.0139652	0.9564443	0.9998749	0.0697715	0.7981745	
PTRH2	Mitochondria	0.3163622	0.2728346		0.2289632	0.6617626		0.3484431	0.3841653		0.0070566	0.9820318	0.9998749	0.1105627	0.7730891	
PUS1	Mitochondria	0.5047096	0.2908203		0.306281	0.7132206		0.6409428	0.3313256		0.1743406	0.7282904	0.9998749	0.4063498	0.5251443	
PUSL1	Mitochondria	0.4304808	0.8588194								0.3957898	0.6494647	0.9998749			
PXMP2	Peroxisome	-0.2524632	0.1029709	0.3804901	-0.4386392	0.1405279	0.7094408	-0.1654483	0.4041923	0.7639967	-0.2800696	0.2024908	0.9998749	-0.1762824	0.4087761	0.7885242
PXMP4	Peroxisome	0.3639539	0.1276483	0.4250269	0.3094947	0.4618245		0.3822973	0.2416172		0.258977	0.3663283	0.9998749	0.1270964	0.67851	
PYCR2	Mitochondria	0.3607281	0.1749764		0.3220911	0.4929301		0.3777625	0.3082556		0.1777296	0.5508273	0.9998749	0.2094254	0.5504323	
PYURF	Mitochondria	0.0366794	0.8629818	0.9644421	0.0641547	0.8690836		-0.0036282	0.99033		0.0485288	0.8432835	0.9998749	0.0192136	0.9431679	
QDPR	Mitochondria	-0.3299286	0.0071335	0.0626507	-0.1757593	0.4610293	0.966675	-0.4332641	0.0127221	0.1369004	0.0035145	0.9821354	0.9998749	-0.3312586	0.0419266	0.2978001
QRSL1	Mitochondria	0.2014687	0.3951866		0.2286626	0.597726		0.1649832	0.6173511		0.1657504	0.5364332	0.9998749	0.0512412	0.8679947	
QTRT1	Mitochondria	0.2263139	0.4496238		0.0931488	0.8645084		0.3074118	0.4537995		-0.133328	0.6987539	0.9998749	0.2718907	0.4831598	
RAB24	Mitochondria	0.4925949	0.1902223		0.3128928	0.6269317		0.6231643	0.2356692		0.0086598	0.9827596	0.9998749	0.3701905	0.4655152	
RAB5IF	Mitochondria	0.4272277	0.1391521		0.4445325	0.387062		0.3863947	0.3390031		0.1449842	0.6390627	0.9998749	0.1606266	0.6735093	
RARS2	Mitochondria	-0.4342931	0.1104064		-0.2411709	0.6021947		-0.58419	0.1384797		0.3502207	0.2855605	0.9998749	0.0717133	0.8274105	
RBFA	Mitochondria	0.139516	0.4440023	0.7536846	-0.0678007	0.8451692		0.2611902	0.2972703	0.684413	-0.1057901	0.6164398	0.9998749	0.0566373	0.812967	
RCC1L	Mitochondria	0.037949	0.8783695		-0.1859272	0.6745554		0.1713313	0.6237245		-0.0224754	0.9377874	0.9998749	0.3086806	0.3264971	
RDH13	Mitochondria	0.5099193	0.3512766		0.4525154	0.6370362		0.5639284	0.4489366		0.0110412	0.9852916	0.9998749	0.1960269	0.7939651	

RDH14	Mitochondria	0.0602463	0.7418227	0.9456563	0.1479829	0.6637839		-0.0080661	0.9748012	0.9876706	0.0515528	0.8037903	0.9998749	-0.1205605	0.6080165	
RECQL4	Mitochondria	2.0034442	0.0089647		1.4799488	0.2096516		2.2789922	0.0377471		-0.0903555	0.8896163	0.9998749			
REXO2	Mitochondria	0.2885537	0.0721166	0.307765	0.2457785	0.4131884	0.9464092	0.3060398	0.1710556	0.5570559	0.1421095	0.4261929	0.9998749	0.0512086	0.8086803	
RFK	Mitochondria	0.152495	0.3972803	0.7362442	0.2341352	0.4978098		0.0851828	0.7319123	0.922111	0.0045963	0.9824004	0.9998749	-0.1679038	0.4806609	
RHOT1	Mitochondria	0.0095403	0.9785978		0.0579573	0.926055		-0.0381239	0.9383059		0.159479	0.7022846	0.9998749	0.078912	0.8599353	
RHOT2	Mitochondria	0.2195955	0.6351798		0.3495498	0.6567799		0.1502044	0.8180679		0.4890301	0.3260527	0.9998749	0.2134594	0.7222675	
RIDA	Mitochondria	-0.3918797	0.0008913	0.0136014	-0.4643528	0.0195073	0.3199189	-0.3635858	0.0099855	0.1123366	-0.4553982	0.0404976	0.9998749	-0.5365721	8.844E-05	0.0060905
RMDN1	Mitochondria	-0.1679929	0.2933937	0.6760327	-0.0230894	0.9414821	0.9909626	-0.2542326	0.2453762	0.6298	0.010269	0.9574296	0.9998749	-0.2883858	0.157946	
RMDN3	Mitochondria	-0.008722	0.9700142		-0.169677	0.691402		0.0814399	0.7980011		-0.1175013	0.6612645	0.9998749	0.0522005	0.8570807	
RMND1	Mitochondria	-0.1092831	0.5515069	0.8415686	-0.0266939	0.9378409		-0.1964848	0.4487528	0.7923079	0.0545701	0.8107344	0.9998749	-0.0633232	0.7800437	
RNASEH1	Mitochondria	0.1056629	0.6859898		0.2074311	0.6728372		0.0397193	0.910834		-0.0499383	0.8688071	0.9998749	-0.1979237	0.5651952	
ROMO1	Mitochondria	0.1535952	0.5209827		-0.1450558	0.7536329		0.3165331	0.3119411		0.1940543	0.5438289	0.9998749	0.011778	0.9688375	
RPIA	Mitochondria	0.2888646	0.4981737		0.4524312	0.5490752		0.1718937	0.768997		0.3714228	0.4249385	0.9998749	-0.0270469	0.9622007	
RPUSD3	Mitochondria	0.0133052	0.9804048		-0.1919108	0.84589		0.1186601	0.8711944		-0.0184494	0.9764226	0.9998749	0.1126472	0.8709763	
RPUSD4	Mitochondria	-0.033673	0.8999898		-0.0045677	0.9926166		-0.0651727	0.858656		0.0801354	0.8002618	0.9998749	-0.0078231	0.9813864	
RSAD1	Mitochondria	0.0983316	0.5692486	0.860261	0.2243665	0.4900322	0.9835019	0.0169608	0.9421879	0.981859	-0.0384641	0.8615503	0.9998749	-0.3036329	0.1647137	
RTL10	Mitochondria	0.0737047	0.7868858		0.1504451	0.7618156		0.0128312	0.9727409		0.2105032	0.5083662	0.9998749	0.0094487	0.9784225	
RTN4IP1	Mitochondria	0.1227349	0.7519374		0.12335	0.8550107		0.1041651	0.848921		0.2339358	0.5911274	0.9998749	0.2596861	0.5968317	
SAMM50	Mitochondria	0.0130271	0.9470122	0.9826873	0.1111711	0.7589354		-0.0712532	0.7940539		0.0941404	0.6810565	0.9998749	0.0291566	0.9055637	

SARDH	Mitochondria	-0.2335563	0.2106531	0.5648486	-0.6148033	0.0629291	0.469108	-0.0076611	0.9756988	0.9876706	-0.1879629	0.4323012	0.9998749	0.2357988	0.3127233	
SARS2	Mitochondria	0.2346147	0.6413433		0.0506004	0.9584875		0.3372225	0.6116898		0.0029345	0.9958155	0.9998749	0.0252355	0.9697188	
SCO1	Mitochondria	0.02928	0.8515929	0.9633793	0.0933855	0.7454389	0.9854749	-0.0474022	0.8336956	0.9517856	0.0338599	0.8827349	0.9998749	-0.0218941	0.9120666	
SCP2	Peroxisome	-0.2417733	0.0296873	0.1799052	-0.1561107	0.3902381	0.9464092	-0.3080712	0.0384725	0.2800571	0.0102821	0.9604997	0.9998749	-0.0502463	0.7649612	0.9526674
SDHA	Mitochondria	-0.1229788	0.3128135	0.6943772	-0.2597417	0.1597453	0.7197318	-0.0489003	0.7706685	0.9311141	0.1310419	0.4626513	0.9998749	0.333534	0.1053542	0.4435874
SDHAF1	Mitochondria	0.0999851	0.7841823		-0.2025942	0.7591152		0.2775292	0.5777472		-0.1367628	0.7449265	0.9998749	0.338425	0.4643943	
SDHAF2	Mitochondria	0.2437572	0.2636326	0.6416118	0.2544477	0.5393004		0.2231682	0.4538217		-0.138157	0.5818573	0.9998749	-0.0943533	0.7437445	
SDHAF3	Mitochondria	-0.3221177	0.2915711		-0.2235199	0.6918934		-0.4059943	0.3336698		0.0216473	0.9525033	0.9998749	-0.1486507	0.6881895	
SDHAF4	Mitochondria	-0.0026481	0.9954819		0.132573	0.8703382		-0.0993551	0.8789968		0.3071066	0.5698195	0.9998749	0.0286667	0.9617159	
SDHB	Mitochondria	0.0697479	0.5049542	0.8052691	0.015284	0.9426229	0.9909626	0.0809008	0.5878703	0.8586913	0.0147576	0.9147017	0.9998749	0.0240978	0.8525997	0.9660934
SDHC	Mitochondria	0.020775	0.8940389	0.9646758	0.167841	0.5458223	0.9854749	-0.0935969	0.6604085	0.9036861	0.1264943	0.5333102	0.9998749	-0.0982032	0.5970488	
SDHD	Mitochondria	-0.2855295	0.0409736	0.2140518	-0.2632307	0.3160282	0.9358893	-0.3158991	0.1148069	0.4735784	-0.0844115	0.6236389	0.9998749	-0.0913959	0.5875146	0.9197401
SDR39U1	Mitochondria	0.059547	0.8759199		-0.105269	0.8752053		0.166413	0.7525536		0.0327325	0.9423321	0.9998749	0.3129181	0.5196544	
SDSL	Mitochondria	0.3017162	0.3370528		0.1739941	0.7519413		0.3972051	0.3697087		0.0690713	0.8377016	0.9998749	0.3480366	0.3981716	
SELENOO	Mitochondria	0.1970956	0.3667517	0.7245031	-0.165058	0.6733118		0.4265577	0.1581848		-0.2796728	0.2662807	0.9998749	0.3218812	0.273136	
SERAC1	Mitochondria	0.1043605	0.7589291		0.3388898	0.5795977		-0.0537718	0.9083082		0.3491346	0.35721	0.9998749	-0.069693	0.8749575	
SETD9	Mitochondria	0.0393621	0.9213968		0.3780885	0.6150731		-0.1616404	0.758761		0.0095285	0.9836018	0.9998749	-0.5242218	0.322626	
SFXN1	Mitochondria	-0.3121571	0.0750037	0.309199	-0.2060733	0.5207707		-0.3904607	0.1164847	0.4765282	0.1186164	0.5839779	0.9998749	0.015094	0.942438	

SFXN2	Mitochondria	-0.2300322	0.3533444		-0.2787224	0.5364508		-0.2131156	0.5280925		-0.0299452	0.9220167	0.9998749	0.0205247	0.9448434	
SFXN3	Mitochondria	0.6858853	0.1018448		0.7853768	0.2577578		0.5893914	0.3196639		0.48873	0.2468153	0.9998749	0.4243306	0.4645315	
SFXN4	Mitochondria	0.2641636	0.3957077		0.2820956	0.6274425		0.2444511	0.5524975		-0.0382905	0.9132656	0.9998749	-0.2302291	0.5808761	
SFXN5	Mitochondria	0.3674472	0.1561429		0.2852701	0.5475336		0.4036128	0.2551807		-0.1174635	0.6857879	0.9998749	0.0705322	0.836105	
SHMT2	Mitochondria	-0.0596548	0.7282593	0.9456563	0.0819751	0.8011792		-0.1463757	0.5350488	0.8434687	0.1482583	0.4819837	0.9998749	-0.1627865	0.4496401	
SIRT3	Mitochondria	-0.0734785	0.8006876		-0.0943083	0.8600722		-0.0667682	0.8662102		0.0389191	0.9120085	0.9998749	0.0422115	0.9072056	
SIRT4	Mitochondria	0.2663248	0.7485121					0.923472	0.4161651		-0.6364394	0.4321488	0.9998749	1.1253375	0.2129868	
SIRT5	Mitochondria	0.0065712	0.9697497	0.988921	0.143644	0.6681277		-0.0749555	0.7497391	0.922111	-0.0854568	0.6828337	0.9998749	-0.3428089	0.1225323	
SLC25A1	Mitochondria	0.0919767	0.6365882	0.8971453	-0.3344973	0.380231		0.3089704	0.2229044	0.6138361	-0.4616766	0.053479	0.9998749	0.06313	0.8041809	
SLC25A10	Mitochondria	0.0497776	0.8441719		-0.2021892	0.66427		0.2109226	0.5385945		-0.3307461	0.2473901	0.9998749	0.0977653	0.7688694	
SLC25A11	Mitochondria	0.2404853	0.3479062		0.1950692	0.6919336		0.2515993	0.4574379		0.1469883	0.6304147	0.9998749	-0.1522982	0.6509181	
SLC25A12	Mitochondria	0.9456545	0.2234559		1.3107423	0.2364109		0.6901277	0.5185937		0.7123221	0.2683738	0.9998749	0.4164113	0.7053704	
SLC25A13	Mitochondria	-0.3777753	0.0013871	0.0191039	-0.4315339	0.0524112	0.4451681	-0.364706	0.0347144	0.2727561	-0.129757	0.3847501	0.9998749	-0.0332819	0.8042788	0.9658691
SLC25A14	Mitochondria	0.2739773	0.6205445		0.4528619	0.6366391		0.1555249	0.8379096		0.0756405	0.9046159	0.9998749	0.0514275	0.9441723	
SLC25A15	Mitochondria	-0.0073387	0.9445762	0.9818408	0.0166725	0.9404101	0.9909626	-0.0341779	0.8142034	0.9493368	-0.4192314	0.0145793	0.9998749	-0.4931606	0.0020751	0.0363838
SLC25A16	Mitochondria	-0.0264075	0.9333699		-0.0423253	0.9396657		-0.024934	0.9544308		0.2033729	0.5837306	0.9998749	0.1353731	0.7302286	
SLC25A18	Mitochondria	0.5274052	0.0038128	0.0385088	0.1846803	0.5551728	0.9854749	0.7077431	0.0011335	0.0207802	-0.3549176	0.1774792	0.9998749	0.2480329	0.3508322	
SLC25A20	Mitochondria	0.0272053	0.8200295	0.9465483	-0.0033851	0.9887731	0.9970186	0.0354635	0.8329378	0.9517856	-0.0960843	0.553193	0.9998749	-0.0456906	0.7725367	0.9538834
SLC25A22	Mitochondria	0.2474336	0.4532548		-0.1293415	0.8217776		0.4942084	0.2864257		-0.013963	0.9701394	0.9998749	0.6131552	0.1480025	

SLC25A23	Mitochondria	0.0494974	0.7101609	0.9355783	0.0564762	0.8122887	0.9854749	0.0416571	0.824897	0.9517856	0.0888383	0.6348114	0.9998749	0.0729545	0.6376707	0.9365555
SLC25A24	Mitochondria	0.7481542	0.1040043		0.9632764	0.1769387		0.5521023	0.4096029		1.1236302	0.0163529	0.9998749	0.8186328	0.1956138	
SLC25A25	Mitochondria	0.2754067	0.2207994	0.5817585	0.5261355	0.1502734	0.7094408	0.1190436	0.6710794	0.9036861	-0.075479	0.8112527	0.9998749	-0.2916836	0.3436766	
SLC25A26	Mitochondria	0.0845533	0.7857158		0.2094833	0.7107582		-0.0072839	0.9863311		0.1770267	0.615519	0.9998749	-0.0611199	0.8787005	
SLC25A27	Mitochondria	-0.2477049	0.1785705	0.5153047	-0.0003799	0.9991226		-0.4020613	0.1049875	0.4490901	0.0260455	0.9193679	0.9998749	-0.1682907	0.4967895	
SLC25A28	Mitochondria	0.5861313	0.008218	0.0682205	0.4275202	0.3045122		0.6672709	0.0269152		-0.0311666	0.894968	0.9998749	0.1432377	0.6336723	
SLC25A29	Mitochondria	0.4178811	0.2223686		0.7377523	0.2365868		0.2025547	0.6610547		0.0963559	0.797982	0.9998749	-0.0370273	0.9353234	
SLC25A3	Mitochondria	-0.1261822	0.2481677	0.6214448	-0.122772	0.512858	0.9854749	-0.1595288	0.3425358	0.7027967	0.2146866	0.120705	0.9998749	0.2169354	0.0609036	0.3559478
SLC25A30	Mitochondria	0.0471941	0.809186	0.9456563	0.2645841	0.4388715		-0.1226076	0.662658		0.3177155	0.1697955	0.9998749	0.0570688	0.8168739	
SLC25A32	Mitochondria	-0.0529105	0.8685382		0.0124597	0.9830855		-0.1081575	0.8045503		0.0113384	0.9755687	0.9998749	-0.0777493	0.8486581	
SLC25A33	Mitochondria	0.797098	0.0064206		0.5874523	0.2935352		0.8879969	0.0219892		-0.0611059	0.8393919	0.9998749	-0.1001547	0.8105439	
SLC25A34	Mitochondria	0.0305108	0.9286358		-0.0901905	0.8864819		0.1082792	0.8125534		-0.1240136	0.7532752	0.9998749	0.0206687	0.9621878	
SLC25A35	Mitochondria	0.7807473	0.6777355								0.7681993	0.355632	0.9998749			
SLC25A36	Mitochondria	0.6246249	0.0793099		0.8757749	0.1449243		0.4638057	0.332393		0.4681937	0.2254891	0.9998749	0.4484126	0.3833023	
SLC25A37	Mitochondria	-0.3530834	0.0378577	0.2030242	-0.1958663	0.5178748	0.9854749	-0.460034	0.0511824	0.3127812	0.0604971	0.7895246	0.9998749	-0.1140221	0.5600274	
SLC25A38	Mitochondria	-0.1220156	0.408434	0.7366398	-0.2041747	0.4754766	0.9698776	-0.0775772	0.7071947	0.9139984	-0.2211185	0.2208221	0.9998749	-0.0630543	0.7412278	
SLC25A39	Mitochondria	0.1610976	0.5243235		0.0246082	0.9567329		0.2449692	0.4860919		0.2199939	0.4445191	0.9998749	0.2293228	0.4869038	
SLC25A4	Mitochondria	0.033658	0.8136749	0.9456563	-0.053873	0.8453351	0.9854749	0.0642066	0.7501215	0.922111	-0.141327	0.4093129	0.9998749	-0.0239445	0.8934135	
SLC25A40	Mitochondria	-0.0512886	0.8802132		0.1593109	0.7857408		-0.2189331	0.6519653		0.3255522	0.4154112	0.9998749	0.1278528	0.763038	

SLC25A42	Mitochondria	0.0358708	0.8397931	0.957979	-0.1383044	0.6817295		0.1334033	0.5818522	0.8567937	-0.2564867	0.2623452	0.9998749	-0.0310391	0.8992247	
SLC25A43	Mitochondria	0.2275654	0.3644834		0.418981	0.384242		0.0945581	0.7795147		-0.0168759	0.9529096	0.9998749	-0.3232888	0.332364	
SLC25A44	Mitochondria	0.1947329	0.3046677	0.6869339	0.0105776	0.9767863		0.2831728	0.2660643	0.6427056	-0.3074026	0.1597983	0.9998749	-0.0609893	0.8061104	
SLC25A45	Mitochondria	0.6523194	0.1515554		0.5115829	0.5064804		0.7617078	0.2294216		0.1082573	0.8184336	0.9998749	0.4071892	0.509963	
SLC25A46	Mitochondria	-0.1813297	0.196035	0.5429961	0.0497595	0.8494445	0.9854749	-0.3452154	0.0763474	0.3705093	0.0799736	0.6552859	0.9998749	-0.2182974	0.2201487	0.5908073
SLC25A47	Mitochondria	0.0940413	0.5853036	0.8642121	-0.1861696	0.5253756	0.9854749	0.2490945	0.2562982	0.6375258	-0.0038909	0.9850882	0.9998749	0.2772585	0.320495	
SLC25A5	Mitochondria	-0.0365424	0.763168	0.9456563	-0.0649421	0.7463858	0.9854749	-0.0349791	0.8325269	0.9517856	0.0818353	0.6561709	0.9998749	0.2103797	0.2354996	0.6193639
SLC25A51	Mitochondria	1.0340327	0.0981355		0.8388174	0.4329558		1.2028778	0.1591161		-0.1918384	0.7645962	0.9998749	0.4763148	0.6005135	
SLC25A53	Mitochondria	0.5865192	0.7826547								0.3651526	0.6766498	0.9998749			
SLC25A6	Mitochondria	0.1472793	0.4551741	0.7660516	0.1458181	0.6746295		0.1371233	0.6284328		0.3982095	0.0739965	0.9998749	0.3481898	0.1684686	
SLC30A9	Mitochondria	-0.0857229	0.5239922	0.820515	0.1637512	0.5040185	0.9854749	-0.2698004	0.1612627	0.5393582	0.2322753	0.176539	0.9998749	-0.0736063	0.6696951	0.9365555
SLC8B1	Mitochondria	0.195608	0.6709263		0.2641955	0.7399517		0.1502963	0.8151605		0.2468367	0.634568	0.9998749	0.2331887	0.6937961	
SLIRP	Mitochondria	0.0263333	0.8883759	0.9646758	-0.0324945	0.9276397		0.0472331	0.8532086	0.9576831	0.3234065	0.2576486	0.9998749	-0.1158968	0.6215194	
SMDT1	Mitochondria	-0.2751084	0.201061	0.5503258	-0.3161995	0.4433474		-0.2636802	0.366534		-0.0225585	0.9306208	0.9998749	-0.1419864	0.587694	
SMIM20	Mitochondria	0.0362147	0.8537873	0.9633793	0.1310218	0.7245111		-0.0407132	0.8806297		0.0346153	0.8799888	0.9998749	-0.1270491	0.6119363	
SMIM8	Mitochondria	-0.1877234	0.6236437		-0.3434234	0.6482684		-0.135965	0.7877129		-0.3128384	0.494165	0.9998749	-0.1600176	0.7364326	
SNAP29	Mitochondria	0.1002534	0.4192943	0.7471844	0.0819504	0.7365325	0.9854749	0.0926791	0.6024763	0.8694628	0.0914365	0.5352981	0.9998749	0.0133541	0.933023	0.9660934
SND1	Mitochondria	0.1026709	0.4044757	0.7366398	0.0065909	0.9758336	0.9970186	0.1557514	0.4098771	0.7656195	0.3156641	0.0464568	0.9998749	0.5440518	0.0004918	0.0129352
SOD1	Peroxisome	-0.2586572	0.0005303	0.0086859	-0.3669319	0.0154386	0.2637425	-0.2093344	0.0407631	0.2802461	-0.1177296	0.4214577	0.9998749	-0.1704306	0.1606294	0.5203616

SOD2	Peroxisome	0.2414787	0.0747011	0.309199	0.5307705	0.0096022	0.1789508	0.0430316	0.817005	0.9493368	0.3280193	0.1730021	0.9998749	0.0348644	0.8285042	0.9660934
SPATA20	Mitochondria	0.1845248	0.496812		-0.0631247	0.8932215		0.3488447	0.362763		0.0979835	0.7493723	0.9998749	0.4557058	0.1854433	
SPG7	Mitochondria	0.2364539	0.1184349	0.4101232	0.1575275	0.5520948	0.9854749	0.290914	0.1895413	0.572091	0.1317442	0.5016311	0.9998749	0.3395778	0.0815855	
SPHK2	Mitochondria	0.2264529	0.5475287		-0.2915849	0.6527124		0.5884662	0.2671116		-0.061345	0.8862544	0.9998749	1.0010567	0.0350647	
SPIRE1	Mitochondria	0.0940358	0.7798689		0.4315116	0.4528274		-0.1497102	0.7535056		0.3423492	0.3983241	0.9998749	0.1001481	0.8335663	
SPR	Mitochondria	0.0913759	0.5771454	0.8642121	-0.0591144	0.8504947	0.9854749	0.1781229	0.4300533	0.7881107	-0.0091721	0.9623256	0.9998749	0.0703205	0.7338856	
SPRYD4	Mitochondria	-0.0430263	0.7594973	0.9456563	-0.2448619	0.3644908	0.9464092	0.0640304	0.7403213	0.922111	-0.3258398	0.0721898	0.9998749	0.0291664	0.8768002	
SPTLC2	Mitochondria	0.4522731	0.0270938	0.1675393	0.6327072	0.078772		0.3026743	0.2891648		0.5340824	0.0223092	0.9998749	0.187859	0.4931207	
SQOR	Mitochondria	0.0539691	0.7666882	0.9456563	-0.0302223	0.9282003		0.0687773	0.7903451	0.9427007	0.0037384	0.9861136	0.9998749	0.1041339	0.6431448	
SSBP1	Mitochondria	0.0691199	0.659457	0.9184946	0.1550629	0.6053483	0.9854749	0.0068318	0.9750958	0.9876706	0.1523257	0.4665627	0.9998749	-0.3021954	0.1365237	
STARD7	Mitochondria	0.1110475	0.30519	0.6869339	0.1245005	0.5672524	0.9854749	0.0710206	0.6471517	0.8948048	0.0950139	0.4824298	0.9998749	0.0719284	0.58102	0.9150195
STOM	Mitochondria	-0.0712647	0.6619237	0.9184946	0.0186751	0.9335456	0.9909626	-0.1287135	0.5412697	0.8452004	0.2623321	0.3890196	0.9998749	0.3145152	0.0492287	0.3157844
STOML2	Mitochondria	-0.0198557	0.9454807		-0.1356926	0.7924424		0.036818	0.9274896		0.2358259	0.468227	0.9998749	0.2794329	0.4342337	
STX17	Mitochondria	-0.1080907	0.3933774	0.73132	0.0881926	0.7095401	0.9854749	-0.2358744	0.1879565	0.572091	-0.009847	0.9493219	0.9998749	-0.2324924	0.1722372	0.5206711
STYXL1	Mitochondria	0.3938507	0.3681144		0.2225114	0.7736298		0.5032835	0.407977		0.1141081	0.8085838	0.9998749	0.2350471	0.6926974	
SUCLA2	Mitochondria	0.0754239	0.7131065	0.9355783	0.2458107	0.5039224		-0.0716809	0.8036843		0.1901694	0.45862	0.9998749	0.0479268	0.8522245	
SUCLG1	Mitochondria	0.2695101	0.0035933	0.0375433	0.231329	0.1917217	0.7860591	0.2748121	0.0498341	0.3083484	0.037018	0.7767952	0.9998749	0.0173727	0.873538	0.9660934
SUCLG2	Mitochondria	-0.422141	1.042E-05	0.0003325	-0.3048318	0.0469242	0.4275315	-0.5164698	0.000401	0.0082713	0.0762143	0.6851359	0.9998749	0.0282184	0.8333881	0.9660934

SUGCT	Mitochondria	-0.2947604	0.2814307		-0.1756033	0.7253748		-0.3861573	0.3093317		0.0546513	0.8685322	0.9998749	-0.0542199	0.8688967	
SUOX	Mitochondria	0.0200503	0.8854366	0.9646758	-0.1721828	0.5064886	0.9854749	0.1295429	0.5077984	0.8234714	-0.0619992	0.7177193	0.9998749	0.238771	0.1604134	
SUPV3L1	Mitochondria	0.3789431	0.1695298		0.5778754	0.254797		0.2482406	0.5015789		0.1452568	0.6299761	0.9998749	-0.2358555	0.5262969	
SURF1	Mitochondria	-0.3916573	0.2187666		-0.7242956	0.2438737		-0.2220983	0.5946287		-0.219043	0.5701505	0.9998749	0.0216025	0.9539545	
SYNJ2BP	Mitochondria	-0.1935763	0.0742087	0.309199	-0.0866118	0.68422	0.9854749	-0.2643919	0.08886	0.4084982	0.0415306	0.7644695	0.9998749	-0.0780941	0.5538456	0.9045021
TACO1	Mitochondria	0.1524948	0.2897838	0.6728314	0.0210888	0.938476	0.9909626	0.2270916	0.266171	0.6427056	0.0327295	0.8438114	0.9998749	0.1821979	0.3242393	
TAMM41	Mitochondria	0.0849357	0.8775817		0.207913	0.8213892		-0.0229713	0.9768828		0.576214	0.3399821	0.9998749	0.2431918	0.7313127	
TARS2	Mitochondria	0.1851044	0.5627716		0.0320269	0.9570211		0.2665946	0.5329115		0.0645402	0.8578954	0.9998749	0.0481819	0.906694	
TAZ	Mitochondria	0.9019546	0.1600816		1.3335756	0.2174139		0.6171394	0.4858102		0.3932964	0.5547657	0.9998749	0.0049987	0.9957333	
TBRG4	Mitochondria	-0.1090005	0.8384525		-0.0267018	0.9778883		-0.1921419	0.7931453		0.4402654	0.4583971	0.9998749	0.145157	0.8272825	
TCAIM	Mitochondria	-0.2401824	0.2716629	0.6532846	-0.2370544	0.5680506		-0.2563563	0.3893165		-0.2583424	0.336049	0.9998749	-0.231579	0.3947793	
TDRKH	Mitochondria	0.105842	0.8498971		0.2921955	0.7550516		-0.0386591	0.9615193		0.65815	0.2696795	0.9998749	0.2419224	0.7343611	
TEFM	Mitochondria	0.3006036	0.4242176		0.2287749	0.7430299		0.3206276	0.5290354		0.0798321	0.8454948	0.9998749	0.0278811	0.9555403	
TFAM	Mitochondria	0.0281812	0.8755037	0.9646758	0.2000517	0.5448749		-0.0953072	0.7052808	0.9139984	0.22977	0.2702359	0.9998749	-0.0312054	0.8909884	
TFB1M	Mitochondria	0.3296393	0.1656815		0.4882189	0.2779909		0.2217405	0.4844979		-0.0424807	0.8777113	0.9998749	-0.3352987	0.2947417	
TFB2M	Mitochondria	-0.2399326	0.2473172	0.6214448	-0.2862435	0.4595187		-0.2352465	0.4160988		-0.0504648	0.8392942	0.9998749	0.0009866	0.9968722	
THEM4	Mitochondria	0.2222617	0.3114774	0.6939534	0.4395293	0.2797013		0.0814216	0.782447		0.0564558	0.8262162	0.9998749	-0.2010927	0.4742913	
THG1L	Mitochondria	-0.0573983	0.8673745		-0.1313458	0.8132865		-0.0359846	0.9441499		0.4400094	0.2550396	0.9998749	0.5254135	0.2201586	

THNSL1	Mitochondria	-0.5836333	0.0017506	0.0230621	-0.4589666	0.1780289		-0.6942723	0.0081556	0.0962193	-0.0341542	0.8879391	0.9998749	-0.1679212	0.4198109	
TIMM10	Mitochondria	0.0673195	0.7685828		-0.1626267	0.7129488		0.1881866	0.5404529		-0.1154379	0.6662629	0.9998749	-0.0041807	0.9886752	
TIMM10B	Mitochondria	0.0038301	0.984406	0.9918134	0.132968	0.711461		-0.1037839	0.705344		0.3141177	0.1682472	0.9998749	0.0731456	0.7660489	
TIMM13	Mitochondria	0.2799235	0.1699225	0.499869	0.0707411	0.8170839	0.9854749	0.4032324	0.1498913	0.5152579	0.0110163	0.9710838	0.9998749	-0.1612962	0.5818007	
TIMM17A	Mitochondria	0.1763212	0.2761342	0.6588083	0.0704699	0.8194636	0.9854749	0.2197083	0.3258714	0.7013319	0.0610116	0.7549551	0.9998749	0.094637	0.6530392	
TIMM17B	Mitochondria	0.3354288	0.4306496		0.2190364	0.7855629		0.3984132	0.4774023		-0.0247061	0.9571518	0.9998749	-0.1411351	0.8090446	
TIMM21	Mitochondria	-0.1816376	0.37607	0.731203	-0.0892761	0.8166372		-0.2495132	0.3792744		0.0515722	0.8330145	0.9998749	-0.0867283	0.7320492	
TIMM22	Mitochondria	0.298505	0.4817732		0.1021438	0.892135		0.4110241	0.4847547		0.2104681	0.6419131	0.9998749	0.2430528	0.6701833	
TIMM23	Mitochondria	0.1838897	0.3661369	0.7245031	0.2274972	0.5386744		0.1404214	0.6253763		0.2481055	0.2768076	0.9998749	0.0678341	0.7945716	
TIMM29	Mitochondria	-0.2532315	0.6249693		-0.1582618	0.8600133		-0.356642	0.6259651		0.3278468	0.5923037	0.9998749	0.1758056	0.7822945	
TIMM44	Mitochondria	0.5027161	0.020466	0.1305518	0.3133535	0.4045228		0.612455	0.0407466	0.2802461	0.1282955	0.6145701	0.9998749	0.2100097	0.4492372	
TIMM50	Mitochondria	0.1746859	0.3985052	0.7362626	-0.0551109	0.8860032		0.3117503	0.2731239		-0.0533199	0.8226679	0.9998749	0.1920021	0.4684263	
TIMM8A	Mitochondria	-0.1829698	0.5392255		-0.2459609	0.673375		-0.1671996	0.6728505		-0.2095859	0.5615456	0.9998749	-0.3018453	0.4200369	
TIMM8B	Mitochondria	-0.0802263	0.7110288	0.9355783	-0.3614568	0.3726851		0.0753251	0.7966064		0.3186895	0.3142644	0.9998749	0.0656608	0.803187	
TIMM9	Mitochondria	0.0175891	0.9591774		-0.0246424	0.9688706		0.0335628	0.9429649		0.058906	0.882802	0.9998749	-0.0816079	0.8553723	
TIMMDC1	Mitochondria	0.0774778	0.5853863	0.8642121	0.1511119	0.5812968	0.9854749	0.0269401	0.8917586	0.9722587	0.022144	0.8951353	0.9998749	-0.1592451	0.3739262	
TK2	Mitochondria	0.4189229	0.0646175	0.2849268	0.4194452	0.31662		0.401793	0.1964585		-0.1052888	0.6826648	0.9998749	-0.0014424	0.9962411	
TMEM11	Mitochondria	0.2978256	0.2427058		0.3430309	0.4645877		0.2601573	0.4570521		0.1861839	0.5032649	0.9998749	0.018961	0.9553175	
TMEM126A	Mitochondria	0.0166155	0.9633084		-0.0159724	0.980924		0.0028774	0.9953412		-0.0210071	0.958883	0.9998749	-0.031631	0.9439983	

TMEM126B	Mitochondria	0.0730508	0.8389281		0.3899572	0.5635413		-0.11223	0.8152606		0.0835422	0.8334625	0.9998749	-0.4294865	0.3649359	
TMEM143	Mitochondria	0.2348301	0.6459337		0.454583	0.617243		0.1034984	0.8808856		0.151596	0.7961043	0.9998749	-0.0961295	0.8871757	
TMEM14C	Mitochondria	-0.1436481	0.3799619	0.731203	-0.0407801	0.8964963	0.9854749	-0.2220413	0.3296854	0.7027967	0.021083	0.9132702	0.9998749	-0.1738298	0.3882911	
TMEM177	Mitochondria	0.3868798	0.2003411		0.2110729	0.706962		0.4898054	0.227185		-0.099813	0.7608727	0.9998749	-0.0335777	0.9349348	
TMEM186	Mitochondria	0.0082298	0.9794405		-0.200203	0.7441889		0.1145895	0.7862101		0.0485529	0.8955003	0.9998749	-0.0404374	0.9210802	
TMEM205	Mitochondria	0.1611879	0.3656698	0.7245031	0.0221868	0.9461419		0.2419536	0.3351636	0.7027967	-0.0287589	0.8897288	0.9998749	0.1834679	0.437755	
TMEM65	Mitochondria	-0.0655813	0.8339399		0.343497	0.5241134		-0.3634506	0.4077164		0.5890653	0.1002926	0.9998749	-0.0450517	0.9096449	
TMEM70	Mitochondria	0.0335981	0.8762961	0.9646758	0.0507649	0.8955498		-0.0060638	0.9842871		0.0205875	0.9339797	0.9998749	0.1445515	0.592864	
TMLHE	Mitochondria	-0.2732923	0.2044882	0.5552542	-0.2358936	0.5510232		-0.3143603	0.2985467		0.1000746	0.7042963	0.9998749	-0.0217231	0.9348378	
TOMM20	Mitochondria	-0.3053275	0.0022254	0.0259344	-0.1613745	0.4270925	0.9506771	-0.4073991	0.0035444	0.0531655	0.0819634	0.5233307	0.9998749	-0.1036373	0.3914196	0.7798739
TOMM22	Mitochondria	-0.027186	0.8547379	0.9633793	0.0101252	0.9718186	0.9970186	-0.0665794	0.7507581	0.922111	0.1242434	0.4755102	0.9998749	-0.045378	0.8072731	
TOMM34	Mitochondria	0.2034633	0.4668587		0.277568	0.5820344		0.1264449	0.7462155		0.2361195	0.4490225	0.9998749	0.1478655	0.6832072	
TOMM40	Mitochondria	0.2991872	0.409571		0.058382	0.931664		0.4290215	0.3779084		-0.2564713	0.5360109	0.9998749	0.0537661	0.9122353	
TOMM40L	Mitochondria	0.2035221	0.4456584		0.1285375	0.7966095		0.2270321	0.5240704		-0.076448	0.8109645	0.9998749	-0.0295309	0.9321609	
TOMM5	Mitochondria	0.2121924	0.1142108	0.4071278	0.3910761	0.1340298	0.6874549	0.0991032	0.5973361	0.8647185	0.1430143	0.3968346	0.9998749	-0.3272211	0.0615702	
TOMM6	Mitochondria	0.1415324	0.3640788	0.7245031	0.1318883	0.6542383	0.9854749	0.1419027	0.5172454	0.8259241	-0.0639258	0.723541	0.9998749	-0.0023276	0.9908472	
TOMM7	Mitochondria	-0.1695673	0.1967578	0.5429961	-0.1920614	0.438239	0.9506771	-0.1705282	0.3535263	0.7113638	0.2357428	0.2962222	0.9998749	-0.1505222	0.3399448	0.7513066
TOMM70	Mitochondria	-0.0949391	0.3392445	0.713827	-0.0451165	0.8153647	0.9854749	-0.1513614	0.3134799	0.6998859	0.0172696	0.8967204	0.9998749	-0.0512588	0.6896022	0.9365555
TOP1MT	Mitochondria	0.0821681	0.7009076	0.9340525	-0.0658722	0.8642811		0.1772195	0.5518434		-0.0390309	0.8736919	0.9998749	0.2057107	0.449919	
TOP3A	Mitochondria	0.1149286	0.7327965		0.2558328	0.6601381		0.0035554	0.9940134		0.307202	0.4267151	0.9998749	0.1505475	0.7269504	

TRAP1	Mitochondria	0.1824756	0.18443	0.5277187	0.0907187	0.7282076	0.9854749	0.2349909	0.2320524	0.620897	-0.0525504	0.7525542	0.9998749	0.0594321	0.7424003	
TRIAP1	Mitochondria	-0.0120138	0.9320187	0.981362	-0.2036635	0.4615271	0.966675	0.0869125	0.6506603	0.8971499	-0.0953066	0.5925184	0.9998749	0.0087054	0.9597286	
TRIT1	Mitochondria	0.0459125	0.9229558		0.0840501	0.9223109		0.0096157	0.9879551		0.087463	0.8760935	0.9998749	-0.106016	0.8623368	
TRMT1	Mitochondria	0.259075	0.5856681		-0.0650423	0.9386836		0.46908	0.4696895		-0.2064782	0.7024643	0.9998749	0.289405	0.6439973	
TRMT10C	Mitochondria	0.4402344	0.0158244	0.1042347	0.4781957	0.1325616	0.6874549	0.4047662	0.1030762	0.4475676	0.1287262	0.6267507	0.9998749	-0.143453	0.521717	
TRMT2B	Mitochondria	0.0023015	0.9963297		-0.1636628	0.8622281		0.0712043	0.9139281		-0.2195945	0.718735	0.9998749	-0.0633979	0.9211781	
TRMT5	Mitochondria	0.0066704	0.9784922		0.1474036	0.738222		-0.0943895	0.7853796		0.2540542	0.373521	0.9998749	0.0194002	0.9509591	
TRMT61B	Mitochondria	-0.1591459	0.633339		-0.188393	0.7562575		-0.1794932	0.6970985		0.0544249	0.888578	0.9998749	0.0580032	0.8868851	
TRMU	Mitochondria	-0.1211887	0.8060082		-0.013313	0.9878981		-0.2254322	0.7370689		-0.0180182	0.9751223	0.9998749	-0.0173915	0.9775025	
TRNT1	Mitochondria	0.0652178	0.8217244		0.2644325	0.6043207		-0.0891901	0.8259663		0.3109478	0.3422924	0.9998749	-0.0556968	0.8802828	
TRUB2	Mitochondria	0.0594803	0.7893139	0.9456563	0.0119757	0.9772199		0.0797223	0.7920575		-0.0596613	0.8203	0.9998749	-0.0775624	0.786196	
TSFM	Mitochondria	0.312047	0.1621102	0.499869	0.2584354	0.5276313		0.3394844	0.2712227		0.0572579	0.814164	0.9998749	0.1759995	0.5480947	
TSPO	Mitochondria	0.5362772	0.4814715		-0.0947761	0.9277876		1.0190024	0.4614599		0.8002049	0.2570937	0.9998749	1.4986193	0.0879281	
TST	Mitochondria	0.1439561	0.3634616	0.7245031	-0.2446442	0.3942165	0.9464092	0.3773276	0.0712073	0.3633772	0.121387	0.5716582	0.9998749	0.4952093	0.0168691	
TSTD1	Mitochondria	-0.0569156	0.8381522		0.0596429	0.9112455		-0.1309392	0.7235308		0.3255625	0.3238795	0.9998749	-0.295301	0.4110148	
TSTD3	Mitochondria	-0.2199955	0.6989332		0.6189693	0.5548453		-0.6880561	0.3667362		0.4096365	0.5342982	0.9998749	-0.4849407	0.5045179	
TTC19	Mitochondria	-0.1387685	0.2829608	0.6620628	0.0567522	0.822386	0.9854749	-0.2684561	0.1357405	0.5020154	0.1059961	0.5040689	0.9998749	-0.2148667	0.1846261	0.5395184
TUFM	Mitochondria	-0.0140701	0.9090949	0.96821	-0.1146376	0.5797553	0.9854749	0.0386815	0.8159155	0.9493368	0.0307623	0.8666196	0.9998749	0.1942856	0.3807806	0.7798739

TWNK	Mitochondria	-0.072665	0.884305		0.0856212	0.9244491		-0.2109048	0.7570102		0.1892974	0.7410422	0.9998749	-0.0623583	0.9224673	
TXN2	Mitochondria	0.0784905	0.5656688	0.8569882	-0.0349277	0.8935566	0.9854749	0.1465795	0.4509121	0.7923079	-0.1376547	0.3921498	0.9998749	-0.0144197	0.9345837	
TXNRD1	Mitochondria	0.388947	0.0771973	0.3139701	0.4977743	0.1979224		0.2931458	0.3524415		0.3258861	0.189968	0.9998749	0.1974171	0.5007651	
TXNRD2	Mitochondria	0.2927644	0.534721		-0.0640194	0.9396659		0.5135592	0.4288839		-0.2507129	0.6413536	0.9998749	0.4095318	0.5068923	
UCP2	Mitochondria	1.1562008	0.0008611		1.625358	0.0056827		0.843338	0.0782698		0.5324074	0.0978816	0.9998749	-0.134297	0.7993309	
UNG	Mitochondria	-0.190313	0.385968	0.731203	-0.3399501	0.4077194		-0.1202741	0.6899494		-0.1292516	0.6400284	0.9998749	0.0968176	0.7150273	
UQCC1	Mitochondria	0.0502563	0.8349684		0.2385455	0.5777781		-0.1011153	0.767206		0.2570241	0.3549453	0.9998749	0.0452818	0.8811791	
UQCC2	Mitochondria	0.2895288	0.1998466		0.1242115	0.7621692		0.3867938	0.2194034		0.2715166	0.2852926	0.9998749	0.2505283	0.398841	
UQCC3	Mitochondria	0.1333277	0.7209339		-0.1975102	0.7722166		0.3517325	0.4900401		-0.0784747	0.8473475	0.9998749	0.1899755	0.6990997	
UQCR10	Mitochondria	0.1534709	0.4138705	0.7420282	0.0241316	0.9448248		0.2222548	0.3984454	0.7588581	0.2836384	0.2493876	0.9998749	0.2134835	0.3738596	
UQCR11	Mitochondria	0.1421842	0.3787498	0.731203	-0.1363503	0.6362203	0.9854749	0.3118351	0.1674411	0.5525557	0.1420327	0.5500322	0.9998749	0.1271824	0.5916252	
UQCRB	Mitochondria	0.1061333	0.2991932	0.6841928	0.0756218	0.6431476	0.9854749	0.1128115	0.4386424	0.7881107	0.2095355	0.37235	0.9998749	-0.1822905	0.1444663	0.4871108
UQCRC1	Mitochondria	0.0704749	0.5825728	0.8642121	-0.1118418	0.612881	0.9854749	0.1774367	0.3388833	0.7027967	0.1033126	0.5369134	0.9998749	0.3581523	0.0657654	0.3760063
UQCRC2	Mitochondria	0.0317235	0.7454418	0.9456563	0.0207303	0.9142562	0.9909626	0.009833	0.94688	0.9832552	0.1576548	0.244535	0.9998749	0.1631719	0.174545	0.5216517
UQCRFS1	Mitochondria	0.1575182	0.230698	0.6000128	0.0516325	0.839512	0.9854749	0.1962085	0.2947528	0.684413	0.0355454	0.826188	0.9998749	0.0618868	0.7067512	
UQCRH	Mitochondria	0.3655825	0.0032693	0.0353786	0.341997	0.1015919	0.6091981	0.3641044	0.0414316	0.2809403	0.2683085	0.2066354	0.9998749	0.0207926	0.9151904	0.9660934
UQCRQ	Mitochondria	0.0167804	0.8962252	0.9646758	-0.1157547	0.5931049	0.9854749	0.0810421	0.6147902	0.8714138	0.5088971	0.0842524	0.9998749	-0.083394	0.6501428	0.9365555
USP30	Mitochondria	-0.363211	0.1388565		-0.395219	0.3960702		-0.371592	0.264904		-0.1886621	0.536973	0.9998749	-0.1485222	0.6079581	
VARS2	Mitochondria	0.1037368	0.7694752		0.0208783	0.9740948		0.154618	0.7488732		-0.061232	0.8812183	0.9998749	0.2357585	0.599357	

VDAC1	Mitochondria	-0.0430631	0.7680938	0.9456563	-0.0629099	0.8082761	0.9854749	-0.0520648	0.8062091	0.9493368	-6.422E-05	0.9997188	0.9998749	-0.0374079	0.853322	0.9660934
VDAC2	Mitochondria	0.055943	0.6769607	0.9184946	0.1001766	0.7024544	0.9854749	0.0094137	0.9604254	0.9876706	-0.0099101	0.9493916	0.9998749	-0.1707036	0.3208388	
VDAC3	Mitochondria	0.1086003	0.5549251	0.8428185	0.2013146	0.5561974		0.0336673	0.8964845	0.9722587	0.0220063	0.9171886	0.9998749	-0.0499012	0.8357457	
VPS13D	Mitochondria	-0.1866212	0.249574	0.6223944	-0.0977939	0.7353882	0.9854749	-0.2578787	0.2712674	0.6493035	0.2285914	0.2540845	0.9998749	0.1940615	0.3148867	
VWA8	Mitochondria	-0.3075308	0.012768	0.0889356	-0.0928481	0.6838714	0.9854749	-0.4722728	0.0082193	0.0962193	0.2186358	0.2251131	0.9998749	-0.0207517	0.8870901	0.9660934
WARS2	Mitochondria	-0.0765987	0.8414952		-0.0887795	0.8974624		-0.0705602	0.8930404		0.0997616	0.8223303	0.9998749	0.0713017	0.8817588	
XPNPEP3	Mitochondria	-0.300532	0.1089831	0.3949793	-0.1701803	0.627078		-0.3862082	0.1358991	0.5020154	0.0081298	0.9721093	0.9998749	-0.1225447	0.5832769	
YARS2	Mitochondria	0.1625007	0.6742255		0.3097187	0.6721878		0.0753637	0.883571		-0.0417039	0.9236117	0.9998749	-0.2145018	0.6759832	
YBEY	Mitochondria	-0.2246277	0.6005747		-0.3237401	0.6627415		-0.1827739	0.7627145		0.2183621	0.6570522	0.9998749	0.3708984	0.4720141	
YME1L1	Mitochondria	0.1112044	0.4010061	0.7366398	0.2550655	0.2935342	0.9358893	-0.0068868	0.9709299	0.9876706	0.3855369	0.0275583	0.9998749	0.1857499	0.2579069	0.6603866
YRDC	Mitochondria	0.0194267	0.9615507		0.0235565	0.9746701		-0.0068433	0.9900334		-0.0257454	0.9567076	0.9998749	0.0017789	0.997236	
ZADH2	Mitochondria	-0.1286413	0.3889616	0.73132	0.0029011	0.9921615	0.9970186	-0.2151738	0.2899609	0.6806876	-0.0982139	0.5902458	0.9998749	-0.3638778	0.0517482	
ABCD4	Peroxisome	0.0629235	0.8257646		-0.2500696	0.6395759		0.2237005	0.5519806		-0.1483958	0.6675697	0.9998749	-0.0375878	0.9160413	
ACOT8	Peroxisome	0.0945262	0.7936646		-0.0318867	0.9597379		0.1728463	0.7349773		0.3524834	0.3758633	0.9998749	0.336829	0.4685497	
ACOX1	Peroxisome	-0.4978999	7.553E-07	2.953E-05	-0.387237	0.0304617	0.3467332	-0.5815411	6.672E-05	0.0027521	-0.0278712	0.8615645	0.9998749	-0.0864137	0.4135609	0.7885242
ACOX2	Peroxisome	-0.1653382	0.0547237	0.2553494	-0.1513061	0.3831258	0.9464092	-0.1734975	0.1674153	0.5525557	-0.1236511	0.3897929	0.9998749	-0.2112447	0.1314951	0.4871108
ACOX3	Peroxisome	0.0097928	0.9762806		-0.2183205	0.72464		0.1362467	0.7566046		-0.2705485	0.4862518	0.9998749	0.0819769	0.8448183	
ACSL3	Peroxisome	0.2396602	0.1666205	0.499869	0.4284068	0.1458217	0.7094408	0.0939778	0.6946263	0.9088278	0.3403276	0.1483181	0.9998749	0.0812003	0.6955805	

ACSL4	Peroxisome	1.6009556	1.804E-07	8.409E-06	1.4285469	5.901E-05	0.0048386	1.7156431	4.56E-05	0.0020521	-0.1729646	0.7992347	0.9998749	1.4314729	0.0195408	
ACSL5	Peroxisome	-0.2838436	0.0112565	0.0821862	-0.2332319	0.2530777	0.9182464	-0.3199278	0.0429383	0.2872227	-0.1386838	0.3751326	0.9998749	-0.1053339	0.5228646	0.890164
AGPS	Peroxisome	0.0104859	0.9494848	0.9835689	0.1281518	0.6748164	0.9854749	-0.093008	0.6846107	0.9036861	0.1068603	0.6238336	0.9998749	0.0643755	0.7572419	
BAAT	Peroxisome	-0.1807305	0.0917753	0.3588117	-0.1903228	0.3265929	0.9358893	-0.1875912	0.1331554	0.5007432	-0.4113113	0.0252961	0.9998749	-0.4776417	0.010142	0.1111389
DAO	Peroxisome	-0.2245461	0.1027131	0.3804901	-0.4311587	0.0908095	0.5998205	-0.1115195	0.5597247	0.8510009	-0.0373146	0.8241204	0.9998749	0.2429045	0.1359166	0.4871108
DDO	Peroxisome	0.6745786	0.0536148		0.5916323	0.3279309		0.7193886	0.1434462		0.1388958	0.699124	0.9998749	0.2756363	0.5630453	
DECR2	Peroxisome	0.3382271	0.0253622	0.1584483	0.2263917	0.3826725	0.9464092	0.4127422	0.0594624	0.3457949	-0.1986339	0.2954325	0.9998749	0.0880448	0.731841	
FAR1	Peroxisome	0.3188997	0.282528		0.572641	0.24445		0.116588	0.7759852		0.7310129	0.0215797	0.9998749	0.3806778	0.3382051	
FAR2	Peroxisome	-0.0617947	0.9136579		0.5585842	0.5650817		-0.4985194	0.5390306		0.7850879	0.2090573	0.9998749	-0.0613064	0.9325302	
GNPAT	Peroxisome	0.2640596	0.0987014	0.3738315	0.4091237	0.1563776	0.7123869	0.1608264	0.4712673	0.798895	0.1970561	0.3029809	0.9998749	-0.0667315	0.7400629	
HACL1	Peroxisome	-0.0326585	0.8872706	0.9646758	-0.0853012	0.8417005		-0.0028873	0.992489		0.2018231	0.4553254	0.9998749	-0.0763456	0.7898145	
HAO1	Peroxisome	-0.0984702	0.1635269	0.499869	-0.0623915	0.6773984	0.9854749	-0.1296846	0.2037847	0.5968843	-0.2106859	0.1062249	0.9998749	-0.2957272	0.0038656	0.0598036
IDH1	Peroxisome	-0.4740284	4.764E-05	0.001203	-0.3276233	0.0626495	0.469108	-0.5782764	0.0003828	0.0082392	0.0479626	0.813675	0.9998749	0.0074143	0.9472527	0.9693676
MVK	Peroxisome	0.1976818	0.5710335		0.053344	0.9347253		0.2648608	0.5621577		-0.0828425	0.8400599	0.9998749	0.0924463	0.8329165	
NUDT12	Peroxisome	-0.3142522	0.0971312	0.3725412	-0.0950667	0.7795103		-0.4729881	0.0773993	0.3719676	0.1463628	0.5451973	0.9998749	-0.1227505	0.6172211	
NUDT7	Peroxisome	-0.1493032	0.5640245		0.1033069	0.8330978		-0.2946996	0.3931483		0.0193682	0.9486478	0.9998749	-0.4740204	0.1476382	
PAOX	Peroxisome	0.1447515	0.8339236					0.0079765	0.9928574		0.0331709	0.9639627	0.9998749	-0.3317533	0.7072286	
PECR	Peroxisome	-0.2113768	0.0182736	0.1178067	-0.1797555	0.3328502	0.9358893	-0.2418597	0.0694506	0.3581046	0.0113789	0.9284549	0.9998749	0.029084	0.7851233	0.9587045
PEX1	Peroxisome	-0.2883218	0.2158455		0.0922617	0.8337904		-0.5096965	0.1040404		0.0259724	0.9273662	0.9998749	-0.4608504	0.1150228	

PEX10	Peroxisome	-0.1730017	0.6640177		-0.0713097	0.9275378		-0.2553249	0.6255964		-0.3048502	0.5251105	0.9998749	-0.3761781	0.4570208	
PEX11A	Peroxisome	-0.0717564	0.7173074	0.9388516	-0.0600878	0.876658		-0.0846904	0.7495588	0.922111	-0.2012763	0.4020449	0.9998749	-0.28849	0.2517053	
PEX11G	Peroxisome	-0.1941838	0.7190542		-0.0302336	0.9757789		-0.2973096	0.6824976		-0.2275811	0.7352165	0.9998749	-0.1093943	0.8717667	
PEX12	Peroxisome	-0.1185239	0.6866306		-0.1297812	0.8063919		-0.13456	0.7434021		0.0048251	0.9890968	0.9998749	0.0809962	0.8243259	
PEX13	Peroxisome	-0.1664772	0.1849182	0.5277187	-0.0631993	0.7983596	0.9854749	-0.2416892	0.1755748	0.5578491	-0.0760052	0.6255071	0.9998749	-0.1350944	0.3820776	0.7798739
PEX14	Peroxisome	0.3587998	0.062104	0.278778	0.3073963	0.3844652		0.3940173	0.1425883	0.5098188	-0.1492583	0.513632	0.9998749	0.0351593	0.8980812	
PEX16	Peroxisome	0.0756359	0.8224034		-0.1551099	0.8090734		0.2016933	0.6534398		-0.2278283	0.5529188	0.9998749	0.0659609	0.878354	
PEX19	Peroxisome	0.0143595	0.9373731	0.981362	0.0106614	0.9751844		-0.0017296	0.9946164	0.9946164	-0.0678752	0.7529386	0.9998749	-0.0264714	0.9088751	
PEX2	Peroxisome	-0.0158571	0.9558952		0.1054214	0.8389339		-0.1144136	0.7741655		0.0772725	0.816021	0.9998749	-0.0214621	0.9530988	
PEX26	Peroxisome	0.2510133	0.3566172		0.2314799	0.6292926		0.2369858	0.5391446		0.3128332	0.3125868	0.9998749	0.2779292	0.4338122	
PEX3	Peroxisome	0.0223882	0.910769	0.9682913	0.0547119	0.8816933		-0.0358307	0.8986715		0.0329501	0.8907049	0.9998749	0.0526962	0.831482	
PEX5	Peroxisome	-0.2315983	0.5378916		-0.1585425	0.8240211		-0.2662103	0.5940051		-0.0864048	0.8488032	0.9998749	-0.339105	0.4770169	
PEX6	Peroxisome	0.012597	0.9652293		0.0602957	0.8987602		-0.0240253	0.9547545		0.337864	0.315653	0.9998749	0.4890845	0.1695169	
PEX7	Peroxisome	0.1377834	0.7239093		0.1355467	0.8459709		0.1211935	0.8219849		0.1224888	0.7791462	0.9998749	0.005703	0.9911497	
PIPOX	Peroxisome	-0.1512194	0.0442218	0.2271051	-0.322987	0.0288732	0.3467332	-0.0634918	0.5323302	0.841864	-0.2031468	0.0358357	0.9998749	0.0537363	0.6170906	0.9365555
PMVK	Peroxisome	-0.1601788	0.4408844	0.7513739	-0.1933674	0.6192829		-0.1559822	0.5882725		0.0170795	0.9448299	0.9998749	-0.0244201	0.9272597	
PRDX1	Peroxisome	0.0059562	0.9692874	0.988921	0.0526123	0.8016485	0.9854749	-0.0339057	0.8663149	0.9673117	0.0689504	0.7601065	0.9998749	0.0903483	0.6662278	0.9365555

SLC25A17	Peroxisome	0.0025459	0.9943391		-0.3873178	0.5520199		0.2379914	0.6242452		-0.2901561	0.5035647	0.9998749	0.2392812	0.5940473	
SLC27A2	Peroxisome	0.0895056	0.3600082	0.7245031	0.2305631	0.195676	0.7865408	-0.0054258	0.9679672	0.9876706	0.1030008	0.5490112	0.9998749	-0.2678989	0.1642206	0.5203616
XDH	Peroxisome	0.0939064	0.4042709	0.7366398	0.1638483	0.406811	0.9464092	0.041912	0.7835803	0.9368895	0.0212451	0.9136052	0.9998749	-0.0773424	0.5282587	0.89059

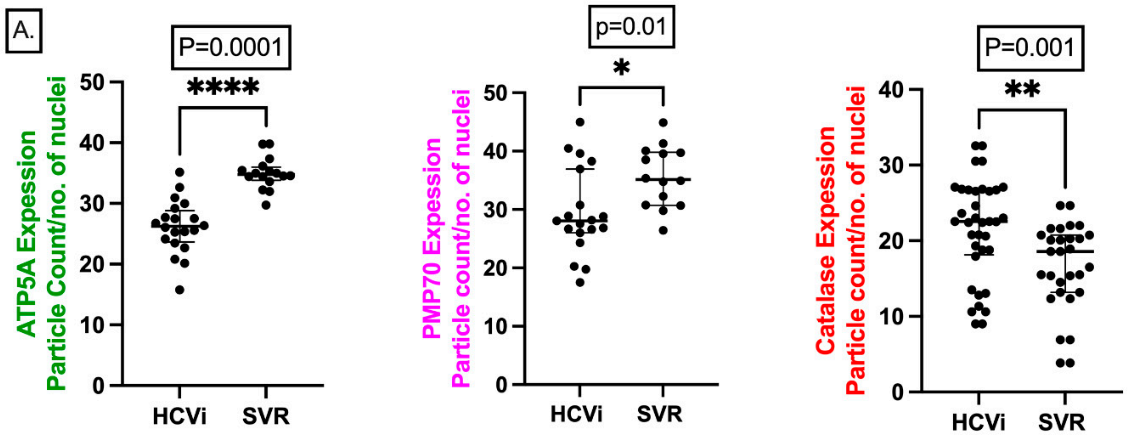

Supplementary Figure S1: Quantification of particle count in the hepatocyte for ATP5A, PMP70, and catalase expression across HCVi and SVR groups. All the images were normalized to the total number of nuclei. Mann-Whitney U test ($p<0.05$) was used for marker comparison between groups. *$p<0.05$, **$p<0.01$, ***$p<0.001$.

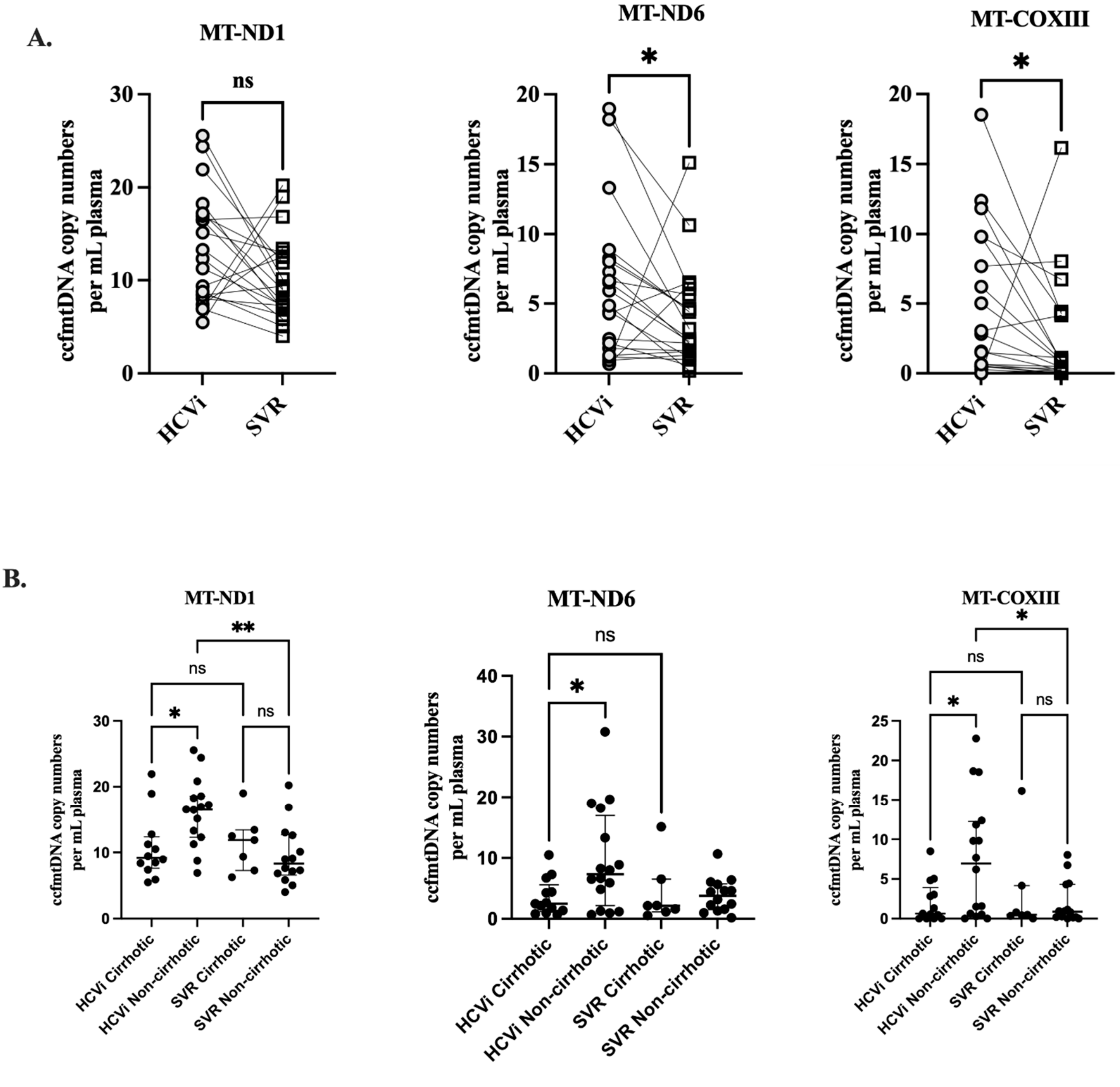

Supplementary Figure S2: Different regions from mtDNA genome have been amplified using target specific primers (MT-ND1, MT-ND6, MT-COXIII) and copy numbers were calculated using standard curves for specific templates. (A) Individual ccfmtDNA copy numbers of MT-ND1, MT-ND6 and MT-COXIII HCVi compared to SVR. (B) ccfmtDNA copy numbers in all the four groups 1) HCVi Cirrhotic, 2) HCVi non-cirrhotic, 3) SVR Cirrhotic, 4) SVR Non-cirrhotic. β-actin was used as control gene. P-values calculated using Wilcoxon test for paired samples and multiple comparisons using one-way ANOVA followed by the Bonferroni correction was used. * $p < 0.05$, ** $p < 0.01$.

Supplementary Table S2: Expression of mitochondrial and peroxisomal transcriptional/regulatory factors captured in the analysis set (HCVi vs. SVR). Differential expression of the intrinsic mitochondrial transcription machinery, mitochondrial functional regulators, and peroxisome-proliferation factors present in the 1094 gene mitochondrial (MitoCarta 3.0) and peroxisomal (KEGG) inventory. Values are shown for the three comparisons: all patients (HCVi vs. SVR), cirrhotic only, and non-cirrhotic only. No gene met the differential-expression threshold (FDR < 0.1 and $|\log_2 FC|$> 0.5 in any comparison.

Gene	Organelle	Regulatory role	HCVi vs. SVR (all $\log_2$FC)	FDR	Cirrhotic $\log_2$FC	FDR	Non-Cirrhotic $\log_2$FC	FDR
TFAM	Mitochondria	mtDNA transcription & packaging	+0.03	0.965	+0.20	NA	-0.10	0.914
TFB1M	Mitochondria	mtDNA transcription factor B1	+0.33	NA	+0.49	NA	+0.22	NA
TFB2M	Mitochondria	mtDNA transcription factor B2	-0.24	0.621	-0.29	NA	-0.24	NA
POLRMT	Mitochondria	Mitochondrial RNA polymerase	+0.16	NA	-0.14	NA	+0.35	NA
TEFM	Mitochondria	Transcription elongation factor	+0.30	NA	+0.23	NA	+0.32	NA
MTERF3	Mitochondria	Transcription termination factor	+0.08	NA	+0.21	NA	-0.00	NA
MTERF4	Mitochondria	Transcription termination factor	+0.10	0.894	+0.05	NA	+0.12	NA
LRPPRC	Mitochondria	mt transcript stability/processing	-0.30	0.098	-0.18	0.946	-0.40	0.195
SLIRP	Mitochondria	mt mRNA stability (LRPPRC partner)	+0.03	0.965	-0.03	NA	+0.05	0.958
SIRT3	Mitochondria	Deacetylase (functional regulator)	-0.07	NA	-0.09	NA	-0.07	NA
PEX 11A	Peroxisome	Peroxisome proliferation	-0.07	0.939	-0.06	NA	-0.09	0.922
PEX 11B	Peroxisome	Peroxisome proliferation	+0.06	NA	-0.07	NA	+0.12	NA
PEX 11G	Peroxisome	Peroxisome proliferation	-0.19	NA	-0.03	NA	-0.30	NA

Note: $\log_2$FC, $\log_2$ fold change (HCVi relative to SVR); FDR, false discovery rate adjusted p-value (DESeq2). NA indicates that an adjusted p value was not returned by DESeq2 (independent filtering / low counts). A gene was considered differentially expressed only when FDR < 0.1 and $|\log_2 FC| > 0.5$.

Abbreviations: TFAM, transcription Factor A, mitochondrial; TFB1M, transcription factor B1, mitochondrial; transcription factor B2, mitochondrial; POLRMT, RNA polymerase mitochondrial; TEFM, transcription elongation factor, mitochondrial; MTERF3, mitochondrial transcription termination factor 3; MTERF4, mitochondrial transcription termination factor 4; LRPPRC, leucine rich pentatricopeptide repeat containing; SLIRP, SRA stem-loop interacting RNA binding protein; SIRT3, sirtuin3; PEX 11A, peroxisomal biogenesis factor 11 Alpha; PEX 11B, peroxisomal biogenesis factor 11 beta; PEX 11G, peroxisomal biogenesis factor 11 gamma.
